# Tripodal, Squaramide-Based Ion Pair Receptor for Effective Extraction of Sulfate Salt

**DOI:** 10.3390/molecules26092751

**Published:** 2021-05-07

**Authors:** Damian Jagleniec, Marcin Wilczek, Jan Romański

**Affiliations:** Faculty of Chemistry, University of Warsaw, Pasteura 1, 02-093 Warsaw, Poland; djagleniec@chem.uw.edu.pl (D.J.); wilczek@chem.uw.edu.pl (M.W.)

**Keywords:** ion pair receptors, tripodal receptors, squaramide, sulfate extraction

## Abstract

Combining three features—the high affinity of squaramides toward anions, cooperation in ion pair binding and preorganization of the binding domains in the tripodal platform—led to the effective receptor **2**. The lack of at least one of these key elements in the structures of reference receptors **3** and **4** caused a lower affinity towards ion pairs. Receptor **2** was found to form an intramolecular network in wet chloroform, which changed into inorganic–organic associates after contact with ions and allowed salts to be extracted from an aqueous to an organic phase. The disparity in the binding mode of **2** with sulfates and with other monovalent anions led to the selective extraction of extremely hydrated sulfate anions in the presence of more lipophilic salts, thus overcoming the Hofmeister series.

## 1. Introduction

Due to the environmental and biological significance of anions, the recognition of this species is one of the main trends of research in the field of supramolecular chemistry. A particular challenge lies in the selective recognition of anions in highly competitive aqueous media [1,2]. A special case is sulfate anions [3] for their growing importance in areas such as industry [4], medicine [5,6] and radioactive waste utilization [7,8]. However, one of the main problems is that sulfate anions are characterized by high hydration energy (ΔG_hyd_ = −1090 kJ mol^−1^) [9], which significantly hinders the formation of strong and durable host–sulfate complexes. In the presence of other anions such as chlorides (ΔG_hyd_ = −340 kJ mol^−1^) or nitrates (ΔG_hyd_ = −306 kJ mol^−1^) [9], which are more lipophilic, sulfate binding is unfavorable and follows the Hofmeister bias [10,11]. This also applies to the extraction of salts from the aqueous to the organic phase, where more lipophilic salts are extracted more effectively. Despite the high demand, the selective binding and removal of sulfates still poses serious problems and designing effective receptors capable of overcoming the Hofmeister series is of great interest. Three trends can be distinguished in research on the synthesis of such systems. A first approach uses macrocyclic anion receptors with a properly designed binding cavity for a tetrahedral sulfate anion [12,13,14]. The second approach involves designing acyclic receptors capable of forming stable complexes with sulfate anions through self-organization [15,16,17]. The last approach involves tripodal receptors (built of three connected arms), as a compromise between the macrocyclic and acyclic approaches. It is assumed that these compounds, by having flexible arms, can adapt to the geometry of the sulfate anion as in the case of macrocyclic receptors, which form strong, durable host–sulfate complexes [18]. Several platforms have been proposed for building tripodal receptors selective for sulfate anions, including a phenyl ring and its derivatives [19,20,21], cyanuric acid [22] and the most frequently used tris(2-aminoethyl)amine (TREN) [23,24]. By introducing specific binding sites, such as urea [25,26,27,28,29,30,31], thiourea [32] or a squaramide [33] function, to the specific platform, tripodal anion receptors capable of interacting with sulfate anions have been obtained. With the exception of two systems reported very recently [17,34] all sulfate receptors mentioned require the assistance of lipophilic counterions for their effective recognition or extraction. On the other hand, highly hydrated cations may compete with receptors for anion binding and hamper their action [35,36,37]. This great disadvantage may preclude the action of anion receptors in real-life scenarios where salts consisting of highly hydrated cations are present.

**Scheme 1:**
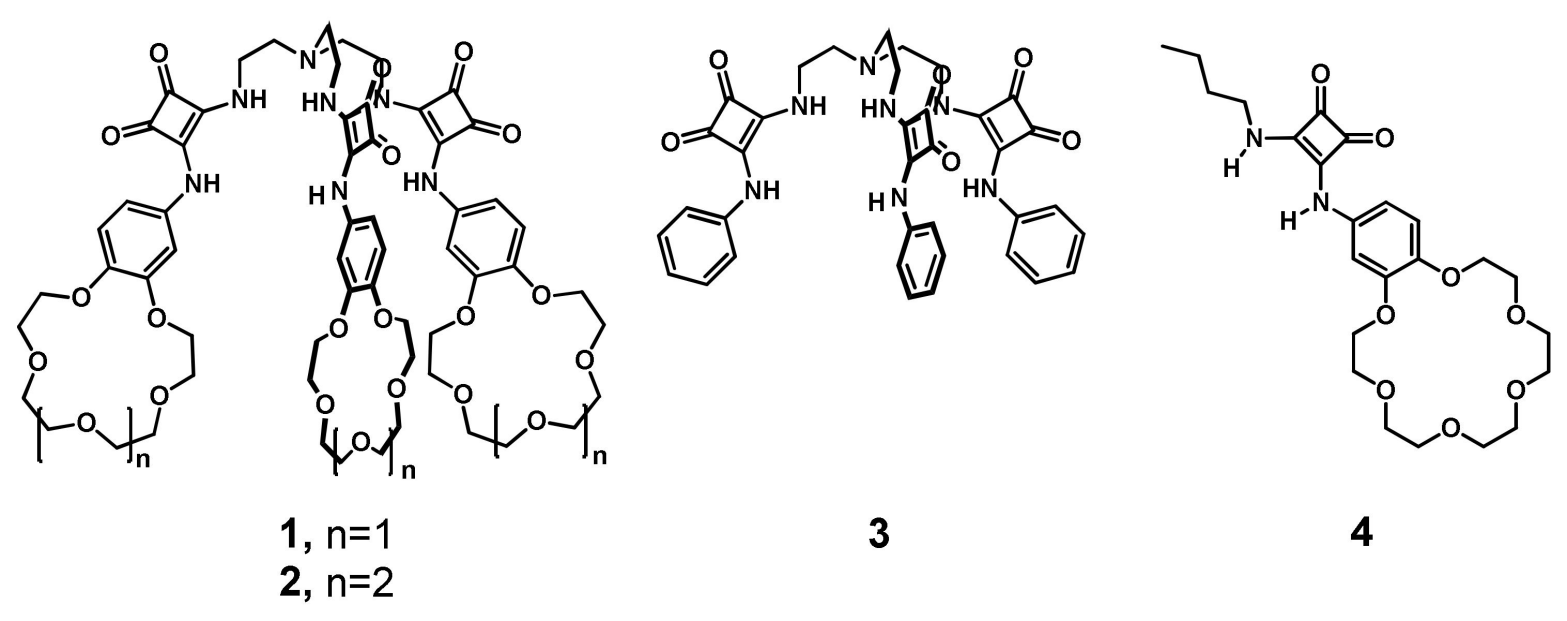
Structures of receptors **1**–**4**.

A solution to this problem may lie in the use of ion pair receptors able to interact with cations and anions simultaneously [38,39,40,41,42]. Surprisingly, the literature offers few reports of tripodal, TREN-based ion pair receptors and to the best of our knowledge this is limited to the tripodal amide designed by Beer and coworkers [43]. Given the superlative affinity of squaramides for anions [44,45,46,47] we envisioned that combining a squaramide unit with a cation binding domain and arranging it on the C-3 platform would make it possible to obtain highly selective and effective receptors capable of binding and extracting extremely hydrated anions (e.g., sulfates), even in the form of alkali metal salts. This assumption is based on the fact that tripodal receptors are prone to form supramolecular capsules with divalent anions, such a sulfates [48]. The dimeric (2:2) complexes thus formed would promote the extraction of sulfates rather than other monovalent salts, thus achieving the desired selectivity. To the best of our knowledge the literature serves only one example of a tripodal ion pair receptor able to extract sulfates in the form of alkali metal salts. This receptor is built on 1,3,5-tris(aminomethyl)-2,4,6-triethylbenzene scaffold [21]. However, to be closer to practical applications, a more accessible platform is needed, and the synthetic protocol should avoid chromatographic purification. By making use of the strategy of simultaneous binding by ion pair receptors of both cations and anions, we decided to expand this area on TREN-based ion pair receptors by also paying attention to the pH regime in which it can operate. In order to confirm the validity of the presented hypothesis, a tripodal, squaramide-based anion receptor **3** and an acyclic ion pair receptor **4**, mimicking a single arm of the tripodal salt receptor **2**, were also obtained and tested (Scheme 1).

## 2. Results

**Scheme 2:**
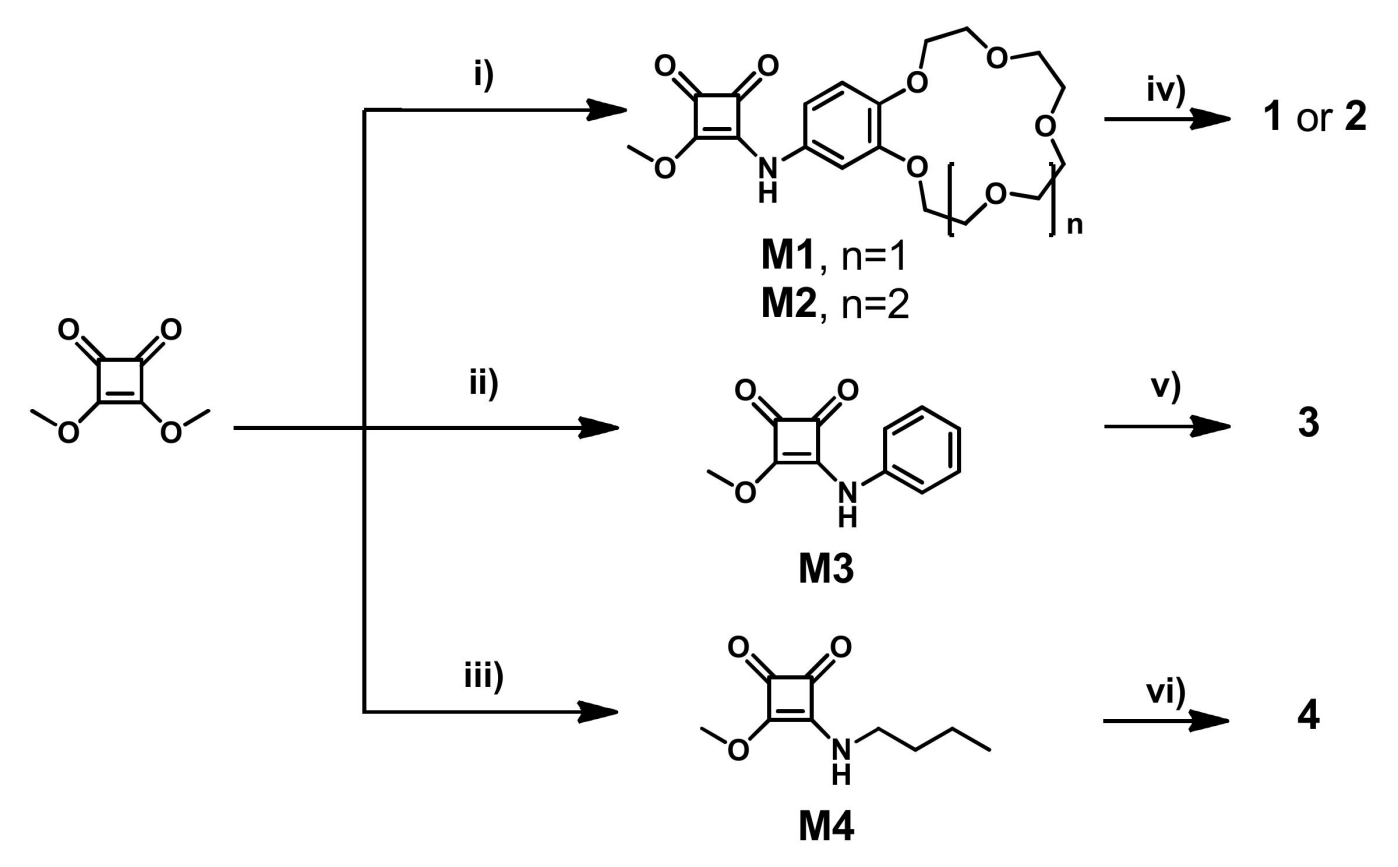
Synthesis of receptor **1–4**. Reagents and conditions: (i) methanol, 4-aminobenzo-15-crown ether or 4-aminobenzo-18-crown ether, 24 h, room temperature, 87% for **M1** and 86% for **M2**; (ii) aniline, methanol, 24 h, room temperature, 80%; (iii) methanol, *n-*butylamine, 24 h, room temperature, 85%; (iv) methanol, tris(2-aminoethyl)amine, 24 h, room temperature, 86% for **1** and 79% for **2**; (v) tris(2-aminoethyl)amine, methanol, 24 h, room temperature, 71%; (vi) 4-aminobenzo-18-crown-6 ether, methanol, 24 h, room temperature, 67%.

All the receptors can by synthesized in a simple, modular fashion by a two-step protocol using the stepwise amidation of dimethyl squarate and selected amines (Scheme 2). The first step produced monosquaramide modules **M1**–**M3** by reacting dimethyl squarate with 4-aminobenzo-15-crown-5 ether, 4-aminobenzo-18-crown-6 ether or aniline, respectively. A subsequent amidation of the obtained modules with tris(2-aminoethyl) amine (TREN) afforded tripodal ion pair receptor **1** and **2** with a 86% and 79% yield, respectively, and 71% for reference tripodal anion receptor **3**. Notably, all tripodal receptors were synthesized without using chromatographic purification. Ion pair receptor **4**, designed to mimic a single arm of tripodal receptor **2**, was obtained in similar way (84% yield) by the amidation of dimethyl squarate with *n*-butylamine and a second amidation with 4-amino-benzo-18-crown-6.

With receptors **1**–**4** in hand, we investigated their anion and ion pair binding ability using ^1^H NMR titration method in deuterated DMSO. Dilution experiments indicated that self-association does not occur in the investigated concentration range (2.3 × 10^−3^ to 3 × 10^−4^ M) (see Supplementary Materials). On the other hand, test experiments with tetrabutylammonium hexafluorophosphate or perchlorate, confirmed that receptors did not interact with TBA^+^, PF_6_^−^ or ClO_4_^−^ since addition of these salts to the solution of receptors in DMSO do not affect their spectra. Initially we selected interaction with chloride salt to screen the binding abilities of receptors **1**–**4** and to validate the design principles of the preorganization of anion and cation binding domains on each arm of the tripodal platform. For this purpose, titration with tetrabutylammonium chloride (TBACl) was used as an anion source, and in the case of sodium or potassium chloride recognition the assistance of sodium perchlorate or potassium hexafluorophosphate was assured (3 equiv. for receptors **1**–**3** and 1 equiv. for receptor **4**). The association constants, calculated by nonlinear regression analyses of the binding isotherms, are presented in Table 1.

We found that receptors **1**–**3** bind chloride anions moderately as a result of the simultaneous action of three anion binding sites located on each arm of the receptor. Their presence in receptor **1**, **2** and **4** caused the electron density enrichment of the aromatic ring, which led to the lower acidity of the amide protons. However, the obtained results showed that, in the case of the titration of receptors with in situ generated sodium or potassium salts (mixtures of TBACl and sodium perchlorate or potassium hexafluorophosphate), anion receptor **3** bound chloride less effectively than ion pair receptors **1** or **2**. This may be explained by the lack of a crown ether unit in the structure of receptor **3** and competitive ion pair formation out of the receptor. On the other hand, in the structure of receptor **1** or **2** the presence of cation-binding domains enhances anion binding when co-bound with cations. Of the investigated series, we found that the least effective receptor in chloride anion recognition was the “single-armed” ion pair receptor **4**, which interacted with chloride anions at less than a quarter of the strength of other tripodal receptors. Even the assistance of a sodium or potassium cation upon titration of **4** with a chloride anion could not drive enough enhancement in ion pair binding to reach at least as half of the value of the stability constants determined for ion pair receptors **1** or **2**. This clearly supports the idea of salt binding by tripodal receptors bearing heteroditopic binding sites on the C-3 platform, which enable the simultaneous action of a particular arm.

**Figure 1:**
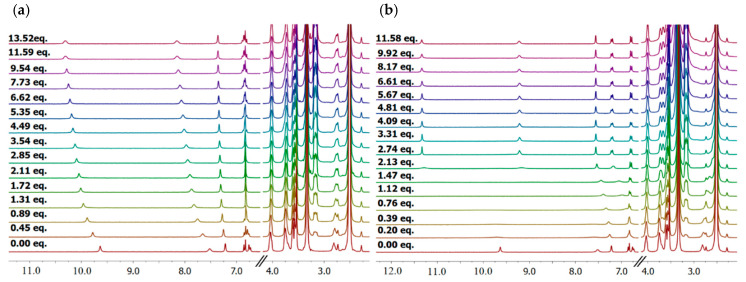
^1^H NMR titration of receptor **2** with TBACl (**a**) and TBA_2_SO_4_ (**b**) in DMSO-*d_6_*.

Likewise, receptor **2**, possessing a benzo-18-crown-6 ether unit as a cation binding site, was found to be the most effective in ion pair binding and the highest enhancement in chloride anion binding was achieved with the assistance of potassium cations. For this reason, an extension of the binding study was carried out for receptor **2** with selected anions and in situ generated potassium salts. The obtained association constants are listed in Table 2. We found that receptor **2** associated the examined anions moderately, in the following order: CH_3_COO^−^ > PhCOO^−^ > Cl^−^ > NO_2_^−^ > Br^−^. In the case of titration with NO_3_^−^ anions, excessively small chemical shifts made it impossible to determine the association constant, which suggests that, among the anions studied, receptor **2** created the weakest complexes with nitrates. In the case of SO_4_^2−^ anions, a different behavior was observed and the data obtained could not be fitted to an appropriate binding model (see Supplementary Materials), suggesting more complicated equilibria in solution. Upon the incremental addition of sulfates to the solution of **2** in deuterated DMSO the signals corresponding to squaramide protons were initially broadened and shifted downfield. After exceeding approx. two equivalents of added sulfates, both signals were sharpened and did not change their position to the end of titration, suggesting that equilibrium was reached (Figure 1b). The same trend was observed when tracking the signals corresponding to aromatic protons. Remarkably, while the position of ethylene protons upon titration of **2** with carboxylates, bromides, chlorides and nitrites were practically unchanged (Figure 1a), in the case of the addition of sulfates both of them were significantly shifted upfield. This can be explained by a different mechanism of action in the case of sulfates, which involves the spreading out of the receptor arms and adoption of a more planar trigonal conformation, or the contribution of a diamagnetic effect resulting from the close proximity of complexed sulfate anions to the ethylene groups [49]. This behavior matches well with the findings of Jin et al., who recently reported the binding properties of anion receptor **3**, which was used in the present study as a reference receptor. It was shown that **3** was able to trap two sulfate anions inside a pseudocapsule ensemble formed by two molecules of **3**, creating complexes with 2:2 (host:guest) stoichiometry [33]. Each of the two sulfate anions in such dimers is bounded by two squaramide functions of one molecule of receptor **3** and interact with a third arm, employing a second molecule of the receptor. Indeed, we found that all tripodal receptors **1**, **2** behave analogously to receptor **3** as reported by Jin et al. upon the addition of sulfate anions (see Supplementary Materials), suggesting that in equilibria the complexes with 2:2 stoichiometry (receptor **2**: sulfates) should also be taken into consideration. Interestingly, when the addition of sulfate anions to **2** exceeded approx. two equivalents, an additional signal appeared at approximately 9.63 ppm. We concluded that this signal came from a water molecule that may have taken part in the binding event. To verify this assumption, additional analyses were carried out in deuterated DMSO. The investigated signal revealed the exchange coupling with water molecule (ROESY), its correlation with any carbon was not observed (HSQC) and its diffusion coefficient (D = 0.90 × 10^−9^ m^2^ s^−1^) by DOSY was very close to the value of water in the system (D = 0.941 × 10^−9^ m^2^ s^−1^) and almost one magnitude of order higher than that of the receptor (D = 0.129 × 10^−9^ m^2^ s^−1^). Our finding is in agreement with the work reported by Jose et al., who demonstrated the participation of water molecules upon sulfate binding by a tripodal anion receptor consisting of nitrophenylurea functions arranged on a TREN platform [23]. It was found that two molecules of such a receptor formed a dimeric capsule in the solid state, catching two sulfate anions along with three molecules of water inside their cavity. These water molecules bridged the sulfate anions inside the cavity *via* multiple hydrogen-bonding interactions.

Notably, when receptor **2** was titrated in the presence of 3 equivalents of potassium cations, an enhancement in anion binding was observed in all binding events quantitatively established. The enhancement factor, defined as the quotient of the stability constant for complexes with anions in the presence of potassium cations to the stability constant of complexes with anions in their absence, reached the highest value of 1.95 for NO_2_^−^ recognition. Diffusion coefficients were also measured in deuterated DMSO for mixtures of receptor **2** with three equivalents of potassium cations together with one equivalent of chloride (D = 0.118 × 10^−9^ m^2^ s^−1^) or sulfate anions (D = 0.119 × 10^−9^ m^2^ s^−1^). As expected, upon the interaction of **2** with the tested potassium salts, the diffusion coefficient decreased as a consequence of the complexes formed. The diffusion coefficient value measured after the addition of potassium sulfate to **2** in DMSO, however, suggested that in equilibria the fraction of plausible 2:2 complexes is not dominant.

To confirm the aforementioned findings by an independent technique, UV–vis titration experiments were performed using receptors **1**–**4**, this time in less competitive media due to the estimated values of the stability constants. Based on titration in a CH_3_CN:DMSO 8:2 *(v/v)* mixture, analogous trends were found (Table 3). Specifically, anion receptor **3** bound chloride anions the most strongly, but in the presence of sodium or potassium cations significant decreases in the values of the association constants were noted. Again, significant enhancement in chloride binding was found when receptors **1**, **2** or **4** were titrated with this anion in the presence of cations, with **2** having a favorable affinity for potassium salt. Remarkably, the “single-armed” ion pair receptor **4** was definitely less effective and recognized potassium chloride with association constants two orders of magnitudes less than **2**. This confirms once again that the presence of three arms is necessary for the effective binding of anions and ion pairs. In CH_3_CN:DMSO 8:2 *(v/v)* mixture receptor **2** was also able to recognize nitrate anions with stability constants of K_TBANO3_ = 1.04 × 10^4^ M^−1^, a small enhancement in nitrate binding was found for *in situ* generated sodium nitrate (K_NaNO3_ = 1.11 × 10^4^ M^−1^) and complexes more than four times stronger were formed with the assistance of potassium cations (K_KNO3_ = 4.78 × 10^4^ M^−1^). Nevertheless, binding isotherms obtained after titration of **2** with sulfates showed inconsistency with a clear minimum for approx. 0.35 equivalents of anions added, signifying a multistep binding mechanism (see Supplementary Materials). This behavior made it impossible to fit the data to an appropriate binding model and determine a stability constant.

**Figure 2:**
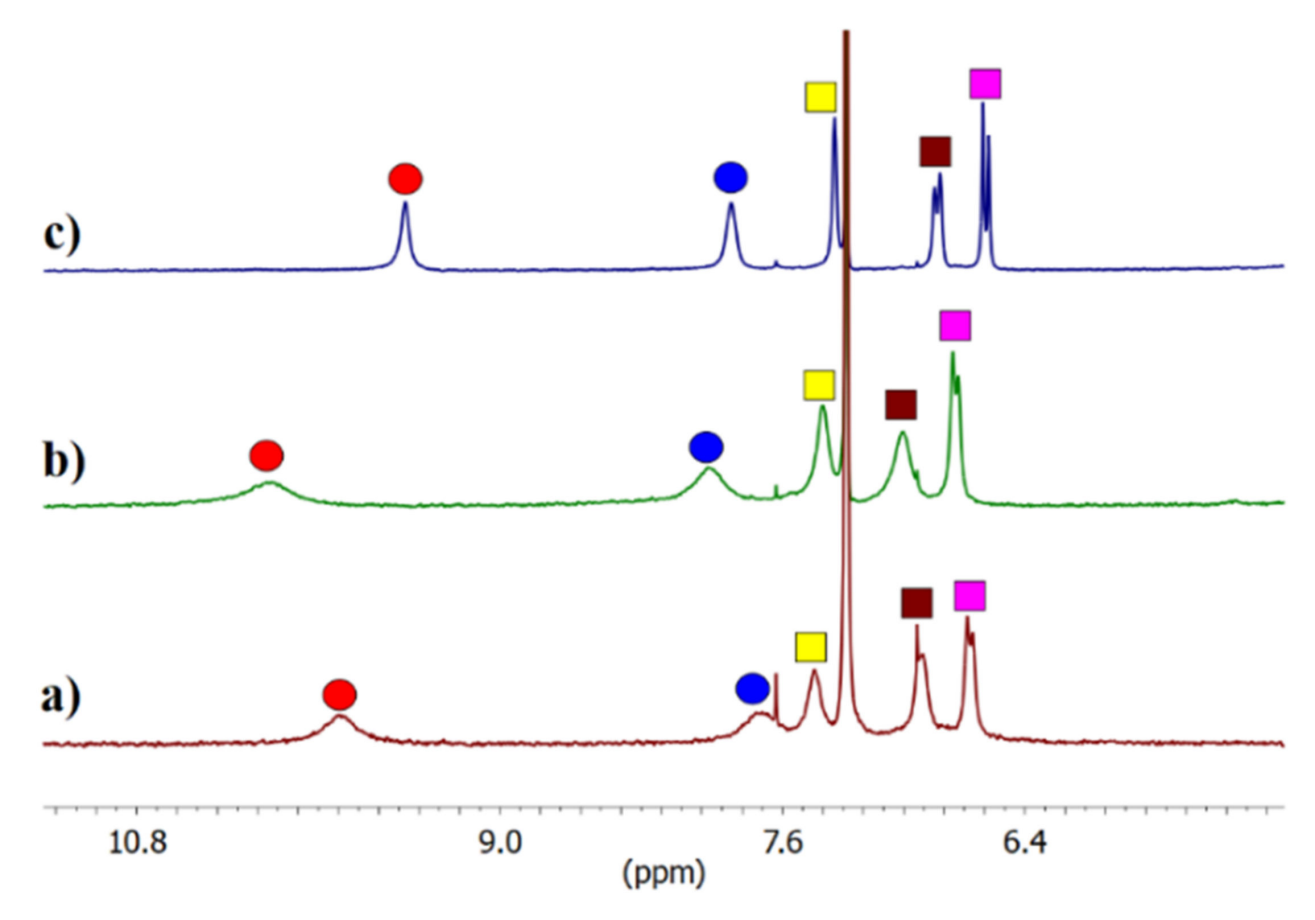
Partial ^1^H NMR spectra of receptor **2**: (**a**) 1.0 mM in wet CDCl_3_; (**b**) after extraction of 50 mM K_2_SO_4_ and (**c**) after extraction of 50 mM KCl.

We envisioned that thanks to the ability of **2** to form dimeric complexes with sulfates we would be able to selectively extract them in the presence of other salts which create 1:1 complexes. First, we found that initial suspension of **2** in CDCl_3_ was clarified after contact with water, suggesting that **2** forms an intermolecular interaction in this medium, which is partially broken in the presence of water. Evidence for complex formation in the organic phase came from LLE experiments carried out under ^1^H NMR control which showed that after the wet solution of **2** in CDCl_3_ (1 mM) came in contact with a 50 mM solution of K_2_SO_4_, the signals corresponding to squaramide protons were shifted downfield (Δδ 0.35 and 0.25 ppm); however, in both cases the signals were broadened (Figure 2). On the other hand, after LLE experiments carried out with aq. KCl, less pronounced shifts were found, this time together with a sharpening of the signals. The more pronounced changes in chemical shifts of NH signals upon extraction of aq. potassium sulfate rather than potassium chloride with **2** in chloroform may have indicated a higher extraction efficiency for the former. On the other hand, the difference in the signal width in wet chloroform after extraction suggested a different type of complex formed in organic phase.

**Figure 3:**
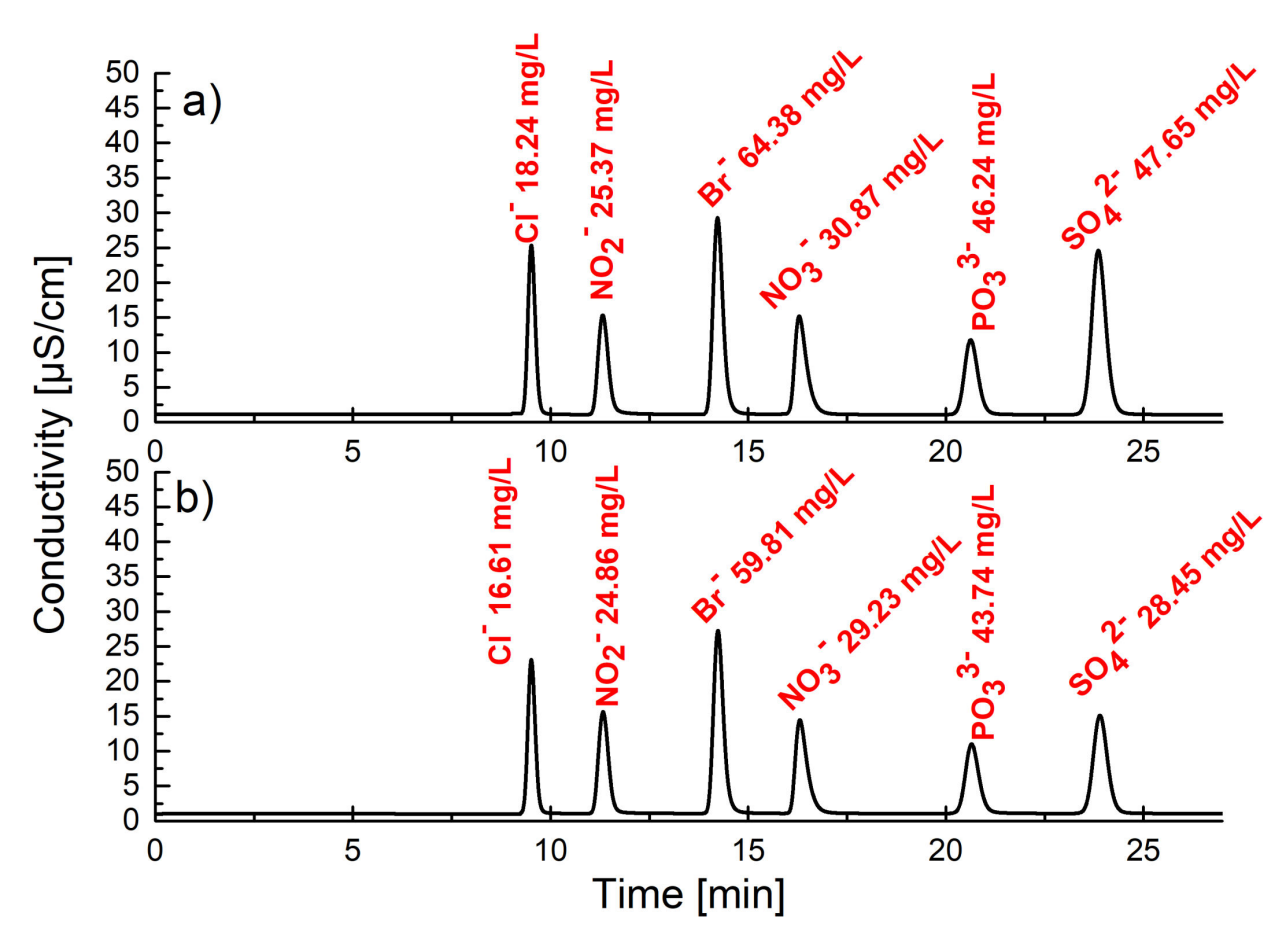
Chromatograms obtained during extraction experiment after tenfold dilution: (**a**) source phase; (**b**) after extraction with 5 mM **2** in CHCl_3_.

In order to quantify the extraction efficiency, LLE experiments were carried out under ion chromatography control. Specifically, aqueous solutions of KCl, KBr, KNO_2_, KNO_3_, K_2_SO_4_ or KH_2_PO_4_ (5 mM each) were extracted with a 5 mM suspension of **2** in chloroform, and the drop of anion concentration in the aqueous phase was measured (see Supplementary Materials). It was found that the highest drop in salt concentration was found for K_2_SO_4_ (52%), then for KCl (31%), KBr (28%), KNO_3_ (22%), KNO_2_ (19%) and KH_2_PO_4_ (9%). The recyclability of receptor **2** was confirmed by its role in a triple-cycle of extraction and a back extraction sequence carried out for aqueous potassium sulfate without loss of extraction efficiency. Interestingly, selectivity towards sulfates was even more pronounced when a competitive LLE experiment was carried out with a mixture of all potassium salts (5 mM each) (Figure 3). The corresponding drops in concentration for the particular salts were found to be: K_2_SO_4_ (40%), KCl (9%), KBr (7%), KNO_3_ (5%), KH_2_PO_4_ (5%) and KNO_2_ (2%). Electron spray ionization mass spectrometric measurements of the extracted organic solution showed only characteristic peaks (*m/z*) at 1397 [**2** + Cl^−^], 1442 [**2** + Br^−^] and 1496 [**2** + KSO_4_^−^], suggesting lower stability for the remaining complexes of **2** under measurements conditions. We found that receptor **2** was able to operate using an aqueous phase in the pH 3.5–8 range. Below and above this range no phase separation was noted upon extraction most likely due to the protonation of **2** below pH 3.5 and its deprotonation above pH 8. The change of pH of aqueous phase within the aforementioned range did not affect the extraction efficiency of potassium sulfate. However, when we carried out competitive extraction using an aqueous solution of all potassium salts (5 mM each) adjusted to pH 3.5 with HClO_4_, the drop of sulfate concentration was maintained and those for all other salts decreased. The corresponding drops in concentration for the particular salts were found to be: K_2_SO_4_ (41%), KCl (4%), KBr (3%), KNO_3_ (<1%), KH_2_PO_4_ (<1%) and KNO_2_ (<1%). This may be explained by the interaction of receptor **2** with the lipophilic perchlorate salt at the expense of other salts, excluding sulfates. Finally, binary mixtures consisting of five molar excesses of anions over sulfates were tested showing a preferential extraction for the latter salt (see Supplementary Materials). All strategies provided clearly supported the tendency of receptor **2** to overcome the Hofmeister bias and its ability to extract an extremely hydrophilic sulfate salt in the presence of lipophilic salts.

To shed light on the mechanism of action under interfacial conditions and verify the assumption of dimer formation, LLE experiments were tracked by ^1^H NMR DOSY experiments. Indeed, a very low diffusion coefficient was found (D = 0.36 × 10^−9^ m^2^ s^−1^) in a wet CDCl_3_ solution of **2**, much lower than expected for a monomer. We compared this value to the diffusion coefficient of the TMS molecule (D_TMS_ = 2.20 × 10^−9^ m^2^ s^−1^) measured as an internal standard in the same solution and performed a simple estimate based on the Stokes–Einstein theory of diffusion [50,51]. We found that the diffusion coefficient of **2** for a single molecule should be about 0.8–0.9 × 10^−9^ m^2^ s^−1^, and the mass of receptor **2** particles in the solution was several times greater than the mass of the monomer. Such a result suggested a strong associative state in the system. However, the experimental diffusion coefficient of **2** in wet CDCl_3_ increased after extraction with aq. K_2_SO_4_ (D = 0.40 × 10^−9^ m^2^ s^−1^), and even more when the aqueous solution of KCl was used (D = 0.51 × 10^−9^ m^2^ s^−1^).

**Figure 4:**
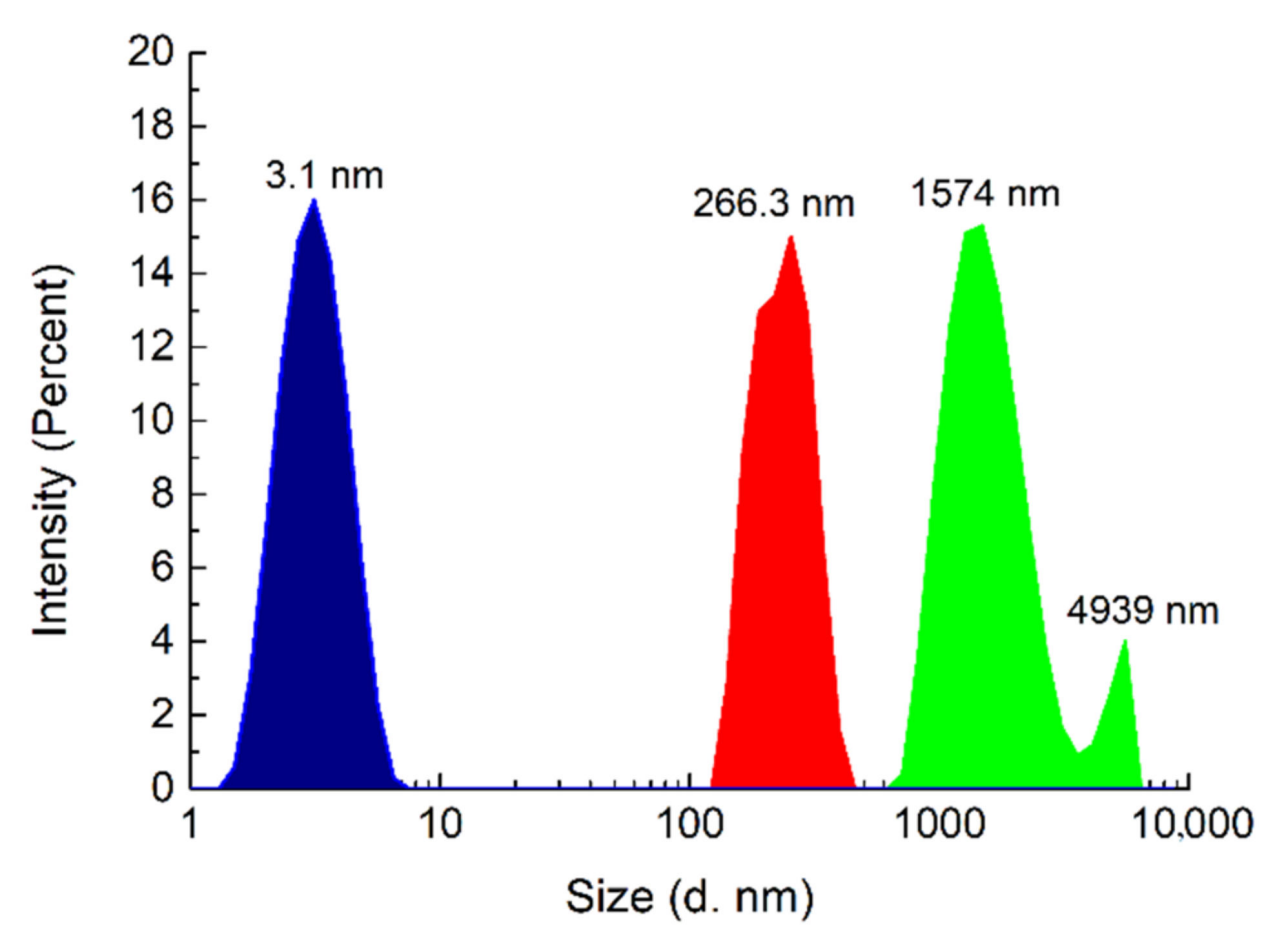
Distribution of the hydrodynamic diameter: 1 mM **2** in wet CHCl_3_ (green), after extraction of 50 mM K_2_SO_4_ (red), after extraction of 50 mM KCl (blue).

The collected data supports the conclusion that the initially formed intermolecular network in CDCl_3_ was subsequently destroyed and replaced by different associates upon contacting **2** with sulfate and chloride salts. We concluded that the main difference between the two ion-containing systems was the ability of tripodal receptor **2**, in the presence of SO_4_^2−^, to create several forms of complexes, including dimers (2:2 stoichiometry) coexisting with the initial network of receptor **2**. The basis for such structures is the assumption that only two arms of a given receptor **2** may be effectively involved in interacting with the sulfate tetrahedron, leaving the third arm free for other interactions, thus facilitating dimer formation. In contrast, the system with chloride ions was composed of the receptor **2** network and 1:1 complexes only. These data are also consistent with the DLS measurements, which showed that the highest solvodynamic radius was found for **2** in wet chloroform (1574 nm); a drop in the radius value was observed when **2** was extracted with aq. K_2_SO_4_ (266 nm), and a further decrease was noted after LLE with aq. KCl (3 nm) (Figure 4).

**Figure 5:**
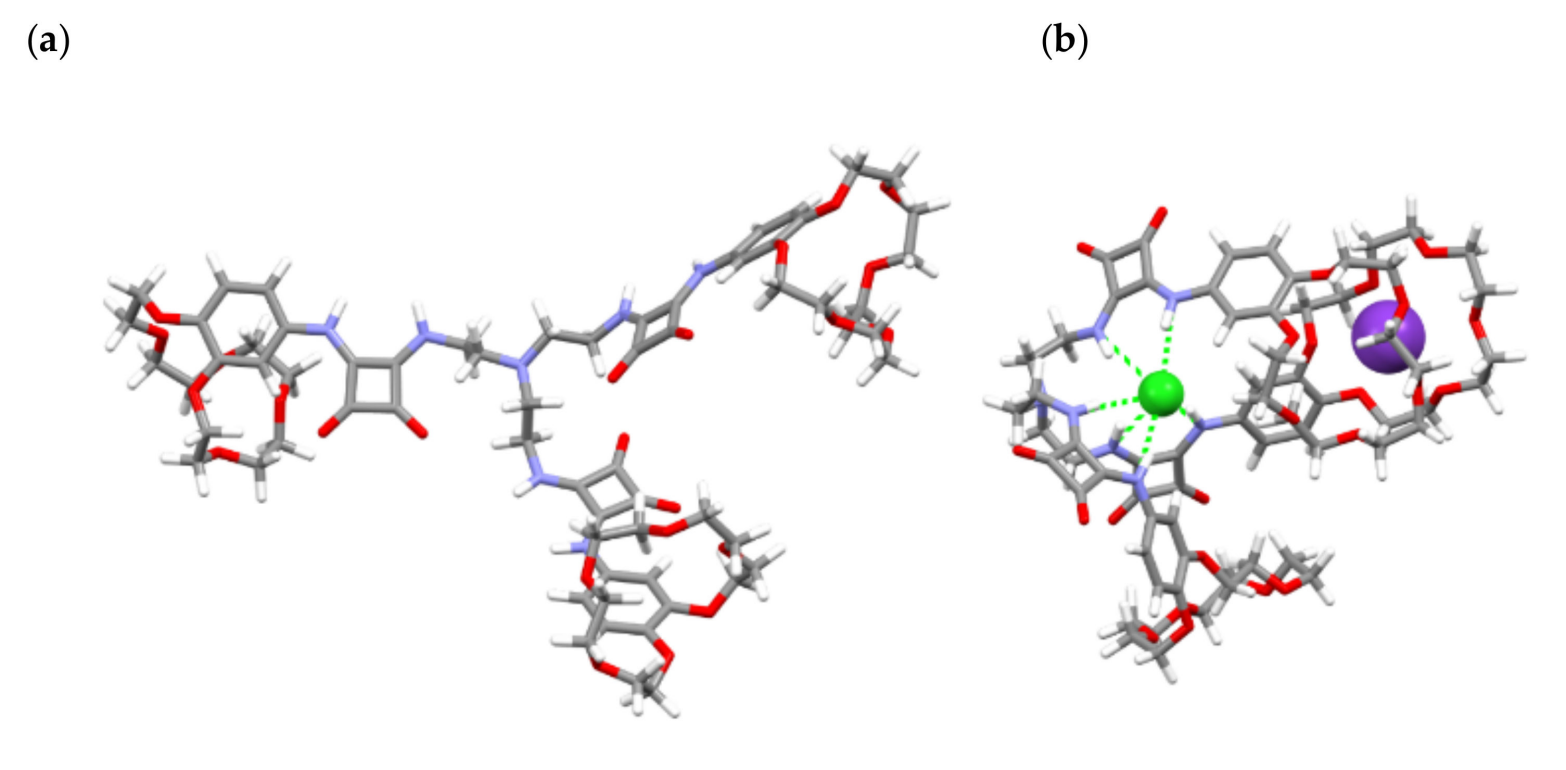
Minimized structures (DFT, B3LYP/6-31G*, Spartan’10, Wavefunction^®^) of receptor **2** (**a**) and complex of receptor **2** with KCl (**b**).

**Figure 6:**
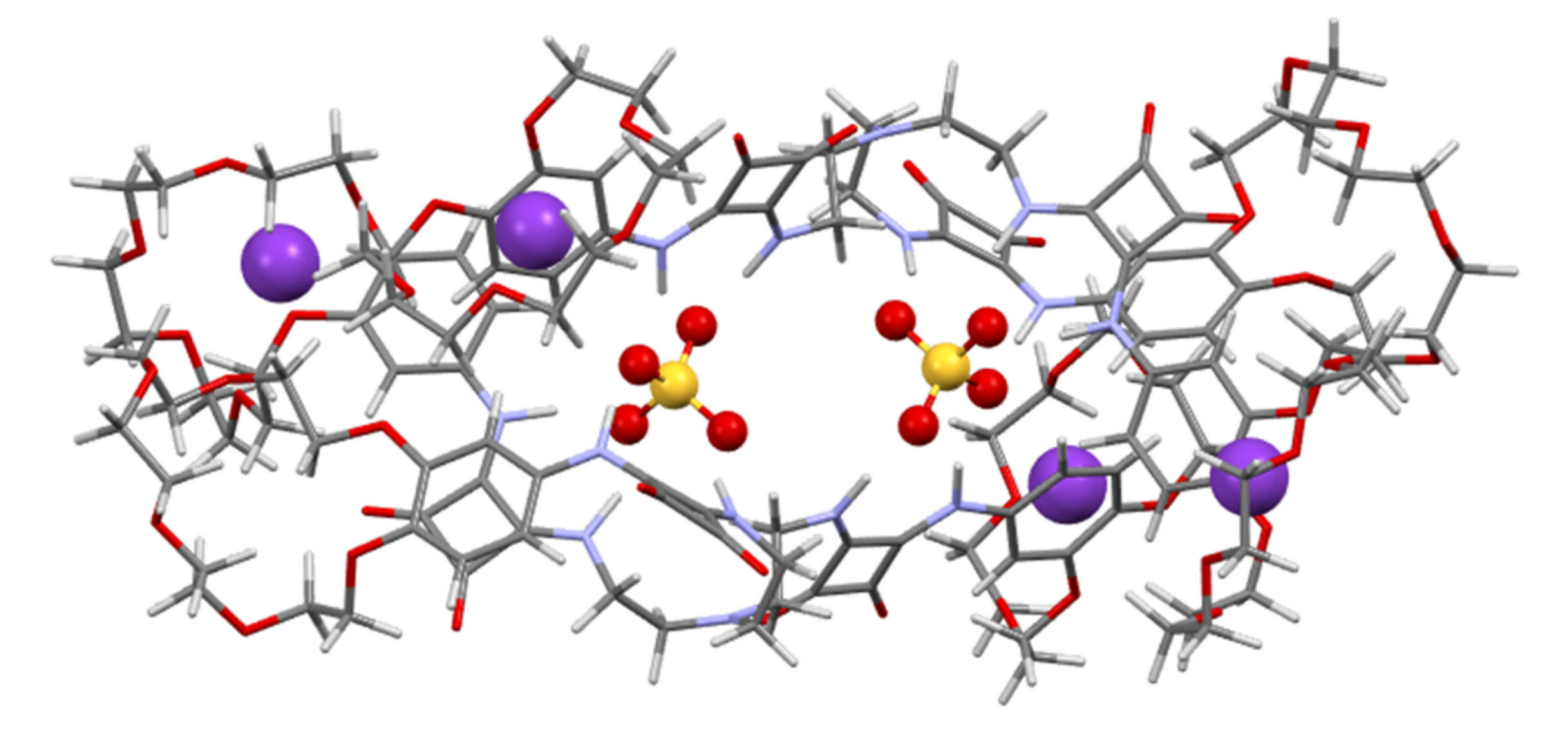
Minimized structures (Semi empirical PM6, Spartan’10, Wavefunction^®^) of receptor **2** and K_2_SO_4_.

Final proof that receptor **2** can interact with salts by utilizing the simultaneous action of three arms came from molecular modelling using Spartan 10 for Windows (Wavefunction, Inc. California, CA, USA, 2010). We performed calculations for receptor **2** and for its complexes with KCl and K_2_SO_4_ salts. Initially the structure of **2** was energy minimized using molecular mechanics followed by optimization by density functional theory (DFT) calculations at the B3LYP/6-31G* level of theory. In the case of the **2**⸦KCl complex, after MM energy minimization of structure **2**, a potassium cation was placed into one of the ether crown units and a chloride anion was located near one of the arm bearing anion binding sites and analogous DFT calculations were made. Based on the aforementioned results, which suggest the formation of a complex with a higher stoichiometry in the case of K_2_SO_4_, we carried out modelling with 2:2 stoichiometry. Potassium cations were placed inside the four crown ether cavities (two cations per one molecule of **2**) and two sulfate anions were located close to squaramide groups between two molecules of **2,** and the structure thus constructed was optimized by a semi-empirical method, specifically PM6. All structures obtained are presented in Figure 5 and Figure 6. Based on the calculations, we found that the receptor itself spread each of its arms out. This may have facilitated the intermolecular interaction and formation of supramolecular assemblies since a squaramide function possesses hydrogen bond donors and acceptors. On the other hand, in a KCl⸦**2** complex, a chloride anion is surrounded by the three receptor arms and bound by six squaramide hydrogen bonds. The H-bond length between Cl^−^ and the NH of the squaramide groups was calculated to be in the 3.29–3.54 Å range. The potassium cation resides on one of the ether crown units with K–O bond lengths in the 2.78–3.47 Å range, and the second crown ether unit located in the neighboring arm of the receptor was also affected by this cation, forming a sandwich-type structure. This may additionally force the preorganization of **2** and more effective anion binding by simultaneous action of the three arms. The modelled structure of (K_2_SO_4_)_2_⸦**2**_2_ complex showed that potassium cations occupy two out of three crown ether cavities in each of two ligands, with bond lengths between the crown ether oxygens in the range of 2.41–3.22 Å. The sulfate anion is complexed by two arms of one molecule of receptor **2** using an additional arm from a second receptor molecule, thus creating a head-to-tail-type dimer. The calculated H-bond length between a squaramide NH and sulfate oxygen ranged from 2.59 to 2.85 Å, suggesting a strong interaction. This corresponded well with the results obtained during NMR titration experiments, which showed that the highest signal shift for squaramide protons was observed upon the addition of sulfates. The calculated [K_2_SO_4_]_2_⸦**2**_2_ structure clearly resembles the crystallographic structure [(SO_4_)_2_]^4−^⸦**3**_2_ obtained by Jin et al. for a tripodal anion receptor, which is used in our study as reference compound **3** [33]. However, in the resulting [K_2_SO_4_]_2_⸦**2**_2_ structure, the two sulfate anions were strongly surrounded by the receptor arms of two ligands as well as by the simultaneous binding of potassium cations, which seems to be crucial for the selective and effective extraction of sulfate salts given that the receptor **3** lacking cation binding sites is unable to operate under interfacial conditions.

## 3. Materials and Methods

Unless specifically indicated, all chemicals and reagents used in this study were purchased from commercial sources and used as received. Solvents: acetonitrile (Sigma-Aldrich, Steinheim, Germany, 99.9%), chloroform (Avantor, Gliwice, Poland, 98.5%), methanol (Avantor, 99.8%), tetrahydrofuran (Avantor, 99.5%), dimethyl sulfoxide (Sigma-Aldrich, 99.7%), dimethyl sulfoxide-*d_6_* (Sigma-Aldrich, 99.9%), chloroform-*d* (Sigma-Aldrich, 99.8%). Reagents: benzo-15-crown-5 ether (Tokyo Chemical Industry, Tokyo, Japan, 97%), benzo-18-crown-6 ether (Tokyo Chemical Industry, 97%), aniline (Sigma-Aldrich, 99.5%), palladium on activated charcoal 10% Pd (Sigma-Aldrich), 3,4-dimethoxy-3-cyclobutene-1,2-dione (Sigma-Aldrich, 99%), 3,5-bis(trifluoromethyl)aniline (Sigma-Aldrich, 97%), tris(2-aminoethyl)amine (Sigma-Aldrich, 96%), *n*-butylamine (Sigma-Aldrich, 99.5%), triethylamine (Sigma-Aldrich, 99%). If the necessary purification of products were performed using column chromatography on silica gel (Merck Kieselgel 60, 230–400 mesh) with mixtures of chloroform/methanol. Thin-layer chromatography (TLC) was performed on silica gel plates (Merck Kieselgel 60 F254). ^1^H and ^13^C NMR spectra used in the characterization of products were recorded on BrukerAvance III HD 300 MHz spectrometer using a residual protonated solvent as internal standard. DOSY, ROESY and HSQC experiments were conducted at 298 K on Bruker Avance III HD 500 MHz instruments with a residual solvent signal as an internal standard. High-resolution mass spectra (HRMS) were measured on a Quattro LC Micromass unit using the ESI technique. UV–vis analyses were performed using Thermo Spectronic Unicam UV500 Spectrophotometer. Dynamic Light Scattering analyses were performed using a Malvern Zetasizer Nano ZS (Malvern Instruments Ltd., Malvern, UK) at 25 °C and a 173° angle relative to the source. The hydrodynamic diameter distributions were obtained by volume using the software package of the apparatus. Each curve represents the average of 3 measurements (16 runs each). Prior to analysis, all solutions were filtered and degassed. High-performance ion chromatography (HPIC) analyses were performed using a 930 Compact IC Flex apparatus (Metrohm AG, Herisau, Switzerland).

**Compound M1.** To a degassed solution of 4-nitrobenzo-15-crown-5 ether (1.10 g, 3.51 mmol) in 40 mL of a THF:MeOH mixture (1:4) 20 mg of 10% Pd/C was added. The reaction mixture was kept under a H_2_ atmosphere (balloon pressure) at room temperature overnight. The catalyst was removed by filtration through a pad of Celite and washed with MeOH. The filtrate was concentrated under reduced pressure to give the crude product in a near-quantitative yield (0.98 g). The obtained 4-aminobenzo-15-crown-5 ether was used in the next step without further purification.

To the solution of 4-aminobenzo-18-crown-6 ether (0.98 g 3.46 mmol) in methanol (20 mL), 3,4-dimethoxy-3-cyclobutene-1,2-dione (0.49 g, 3.45 mmol) was added. The reaction mixture was stirred at room temperature overnight. The resulting precipitate was isolated by filtration and obtained solid material was washed several times with methanol. The obtained white solid was dried in vacuo to give the desired product (1.18 g, 3.00 mmol, 87% yield).

HRMS (ESI): calcd for C_13_H_7_F_6_NO_3_Na [M + Na]^+^: 416.1321, found: 416.1333. ^1^H NMR (300 MHz, DMSO-*d*_6_) δ 10.64 (s, 1H), 7.17–6.95 (m, 1H), 6.95–6.90 (m, 1H), 6.87–6.77 (m, 1H) 4.37 (s, 3H), 4.07–3.99 (m, 4H), 3.82–3.72 (m, 4H), 3.65–3.58 (m, 8H). ^13^C NMR (75 MHz, DMSO-*d*_6_) δ 189.1, 184.1, 178.7, 169.6, 149.3, 145.9, 132.1, 114.9, 112.23, 106.9, 70.9, 70.9, 70.3, 70.2, 69.4, 69.4, 69.1, 68.7, 60.9.

**Receptor 1.** To the solution of compound M1 (0.40 g, 1.02 mmol) in MeOH (10 mL), tris(2-aminoethyl)amine (0.044 g, 0.3 mmol) was added and the mixture was stirred overnight at room temperature. The precipitate was isolated by centrifuge and washed several times by MeOH and diethyl ether. The collected solid was recrystallized by slow vapor–vapor diffusion of methanol to DMSO solution containing **1**. The obtained light-yellow solid was dried in vacuo to give the desired product (0.32 g, 0.26 mmol, 86% yield).

HRMS (ESI): calcd for C_60_H_75_N_7_O_21_Na [M + Na]^+^: 1252.4913, found: 1252.4883. ^1^H NMR (300 MHz, DMSO-*d_6_*) δ 9.64 (s, 1H), 7.53 (s, 1H), 7.19 (s, 1H), 6.91–6.69 (m, 2H), 4.11–3.92 (m, 4H), 3.87–3.54 (m, 14H), 2.9–2.75 (m, 2H). ^13^C NMR (75 MHz, DMSO-*d*_6_) δ 184.1, 180.6, 169.1, 164.2, 149.7, 144.7, 133.4, 115.4, 110.5, 105.3, 70.9, 70.9, 70.4, 70.1, 69.6, 69.5, 69.2, 68.5, 54.8, 42.3.

**Compound M2.** To a degassed solution of 4-nitrobenzo-18-crown-6 ether (1.5 g, 4.20 mmol) in 40 mL of a THF/MeOH mixture (1:4) 20 mg of 10% Pd/C was added. The reaction mixture was kept under an H_2_ atmosphere (balloon pressure) at room temperature overnight. The catalyst was removed by filtration through a pad of Celite and washed with MeOH. The filtrate was concentrated under reduced pressure to give the crude product in near quantitative yield (1.35 g). The obtained 4-aminobenzo-15-crown-5 ether was used in the next step without further purification. To the solution of 4-aminobenzo-18-crown-6 ether (1.35 g 4.12 mmol) in methanol (20 mL), 3,4-dimethoxy-3-cyclobutene-1,2-dione (0.59 g, 4.15 mmol) was added. The reaction mixture was stirred at room temperature overnight. The resulting precipitate was isolated by filtration and the obtained solid material was washed several times with methanol. The obtained white solid was dried in vacuo to give the desired product (1.48 g, 3.38 mmol, 82% yield).

HRMS (ESI): calcd for C_21_H_27_NO_9_Na [M + Na]^+^: 460.1584, found: 460.1591. ^1^H NMR (300 MHz, DMSO-*d*_6_) δ 10.64 (s, 1H), 7.15–6.95 (m, 1H), 6.94–6.86 (m, 1H), 6.84–6.74 (m, 1H) 4.37 (s, 3H), 4.07–3.99 (m, 4H), 3.83–3.72 (m, 4H), 3.65–3.58 (m, 8H). ^13^C NMR (75 MHz, DMSO-*d*_6_) δ 188.7, 183.8, 178.5, 169.1, 148.8, 145.5, 131.9, 113.9, 112.1, 106.3, 70.4, 70.3, 70.3, 69.2, 69.1, 68.9, 68.5.

**Receptor 2.** To the solution of compound **M2** (0.63 g, 1.44 mmol) in MeOH (10 mL), tris(2-aminoethyl)amine (0.058 g, 0.39 mmol) was added and the mixture was stirred overnight at room temperature. The precipitate was isolated by centrifuge and washed several times by MeOH and diethyl ether. The collected solid was recrystallized by slow vapor-vapor diffusion of methanol to DMSO solution containing **2**. The obtained white solid was dried in vacuo to give the desired product (0.42 g, 0.31 mmol, 79% yield).

HRMS (ESI): calcd for C_66_H_87_N_7_O_24_Na [M + Na]^+^: 1384.5701, found: 1384.5713. ^1^H NMR (300 MHz, DMSO-*d*_6_) δ 9.64 (s, 1H), 7.53 (s, 1H), 7.22 (s, 1H), 6.8–6.69 (m, 2H), 4.01–3.83 (m, 4H), 3.80–3.44 (m, 18H), 2.9–2.76 (m, 2H). ^13^C NMR (75 MHz, DMSO-*d*_6_) δ 184.0, 180.7, 169.1, 164.2, 149.2, 144.5, 133.1, 114.4, 110.3, 105.0, 70.4, 70.3, 69.3, 69.1, 69.0, 68.4, 54.8, 42.3.

**Compound M3.** To a solution of 3,4-dimethoxy-3-cyclobutane-1,2-dione (0.76 g, 5.36 mmol) in MeOH (20 mL), aniline (0.49 mL, 5.36 mmol) was added at room temperature. After stirring for 1 day the reaction mixture was filtrated and the collected solid material was washed with MeOH and diethyl ether. The obtained light-white solid was dried in vacuo to give the desired product S3 (0.87 g, 4.28 mmol, 80%).

HRMS (ESI): calcd for C_11_H_9_NO_3_Na [M + Na]^+^: 226.0485, found: 226.0479. ^1^H NMR (300 MHz, DMSO-*d*_6_) δ 10.76 (s, 1H), 7.43–7.31 (m, 4H), 7.18–7.07 (m, 1H), 4.39 (s, 3H). ^13^C NMR (75 MHz, DMSO-*d*_6_) δ 188.5, 184.4, 179.1, 169.6, 138.4, 129.5, 124.5, 120.0, 61.0.

**Receptor 3.** To a solution of compound M3 (400 mg, 4.97 mmol) in MeOH (10 mL) was added aniline (56 mg, 0.60 mmol) at room temperature. The mixture was stirred for 2 days. Then the reaction mixture was filtrated and the collected solid material was washed with MeOH. Obtained white solid was dried in vacuo to give the desired receptor (170 mg, 0.425 mmol, 71%).

HRMS (ESI): calcd for C_36_H_33_N_7_O_6_Na [M + Na]^+^: 682.2395, found: 682.2390. ^1^H NMR (300 MHz, DMSO-*d*_6_) δ 9.73 (s, 1H), 7.63 (s, 1H), 7.47–7.36 (m, 2H), 7.35–7.23 (m, 2H), 7.05–6.95 (m, 1H), 3.85–3.62 (m, 2H), 2.93–2.75 (m, 2H). ^13^C NMR (75 MHz, DMSO-*d*_6_) δ 184.7, 180.5, 169.5, 164.3, 139.4, 129.7, 123.1, 118.6, 54.7, 42.2.

**Compound M4.** To a solution of 3,4-dimethoxy-3-cyclobutane-1,2-dione (300 mg, 2.11 mmol) and TEA (0.32 mL, 2.3 mmol, 1.1 eq.) in MeOH (10 mL), *n*-butylamine (0.2 mL, 2.1 mmol) was added at room temperature. After stirring for 24 h the reaction mixture was concentrated under reduced pressure and the residue was separated by silica gel column chromatography using mixture of MeOH in CHCl_3_ (2:98 *v/v*). Thee obtained solid was dried in vacuo to give the desired product M4 (330 mg, 1.8 mmol, 85%), which at 298 K exists in DMSO-*d*_6_ solution in the form of two conformers [46].

HRMS (ESI): calcd for C_9_H_13_NO_3_Na [M + Na]^+^: 206.0798, found: 206.0791. ^1^H NMR (300 MHz, DMSO-*d*_6_, 298 K) δ 8.80 (s, 0.55H), 8.58 (s, 0.45H), 4.35–4.22 (m, 3H), 3.55–3.35 (m, 0.9H), 3.30–3.20 (m, 1.1H), 1.57–1.42 (m, 2H), 1.37–1.21 (m, 2H), 0.94–0.81 (m, 3H). ^1^H NMR (300 MHz, DMSO-*d*_6_, 333 K) δ 8.75–8.35 (s, 1H), 4.30 (s, 3H), 3.61–3.28 (m, 2H), 1.58–1.46 (m, 2H), 1.39–1.25 (m, 2H), 0.94–0.86 (m, 3H). ^13^C NMR (75 MHz, CDCl_3_) δ 189.8, 182.7, 177.6, 172.2, 60.5, 44.7, 32.5, 19.5, 13.6.

**Receptor 4.** To a solution of compound **M4** (163 mg, 0.89 mmol) in MeOH (10 mL) was added 4-aminobenzo-18-crown-6 ether (280 mg, 0.85 mmol) at room temperature. The mixture was stirred for 24 h. Then the reaction mixture was filtrated and the collected solid material was washed with MeOH. The obtained white solid was dried in vacuo to give the desired receptor (275 mg, 0.57 mmol, 67%).

HRMS (ESI): calcd for C_24_H_34_N_2_O_8_Na [M + Na]^+^: 501.2213, found: 501.2220. ^1^H NMR (300 MHz, DMSO-*d*_6_) δ 9.51 (s, 1H), 7.54 (s, 1H), 7.27 (s, 1H), 6.96–6.85 (m, 1H), 6.83–6.72 (m, 1H), 4.17–3.97 (m, 4H), 3.84–3.46 (m, 18H), 1.63–1.46 (m, 2H), 1.45–1.27 (m, 2H), 1.01–0.85 (m, 3H). ^13^C NMR (75 MHz, DMSO-*d*_6_) δ 183.6, 180.7, 169.4, 163.9, 149.1, 144.2, 133.4, 114.2, 110.1, 104.7, 70.2, 69.2, 69.0, 68.7, 68.2, 43.8, 33.1, 19.5, 14.0.

## 4. Conclusions

In summary, we synthesized and characterized the family of receptors **1–4** and proved the better binding properties of **2**, which uses the feature of tripodal arrangement of multiple, heteroditopic binding sites. By ^1^H NMR and DLS measurements we showed that the initially formed intermolecular network of **2** in chloroform was affected upon contact with ions and formed new inorganic–organic associates soluble in organic phase. The different mechanism of action for **1** and sulfates rather than for other monovalent anions opened up a way to overcame Hofmeister series and favored selective extraction of sulfates in the presence of more lipophilic salts. The lack of key elements in the tripodal anion receptor **3** or the single-armed ion pair receptor **4** resulted in a weaker interaction with ion pairs and precluded operation under interfacial conditions.

## Figures and Tables

**Table 1:** Association constants (*K_a_*) for interactions between receptors **1**–**4** and chloride anions and apparent association constants for interaction of receptors **1**–**4** with chloride in the presence of three equivalents of sodium perchlorate or potassium hexafluorophosphate ^a^.

	TBACl	TBACl + 3 equiv. of NaClO_4_	TBACl + 3 equiv. of KPF_6_	KNa/KTBA	KK/KTBA
**1**	643 ± 19	651 ± 20	- ^b^	1.01	-
**2**	647 ± 22	694 ± 20	808 ± 17	1.07	1.25
**3**	695 ± 33	566 ± 21	438 ± 16	0.81	0.63
**4**	137 ± 1	159 ± 1 ^c^	245 ± 3 ^c^	1.16	1.79

^a 1^H NMR, solvent DMSO-*d_6_*, 298 K, [**1**] = 1.56 × 10^−3^ M, [**2**] = 1.28 × 10^−3^ M, [**3**] = 1.34 × 10^−3^ M, [**4**] = 1.14 × 10^−3^ M, chloride added as TBACl, [TBACl] ~0.02 M; M^−1^, 1:1 binding model used. ^b^ The obtained data could not be fitted to an appropriate binding model. ^c^ one equivalent of sodium perchlorate or potassium hexafluorophosphate was added.

**Table 2:** Association constants (*K*_a_) for interactions between receptor **2** and selected anions and apparent association constants for interaction of receptor **2** with anions in the presence of three equivalent of potassium hexafluorophosphate ^a^.

	2	2 + 3 equiv. of KPF_6_	K_K_/K_TBA_
**Cl^−^**	647 ± 22	808 ± 17	1.25
**Br^−^**	70 ± 3	117 ± 3	1.67
**NO_2_^−^**	81 ± 2	158 ± 5	1.95
**NO_3_^−^**	**-** ^b^	-^b^	-
**CH_3_COO^−^**	980 ± 61	987 ± 68	1.00
**PhCOO^−^**	782 ± 20	860 ± 35	1.10
**SO_4_^2−^**	- ^c^	-^c^	-

^a 1^H NMR, solvent DMSO-*d*_6_, 298 K, [**2**] = 1.2 × 10^−3^ M, anions added as TBA salts, [TBAX] ~0.02 M; M^−1^, 1:1 binding model used. ^b^ Binding too weak to obtain an accurate *K_a_*. ^c^ The data obtained could not be fitted to a 1:1 or 2:1 binding model.

**Table 3:** Association constants (*K*_a_) for interactions between receptors **1**–**4** and chloride anions and apparent association constants for interaction of receptors **1**–**4** with chloride in the presence of three equivalents of sodium perchlorate or potassium hexafluorophosphate ^a^.

	TBACl	TBACl + 3 equiv. of NaClO_4_	TBACl + 3 equiv. of KPF_6_	K_Na_/K_TBA_	K_K_/K_TBA_
**1**	1.3 × 10^5^	1.5 × 10^5^	-	1.9	-
**2**	1.3 × 10^5^	1.6 × 10^5^	3.0 × 10^5^	1.2	2.2
**3**	2.1 × 10^5^	1.6 × 10^5^	1.2 × 10^5^	0.8	0.6
**4 ^b^**	8.2 × 10^2^	9.2 × 10^2^	1.1 × 10^3^	1.1	1.3

^a^ UV–Vis, solvent CH_3_CN:DMSO (8:2, *v:v*), 298 K, [**1**] = 8.46 × 10^−6^ M, [**2**] = 7.65 × 10^−6^ M, [**3**] = 8.47 × 10^−6^ M, [**4**] = 2.98 × 10^−5^ M, chloride added as TBACl salt, [TBACl] ~0.4 mM; M^−1^, errors < 10% see SI. ^b^ one equiv. of NaClO_4_ or KPF_6_ was used.

## Data Availability

All data generated or analysed during this study are included in this published article and its Supplementary Materials file.

## Supplementary Materials

The following are available online.

## Sample Availability

Samples of the compounds are not available from the authors.
