# Supplementary material for: Tripodal, Squaramide-Based Ion Pair Receptor for Effective Extraction of Sulfate Salt
Source: Molecules. 2021 May 7;26(9):2751. doi: 10.3390/molecules26092751 (PMC8125518; doi:10.3390/molecules26092751)
Supplement: Supplementary file 1 [file molecules-26-02751-s001.zip › molecules-1210127-supplementary.pdf]

Tripodal, squaramide-based ion pair receptor for effective and selective extraction of sulfate salt.

by

Damian Jagleniec, Marcin Wilczek and Jan Romański*

Faculty of Chemistry, University of Warsaw, Pasteura 1, PL 02-093 Warsaw, Poland

*jarom@chem.uw.edu.pl

TABLE OF CONTENTS

1. UV-vis measurements (Binding isotherms) 1

2. NMR measurements (Binding isotherms) 10

3. Extraction experiments 33

4. NMR DOSY, ROESY, HSQC 42

5. Molecular Modelling 47

6. NMR spectra 63

UV-vis titration experiments

The UV-Vis titration was performed using Thermo Spectronic Unicam UV500 Spectrophotometer at 298K in acetonitrile/dimethyl sulfoxide mixture (8:2 v/v). In each case, a 2500 μL of freshly prepared 9.05×10^{-6} M solution of receptor was added to a cuvette and small aliquots of TBAX, containing constant concentration of the receptor, were added and a spectrum was acquired after each addition. In the case of ion pair titration receptor **1, 2** and **3** was firstly pretreated with three equivalent of KPF_6 or $NaClO_4$ (refers to receptor). In the event of ion pair titration receptor **4** was firstly pretreated with one equivalent of KPF_6 or $NaClO_4$. The resulting titration data were analyzed using BindFit (v0.5) package, available online at http://supramolecular.org

Fig. S1. UV-Vis titration of receptor **1** with TBACl and selecting binding isothermes.

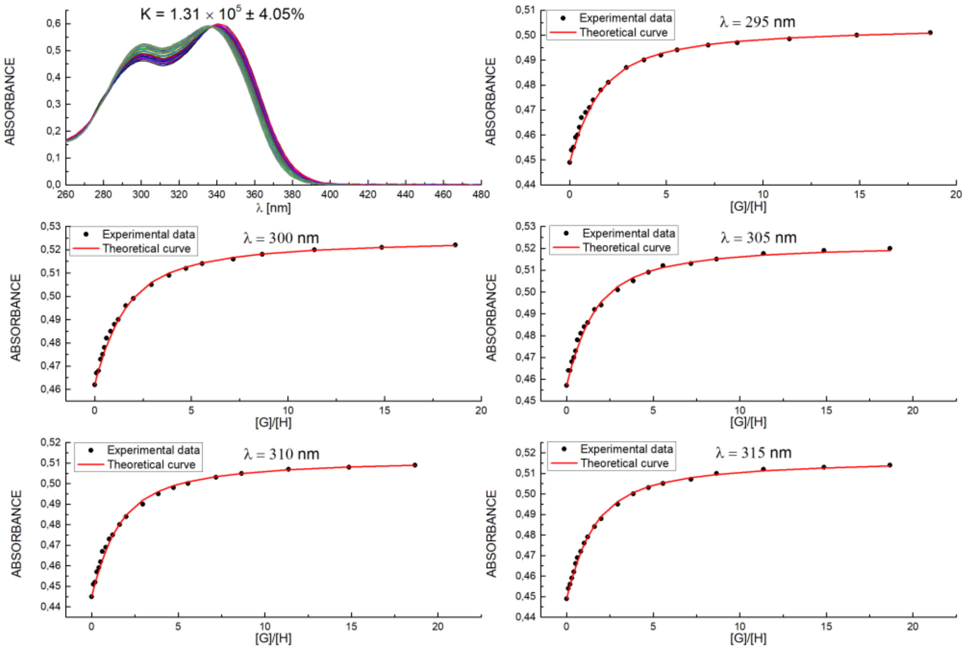

Fig. S2. UV-Vis titration of receptor **1** with TBACl and in the presence of 3 equivalents of $NaClO_4$ and selecting binding isothermes.

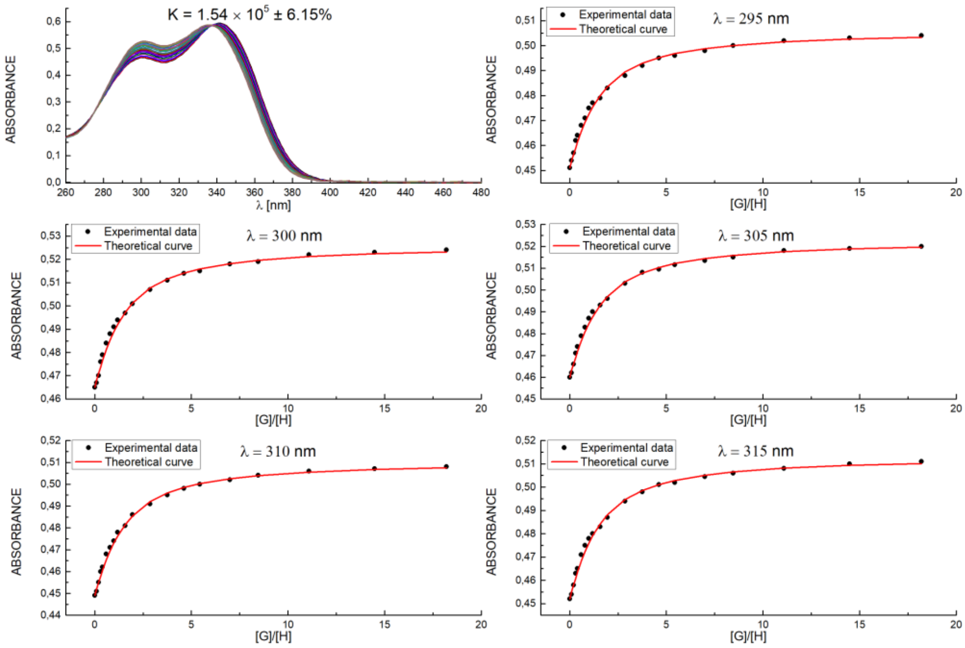

Fig. S3. UV-Vis titration of receptor **2** with TBACl and selecting binding isothermes.

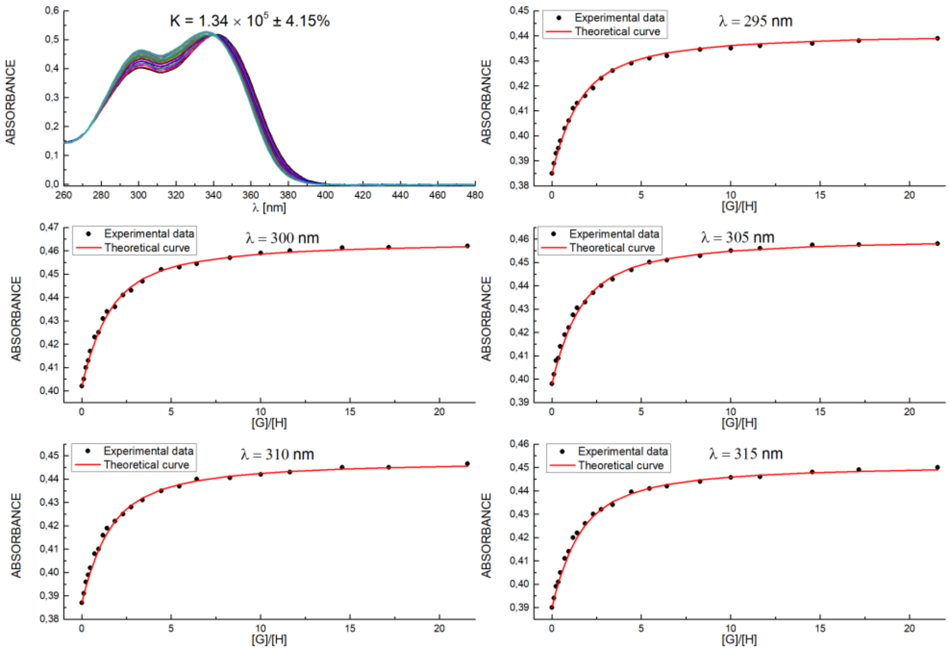

Fig. S4. UV-Vis titration of receptor **2** with TBACl in the presence of 3 equivalents of $NaClO_4$ and selecting binding isothermes.

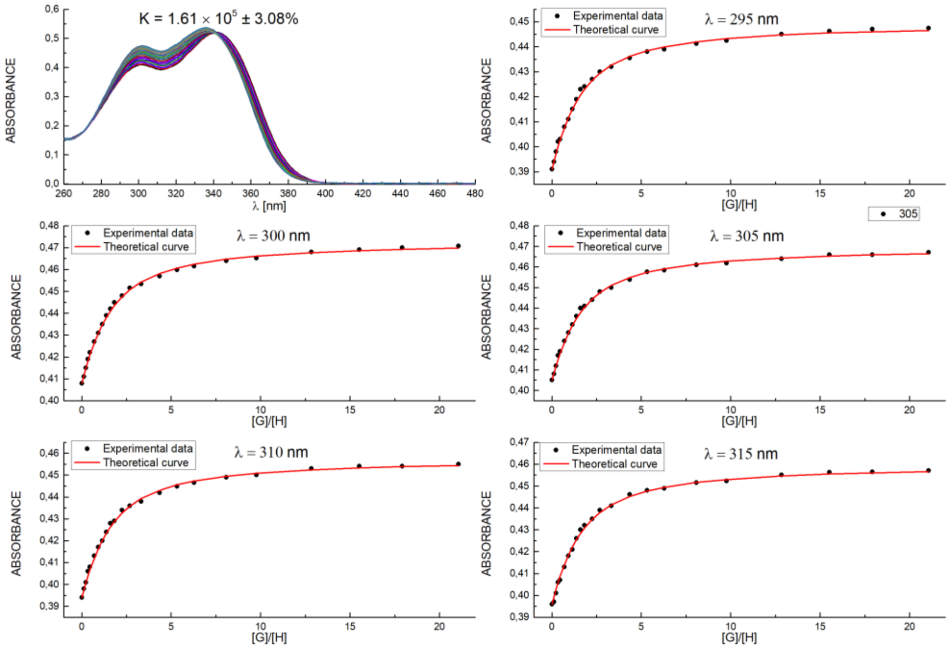

Fig. S5. UV-Vis titration of receptor **2** with TBACl in the presence of 3 equivalents of KPF_6 and selecting binding isothermes.

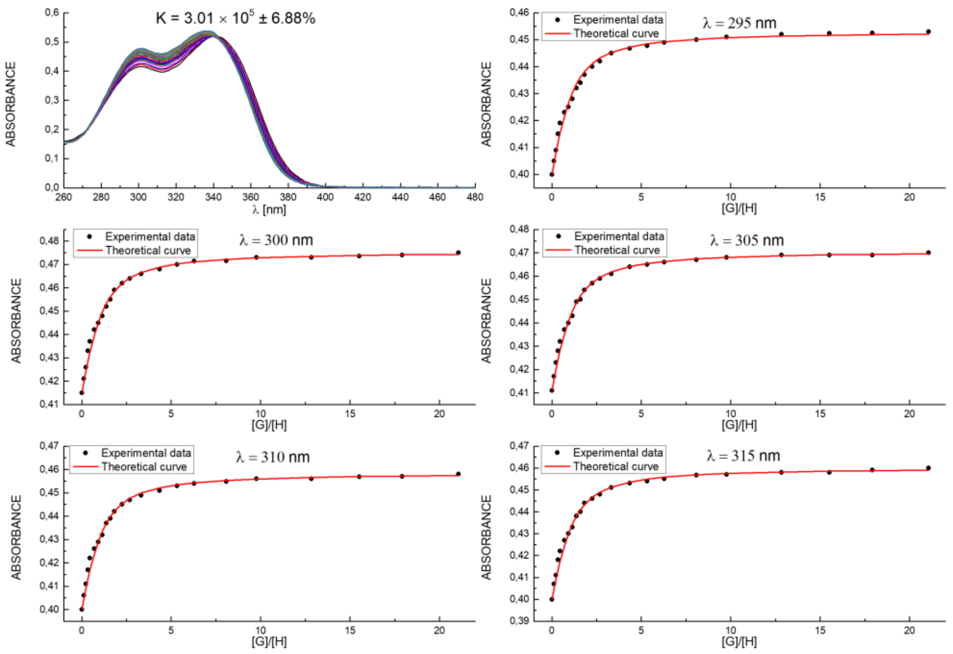

Fig. S6. UV-Vis titration of receptor **2** with $TBANO_3$ and selecting binding isothermes.

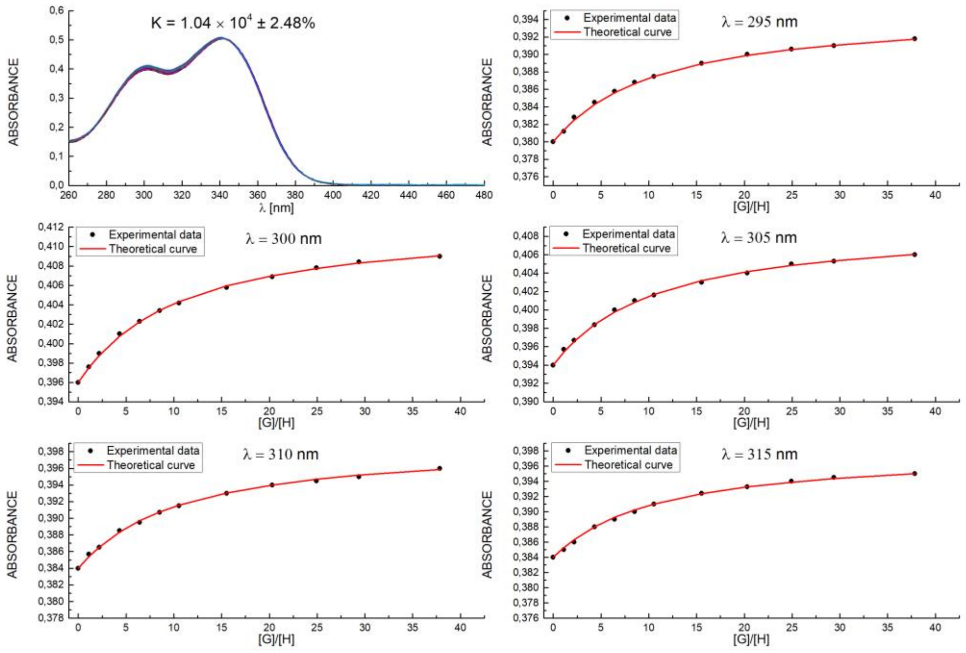

Fig. S7. UV-Vis titration of receptor **2** with $TBANO_3$ in the presence of 3 equivalents of $NaClO_4$ and selecting binding isothermes.

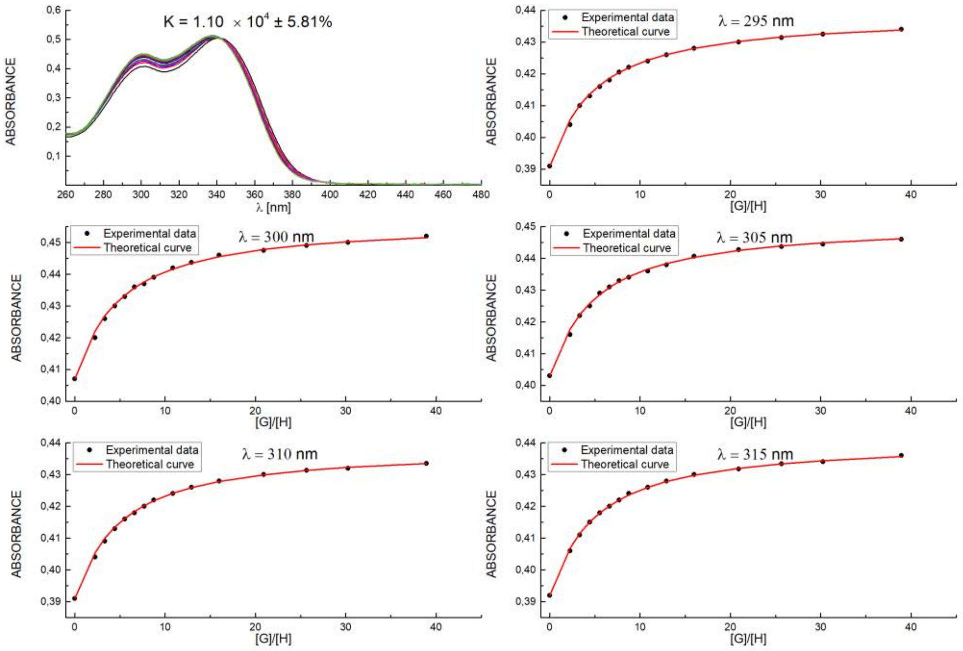

Fig. S8. UV-Vis titration of receptor **2** with $TBANO_3$ in the presence of 3 equivalents of KPF_6 and selecting binding isothermes.

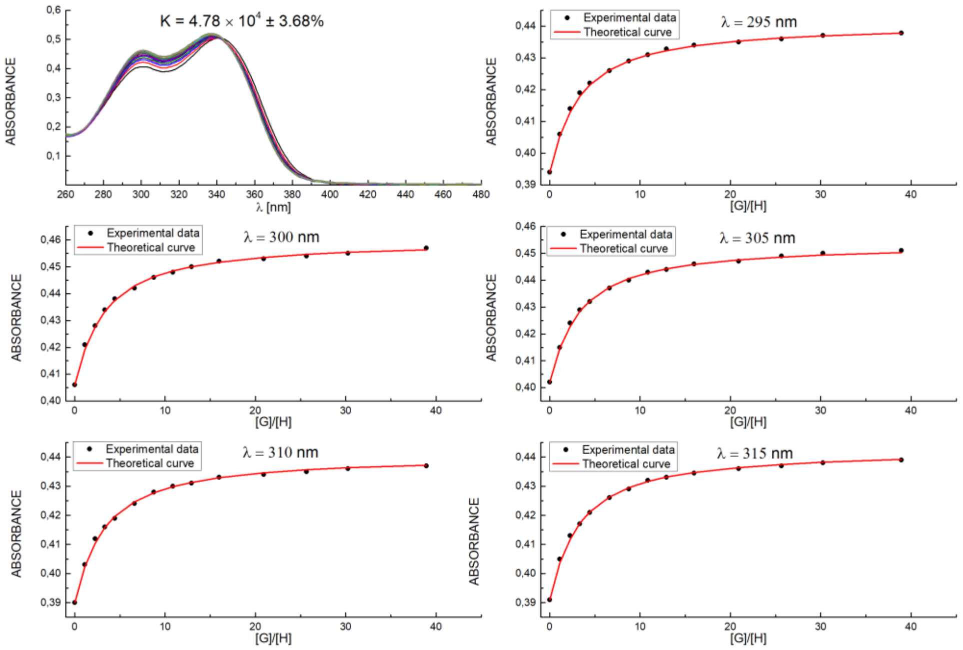

Fig. S9. UV-Vis titration of receptor **2** with TBA_2SO_4 and selecting binding isothermes.

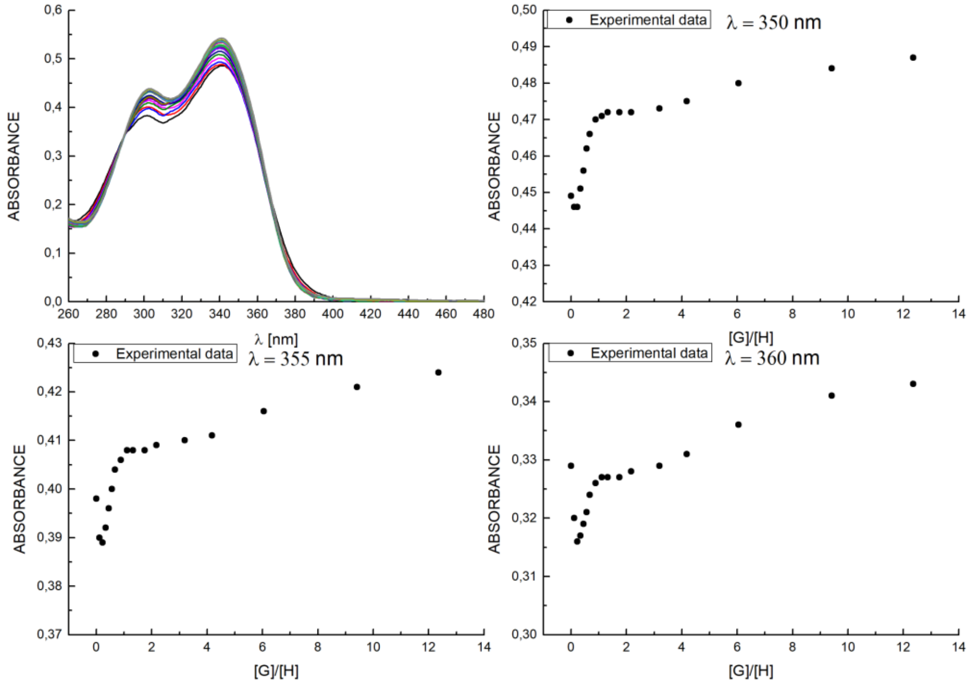

Fig. S10. UV-Vis titration of receptor **2** with TBA_2SO_4 in the presence of 3 equivalents of $NaClO_4$ and selecting binding isothermes.

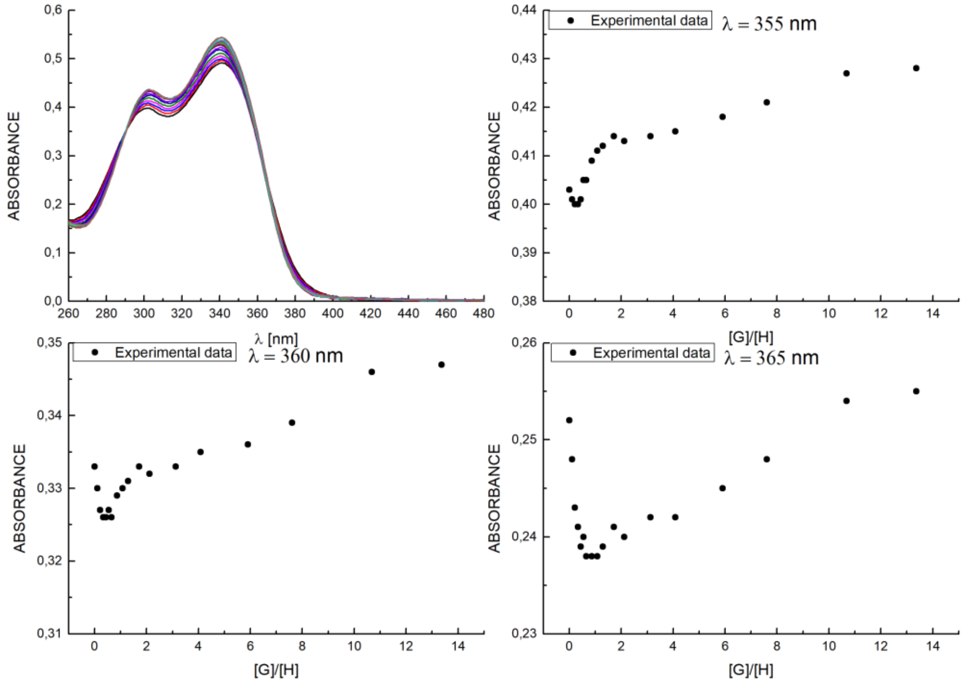

Fig. S11. UV-Vis titration of receptor **2** with TBA_2SO_4 in the presence of 3 equivalents of KPF_6 and selecting binding isothermes.

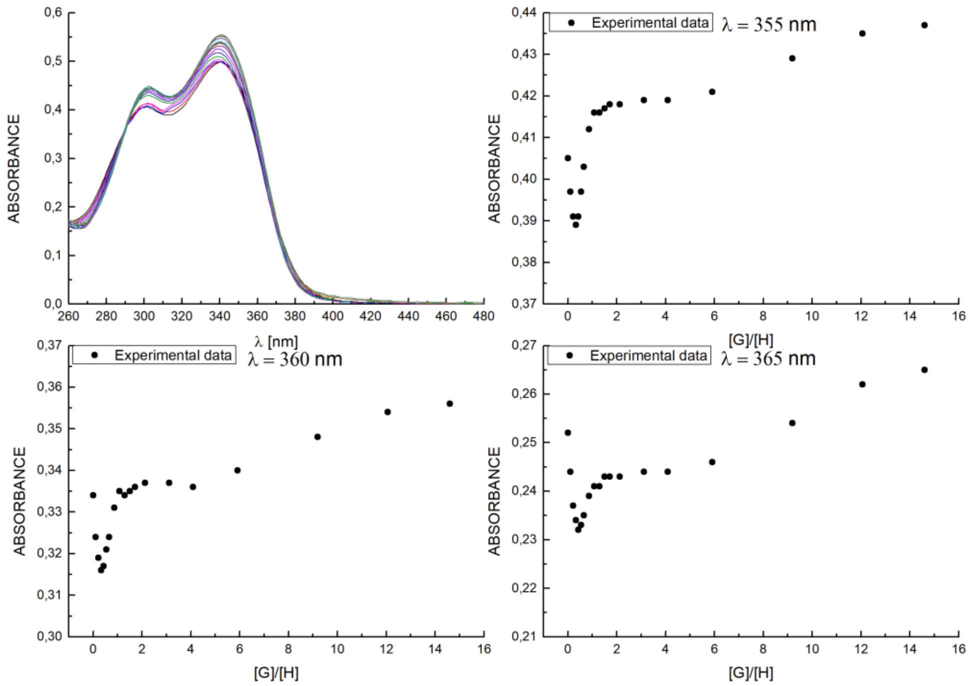

Fig. S12. UV-Vis titration of receptor **3** with TBACl and selecting binding isothermes.

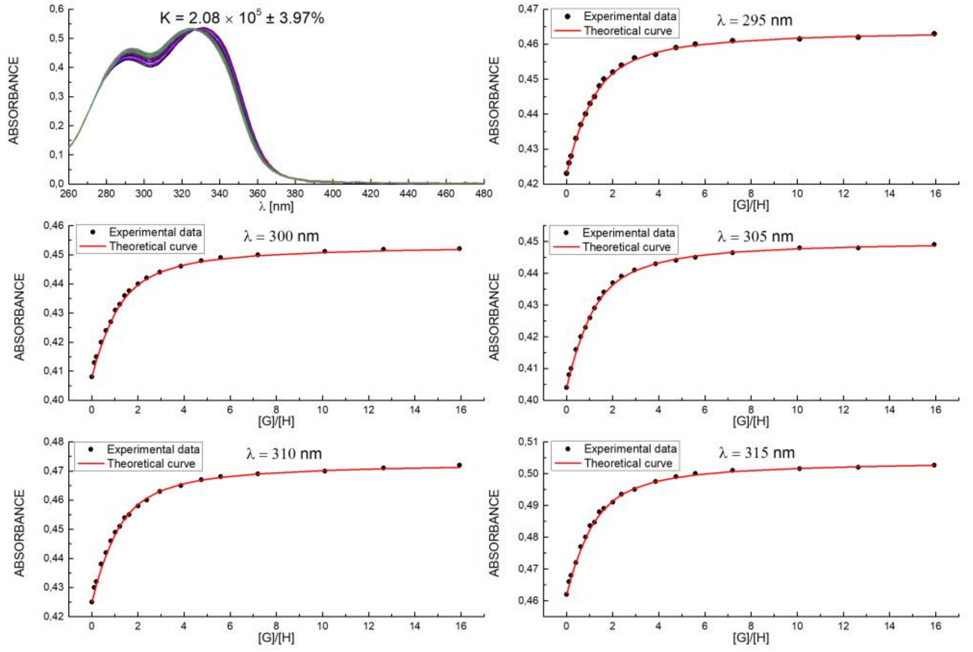

Fig. S13. UV-Vis titration of receptor **3** with TBACl in the presence of 3 equivalents of $NaClO_4$ and selecting binding isothermes.

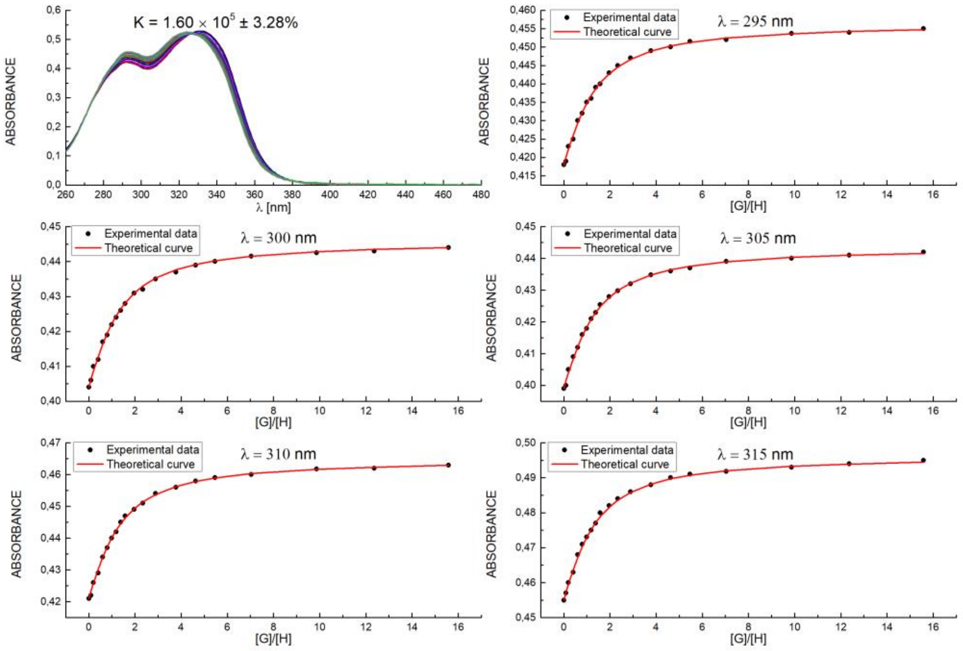

Fig. S14. UV-Vis titration of receptor **3** with TBACl in the presence of 3 equivalents of KPF_6 and selecting binding isothermes.

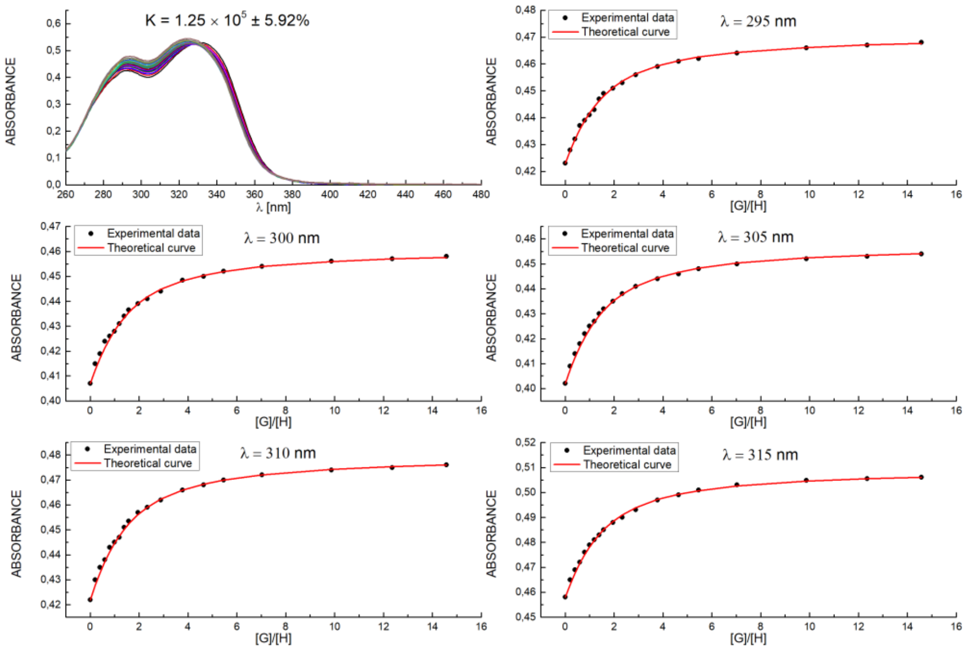

Fig. S15. UV-Vis titration of receptor **4** with TBACl and selecting binding isothermes.

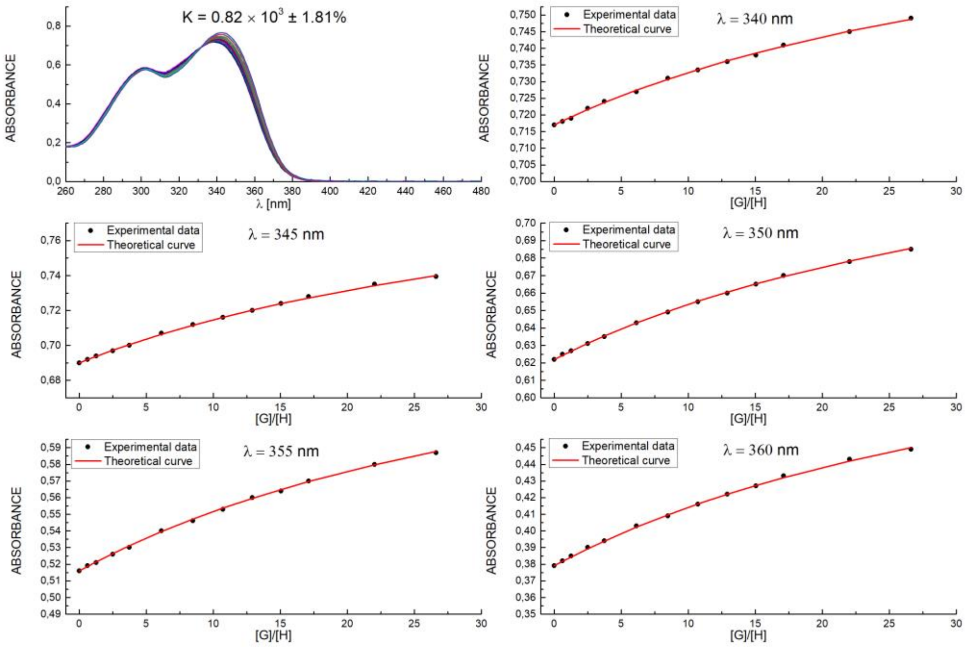

Fig. S16. UV-Vis titration of receptor **4** with TBACl in the presence of 1 equivalent of $NaClO_4$ and selecting binding isothermes.

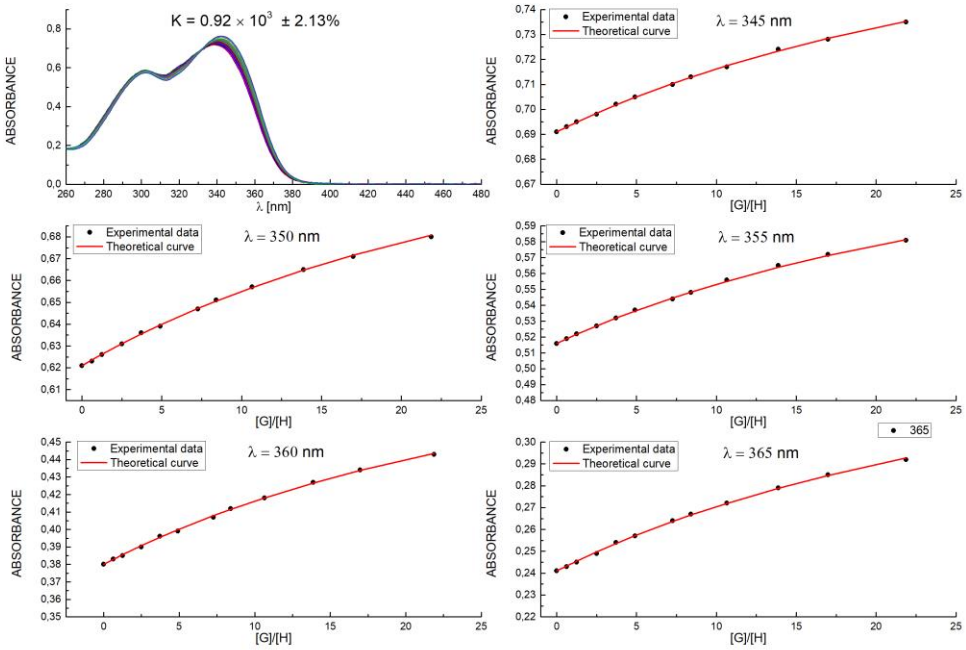

Fig. S17. UV-Vis titration of receptor **4** with TBACl in the presence of 1 equivalent of KPF_6 and selecting binding isothermes.

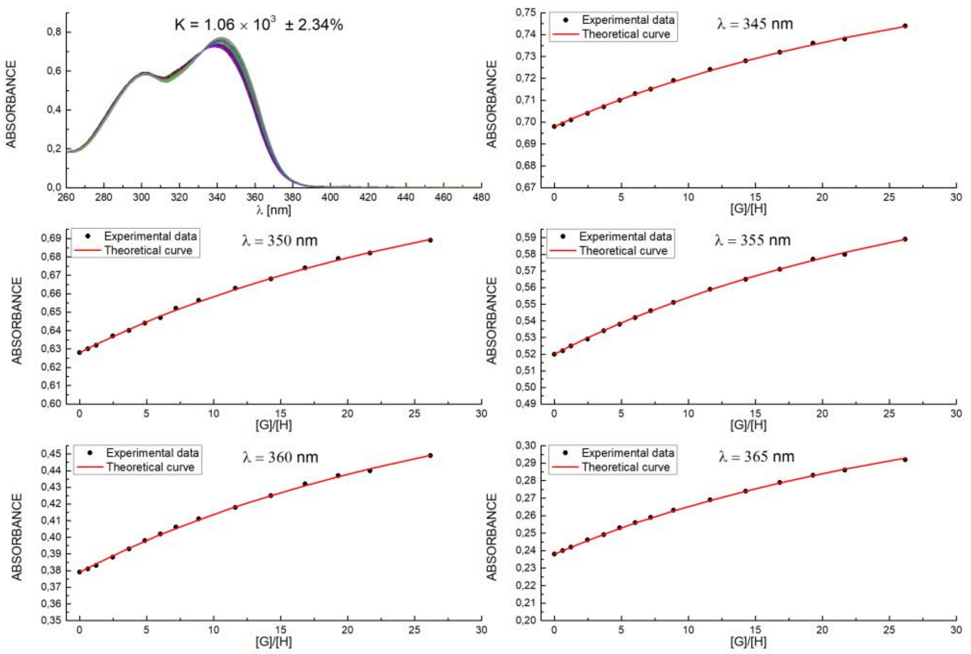

NMR TITRATION

The ^{1}H NMR titration was performed on a Bruker 300 spectrometer, at 298K in DMSO-d_6. In each case, a 500 μL of freshly prepared 2.55 mM solution of receptors was added to a 5mm NMR tube. In the case of ion pair titration receptor **1, 2** and **3** was firstly pretreated with three equivalent of KPF_6 or $NaClO_4$ (refers to receptor). In the event of ion pair titration receptor **4** was firstly pretreated with one equivalent of KPF_6 or $NaClO_4$. Then small aliquots of solution of TBAX, containing receptors at constant concentration and in case of **1, 2, 3** three equivalent of KPF_6 or $NaClO_4$ and one equivalent of this salts in the case of **4**, were added and a spectrum was acquired after each addition. The resulting titration data were analyzed using BindFit (v0.5) package, available online at http://supramolecular.org

Fig. S18. Dilution ^{1}H NMR spectra of receptor **2** in DMSO-d_6.

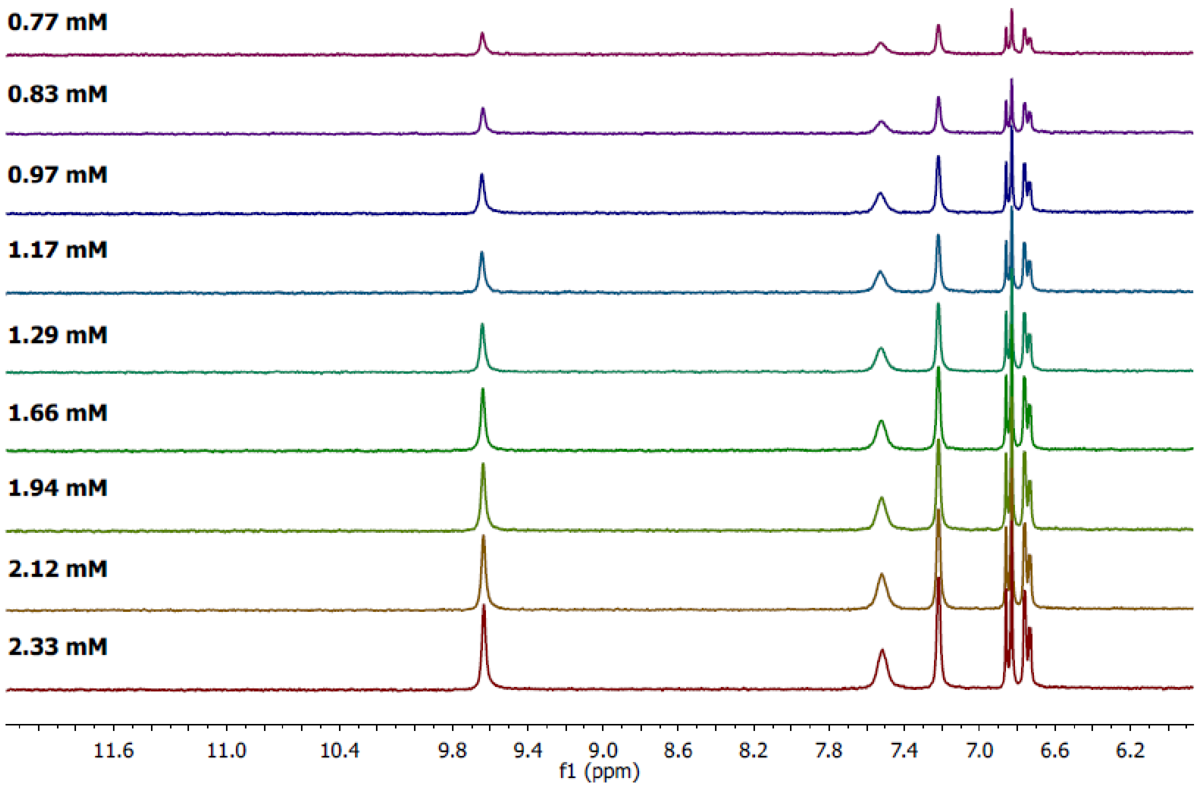

Fig. S19. ^{1}H NMR spectra recorded upon titration of receptor **1** in DMSO-d_6 with TBACl.

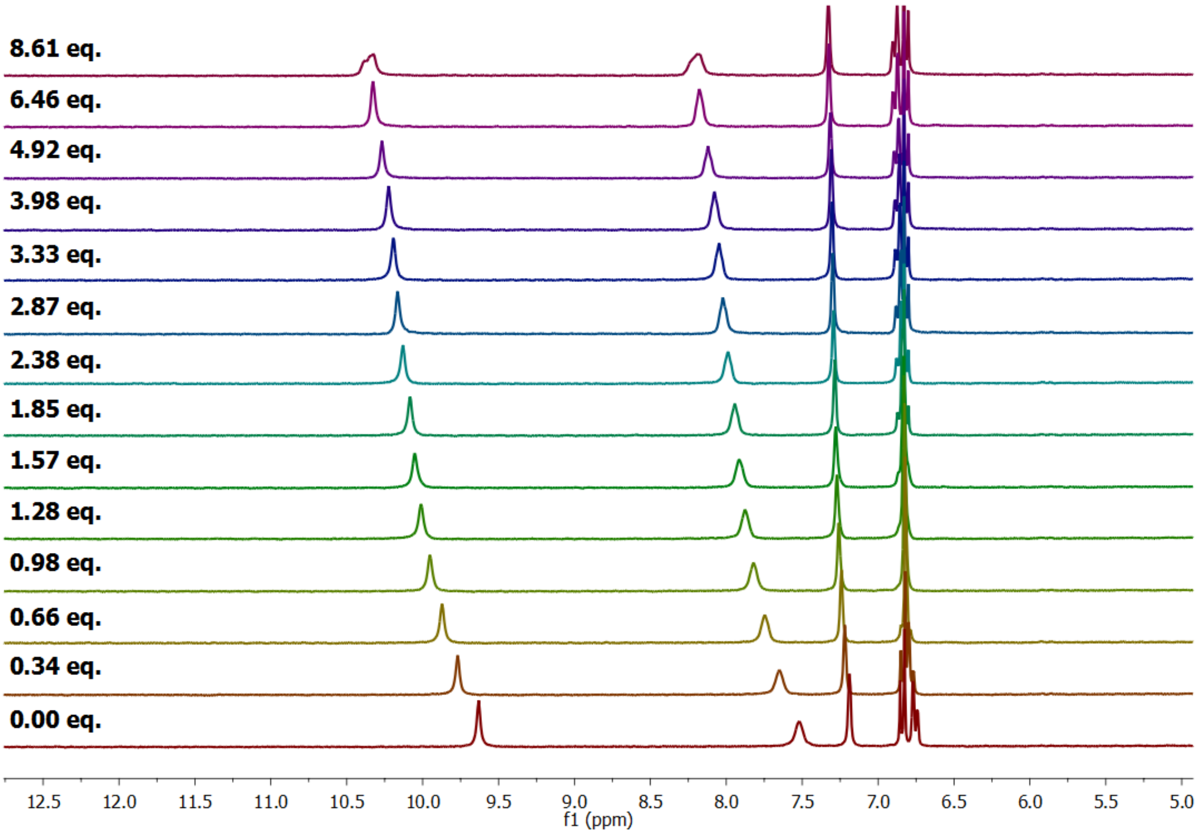

Fig. S20. ^{1}H NMR titration binding isotherms of receptor **1** in DMSO-d_6 upon addition of increasing amounts of TBACl.

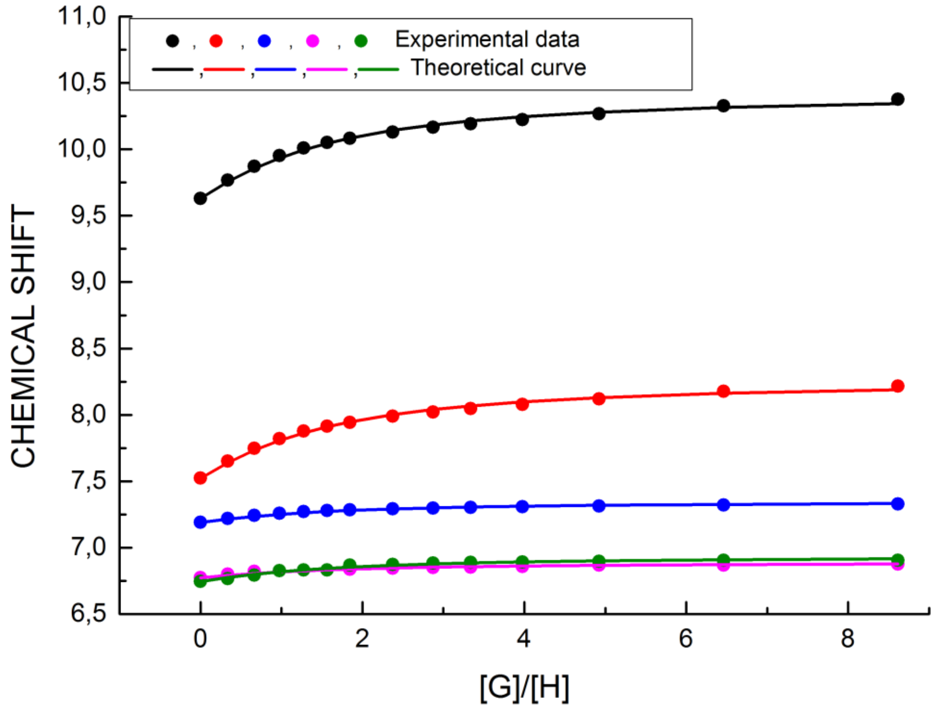

Fig. S21. ^{1}H NMR spectra recorded upon titration of receptor **1** in DMSO-d_6 with TBACl in the presence of 3 eq. $NaClO_4$.

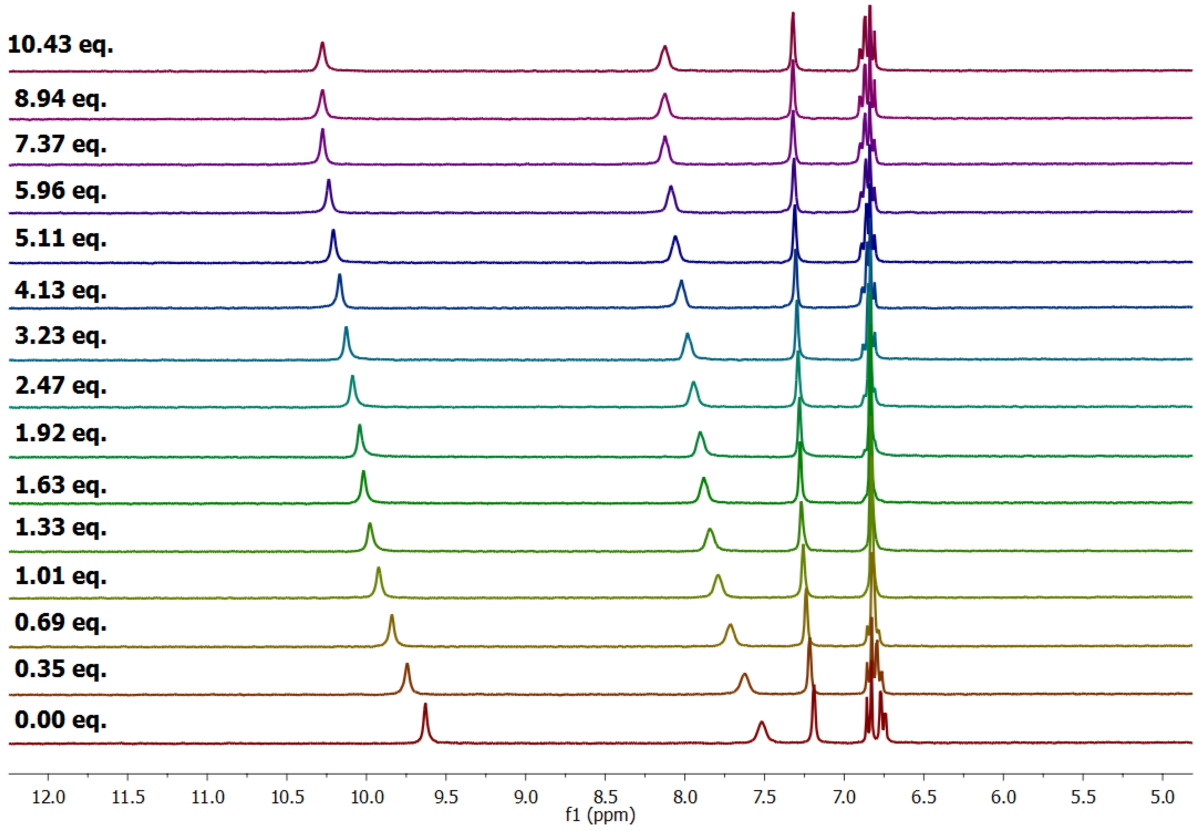

Fig. S22. ^{1}H NMR titration binding isotherms of receptor **1** in DMSO-d_6 upon addition of increasing amounts of TBACl the presence of 3 eq. $NaClO_4$.

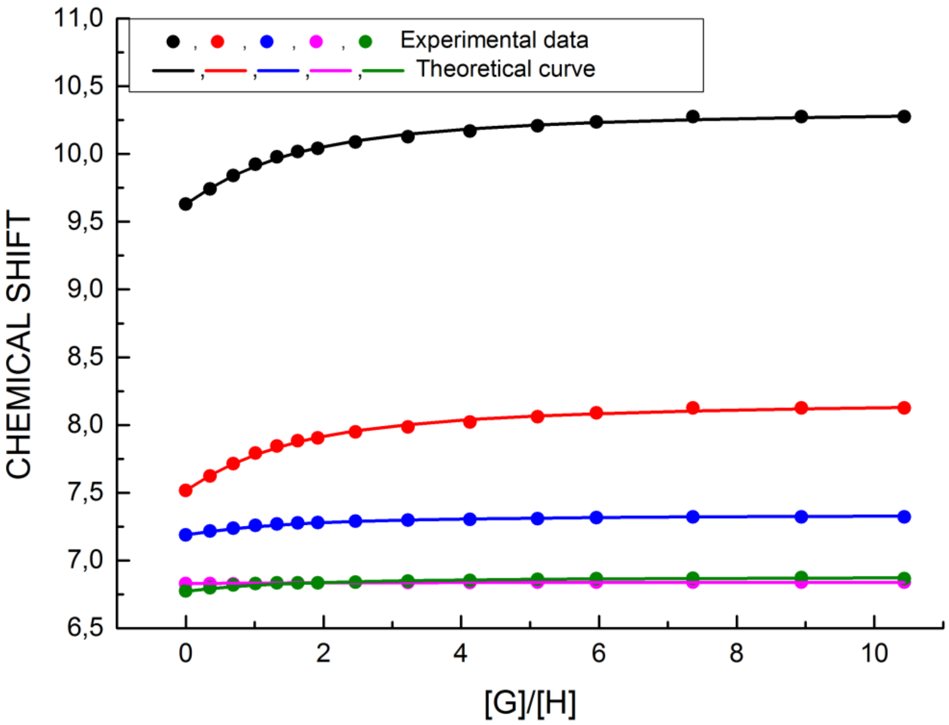

Fig. S23. ^{1}H NMR spectra recorded upon titration of receptor **2** in DMSO-d_6 with TBACl.

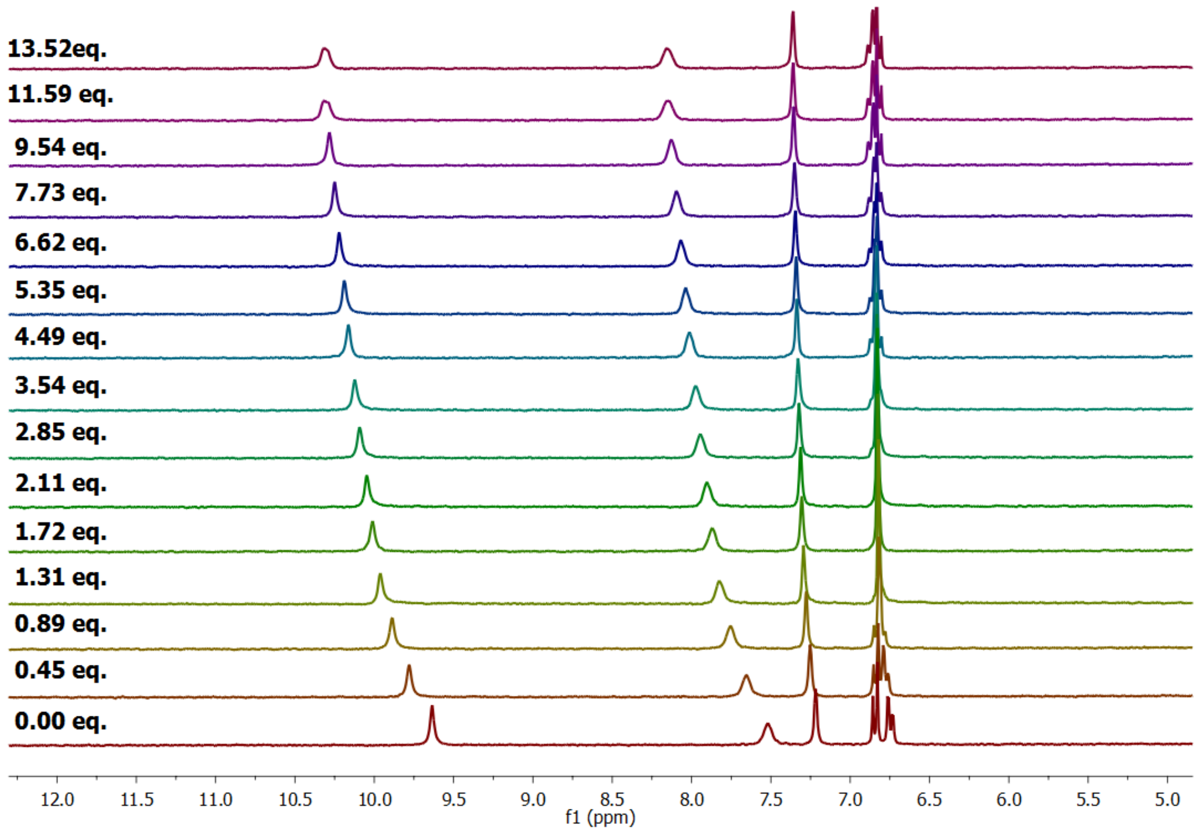

Fig. S24. ^{1}H NMR titration binding isotherms of receptor **2** in DMSO-d_6 upon addition of increasing amounts of TBACl.

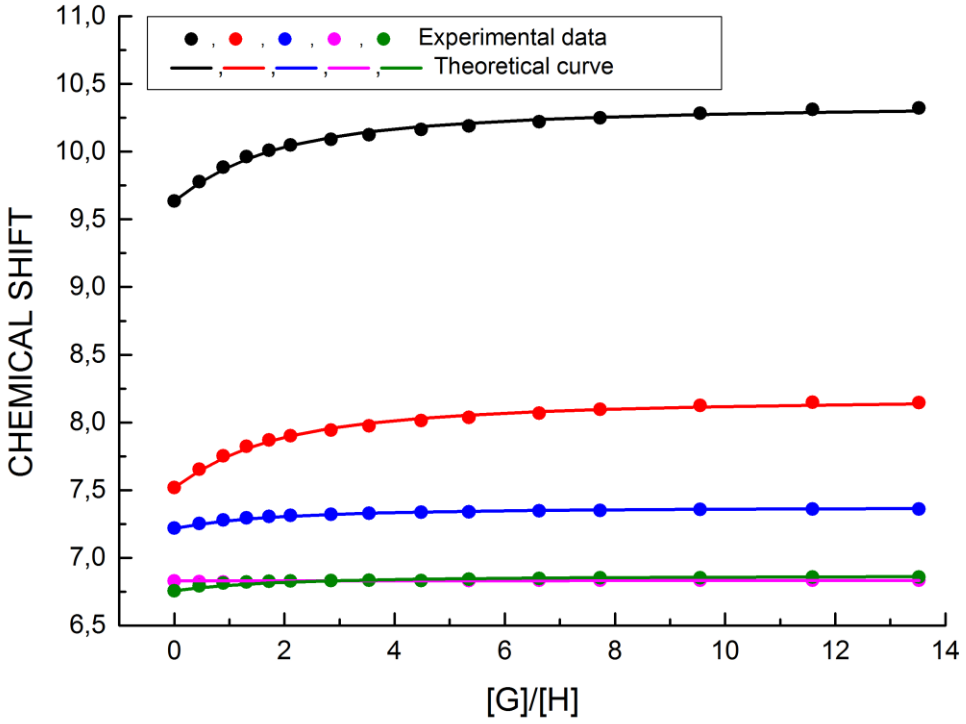

Fig. S25. ^{1}H NMR spectra recorded upon titration of receptor **2** in DMSO-d_6 with TBACl in the presence of 3 eq. $NaClO_4$.

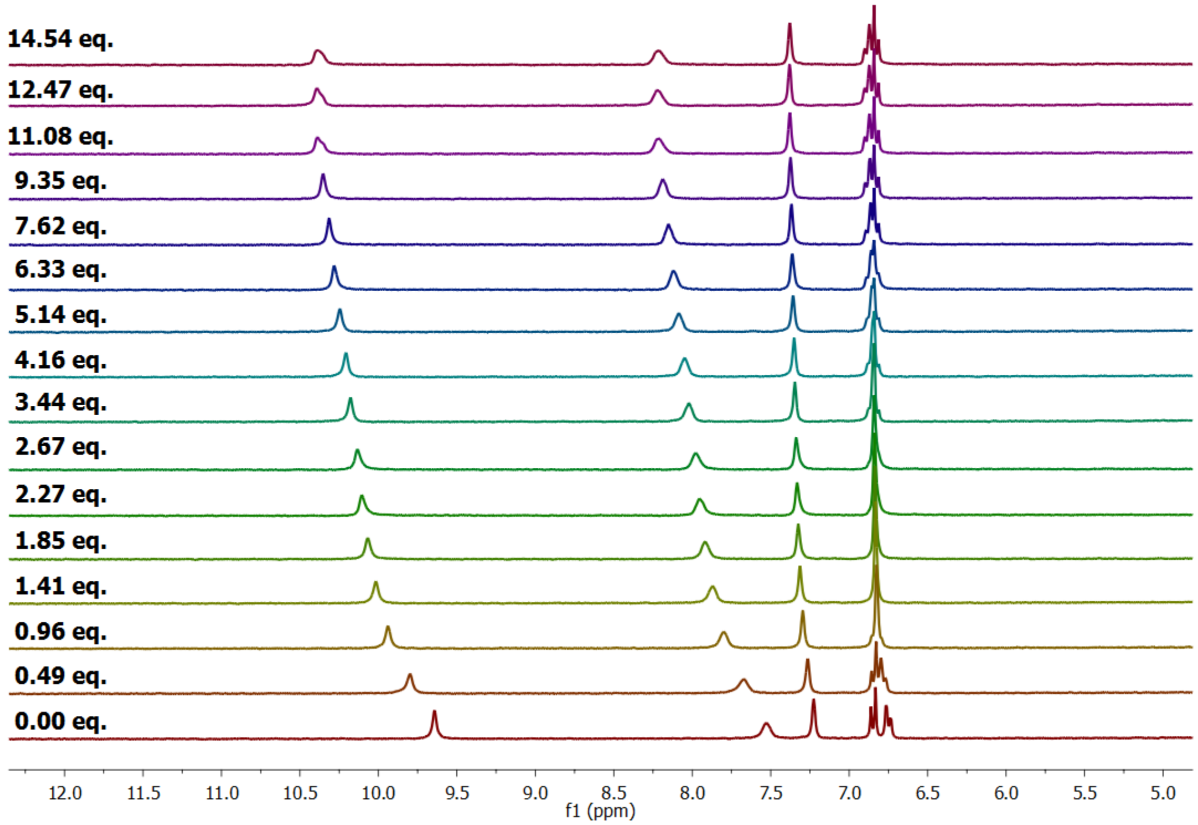

Fig. S26. ^{1}H NMR titration binding isotherms of receptor **2** in DMSO-d_6 upon addition of increasing amounts of TBACl the presence of 3 eq. $NaClO_4$.

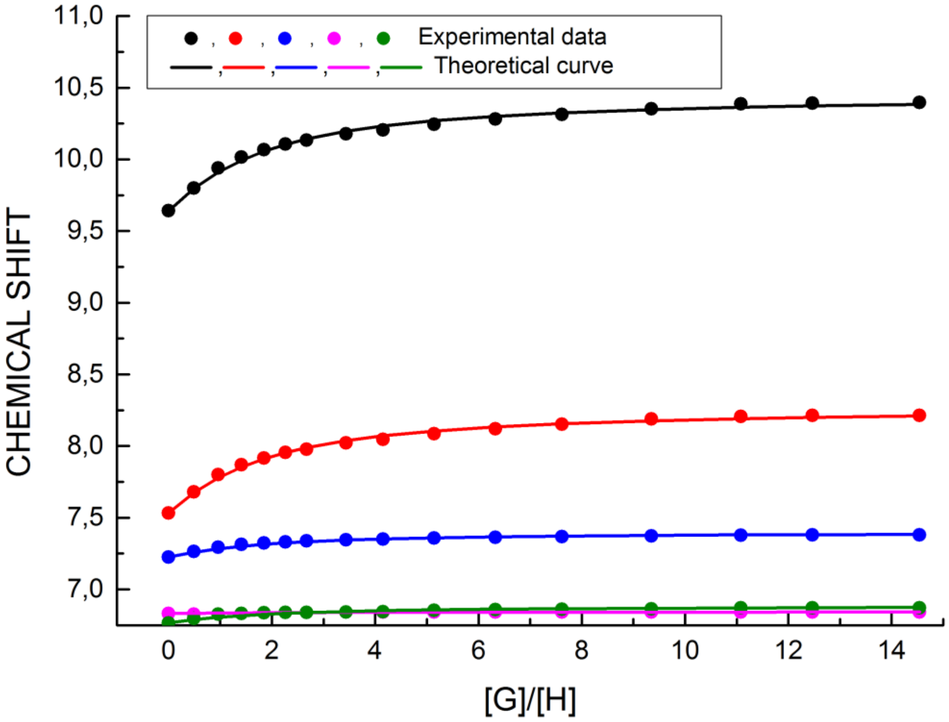

Fig. S27. ^{1}H NMR spectra recorded upon titration of receptor **2** in DMSO-d_6 with TBACl in the presence of 3 eq. KPF_6.

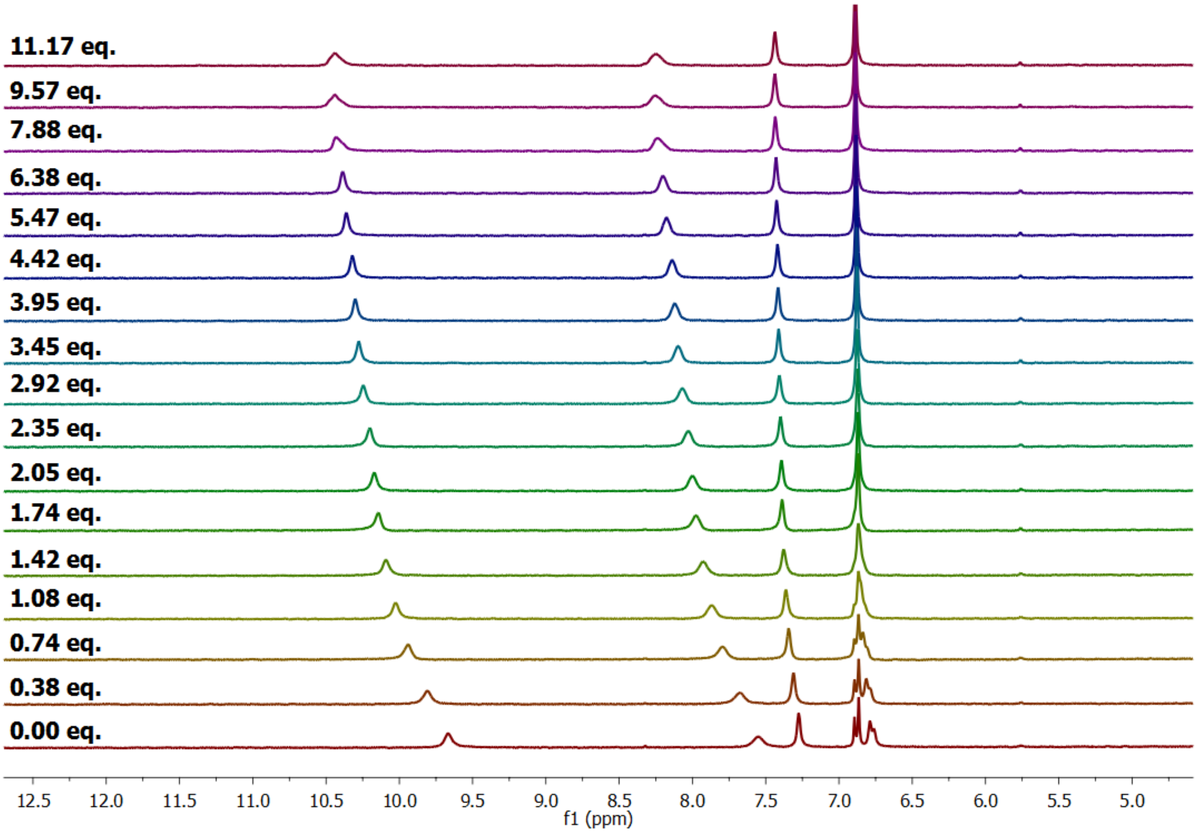

Fig. S28. ^{1}H NMR titration binding isotherms of receptor **2** in DMSO-d_6 upon addition of increasing amounts of TBACl the presence of 3 eq. KPF_6.

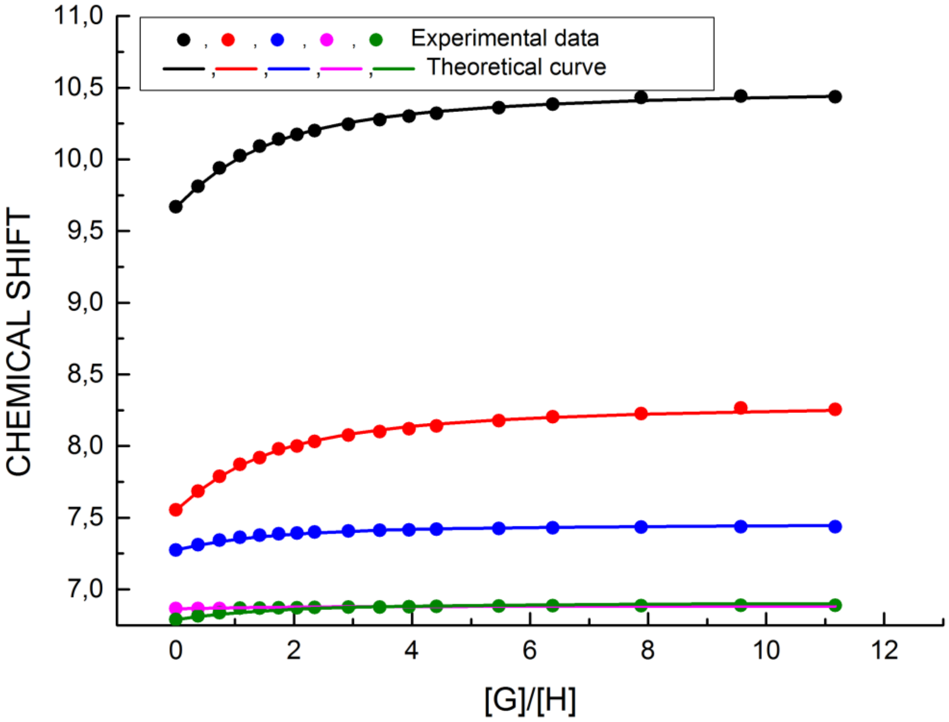

Fig. S29. ^{1}H NMR spectra recorded upon titration of receptor **2** in DMSO-d_6 with TBABr.

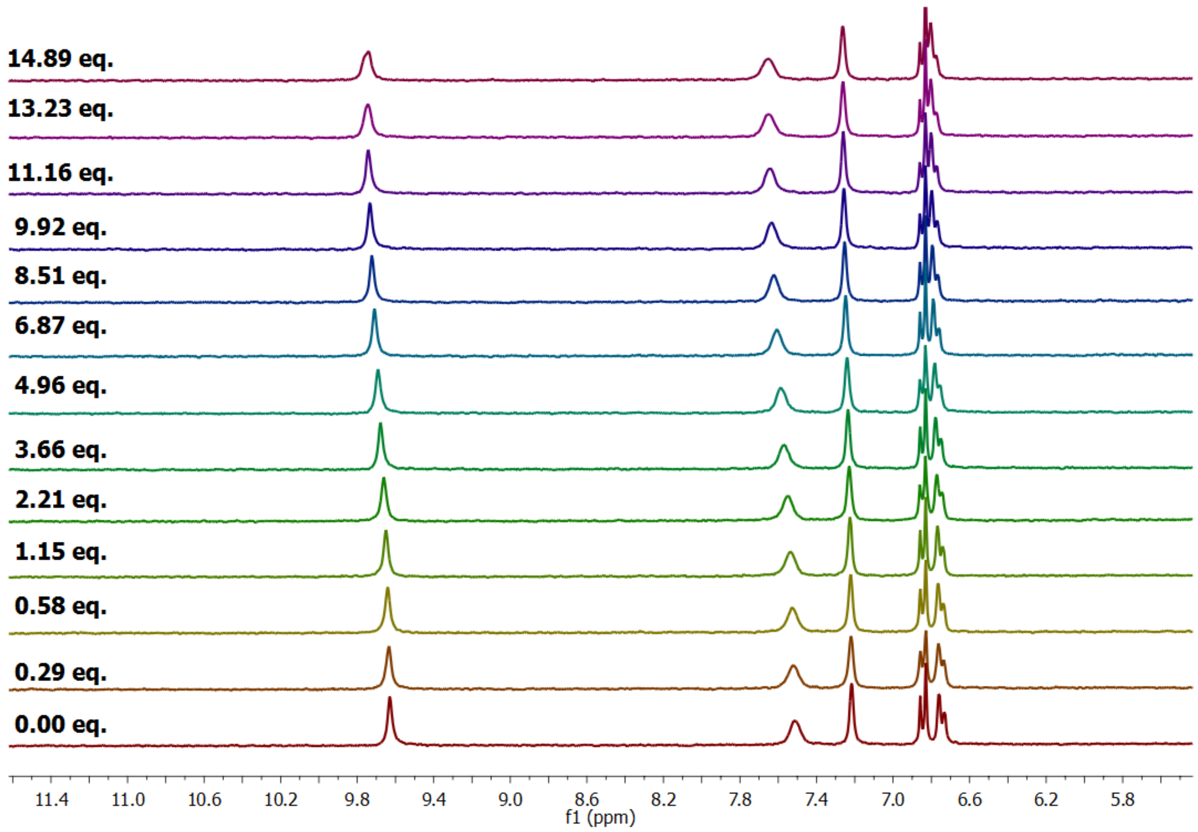

Fig. S30. ^{1}H NMR titration binding isotherms of receptor **2** in DMSO-d_6 upon addition of increasing amounts of TBABr.

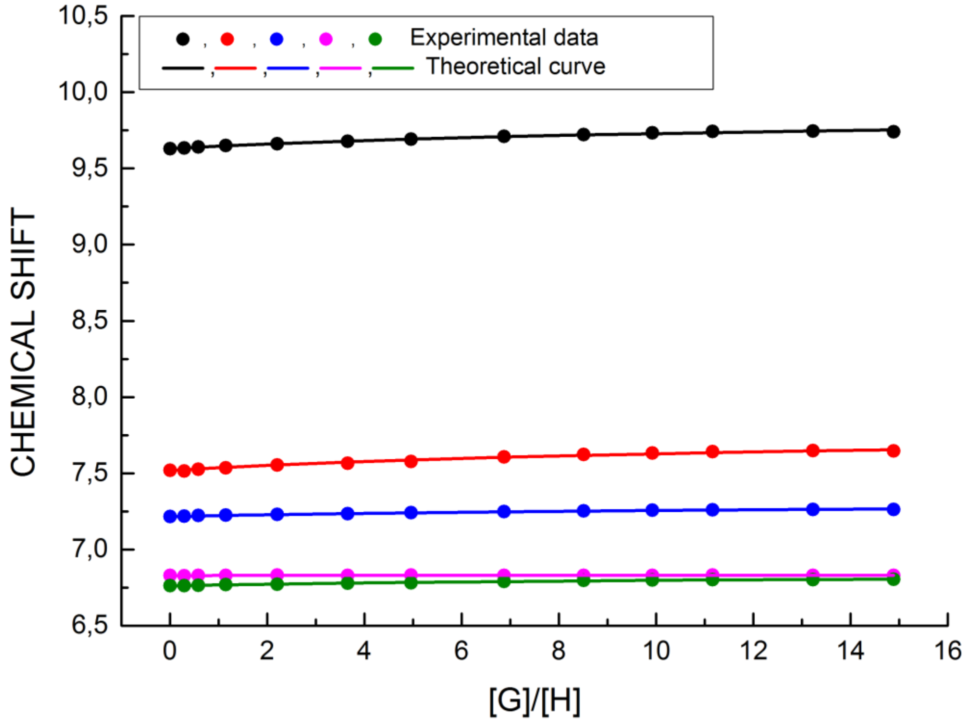

Fig. S31. ^{1}H NMR spectra recorded upon titration of receptor **2** in DMSO-d_6 with TBABr in the presence of 3 eq. KPF_6.

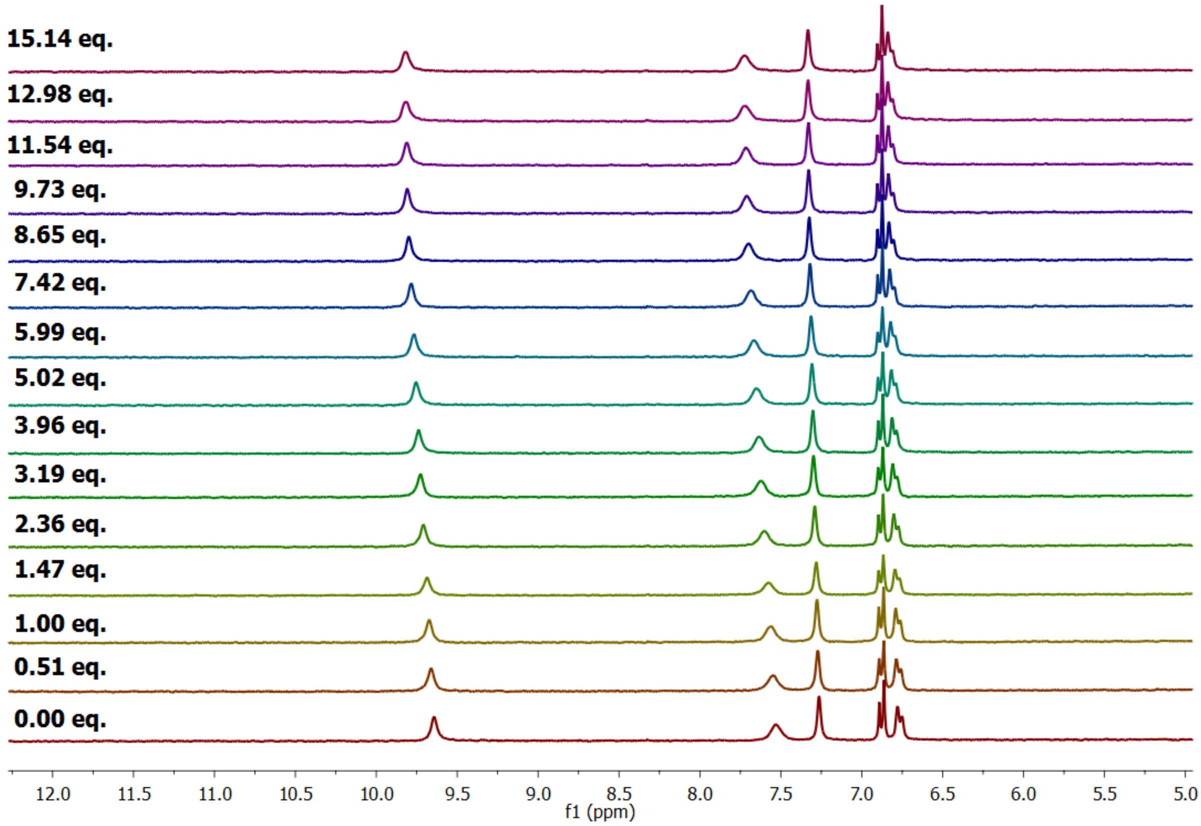

Fig. S32. ^{1}H NMR titration binding isotherms of receptor **2** in DMSO-d_6 upon addition of increasing amounts of TBABr the presence of 3 eq. KPF_6.

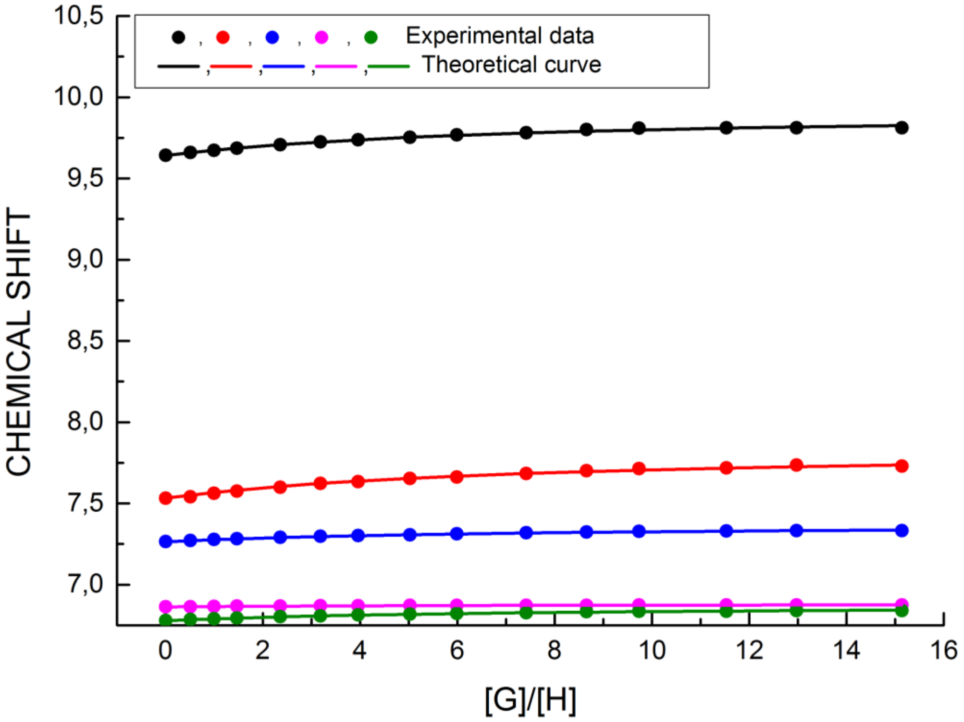

Fig. S33. ^{1}H NMR spectra recorded upon titration of receptor **2** in DMSO-d_6 with $TBANO_2$.

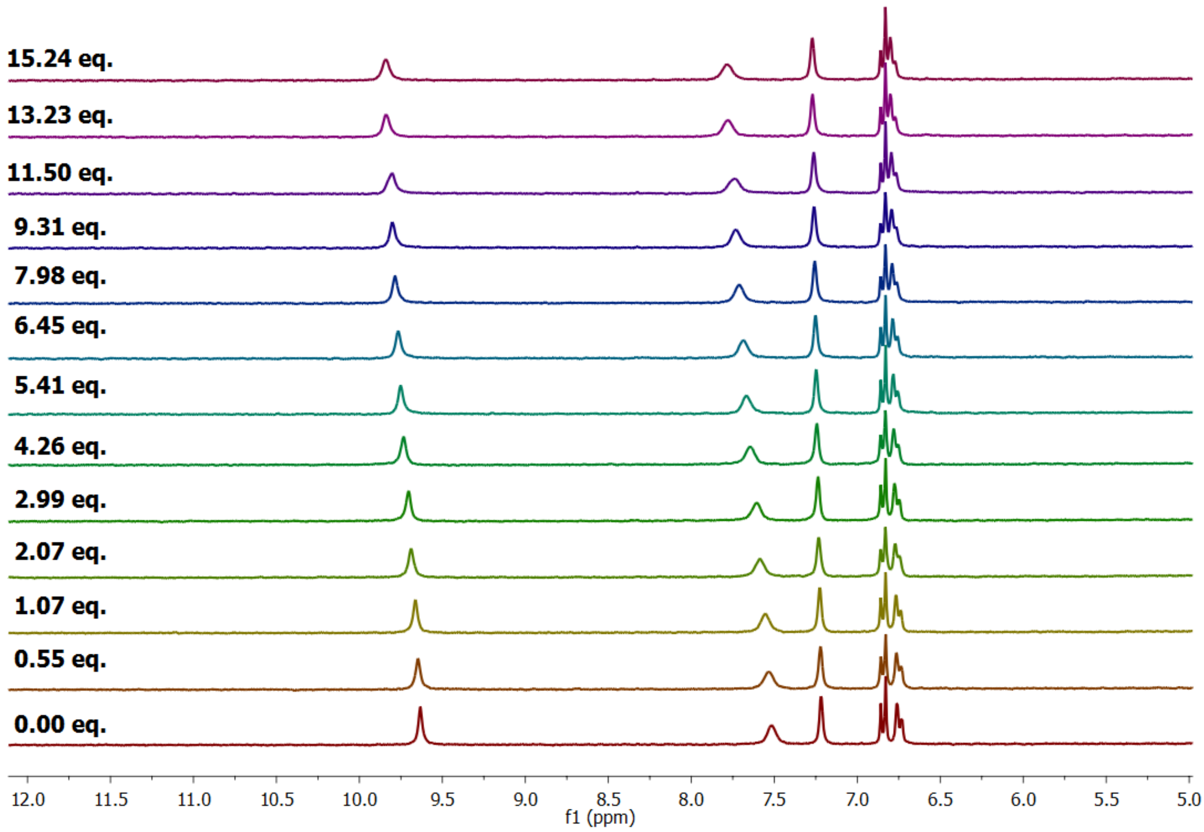

Fig. S34. ^{1}H NMR titration binding isotherms of receptor **2** in DMSO-d_6 upon addition of increasing amounts of $TBANO_2$.

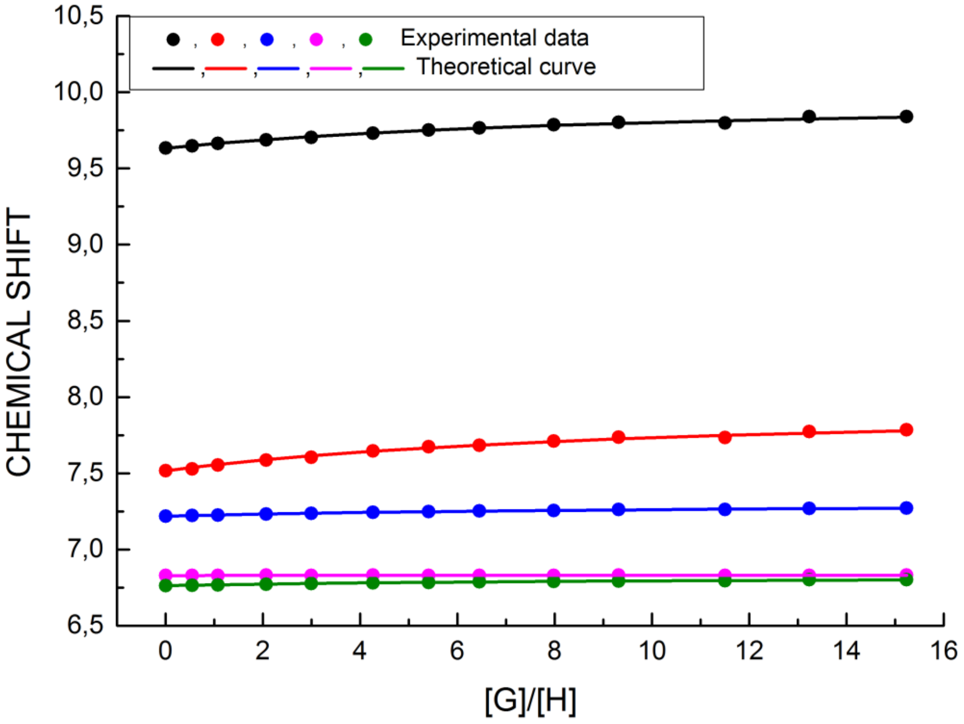

Fig. S35. ^{1}H NMR spectra recorded upon titration of receptor **2** in DMSO-d_6 with $TBANO_2$ in the presence of 3 eq. KPF_6.

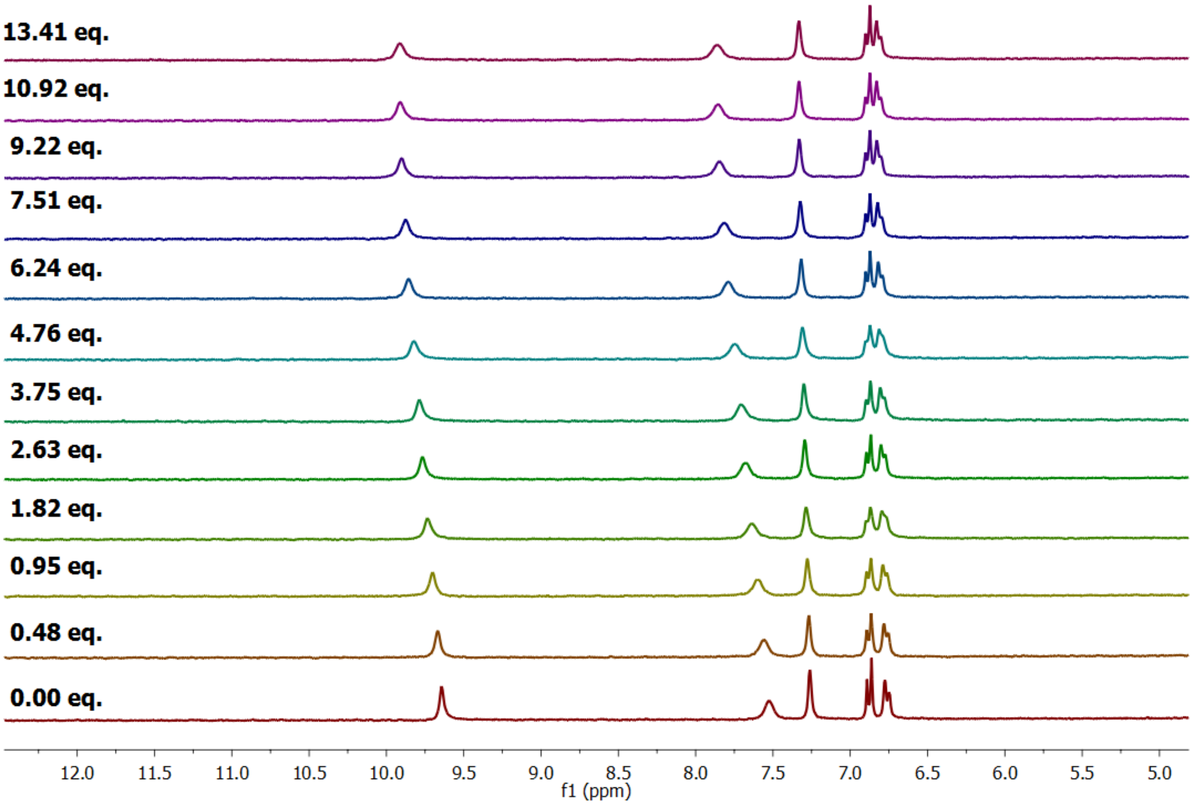

Fig. S36. ^{1}H NMR titration binding isotherms of receptor **2** in DMSO-d_6 upon addition of increasing amounts of $TBANO_2$ the presence of 3 eq. KPF_6.

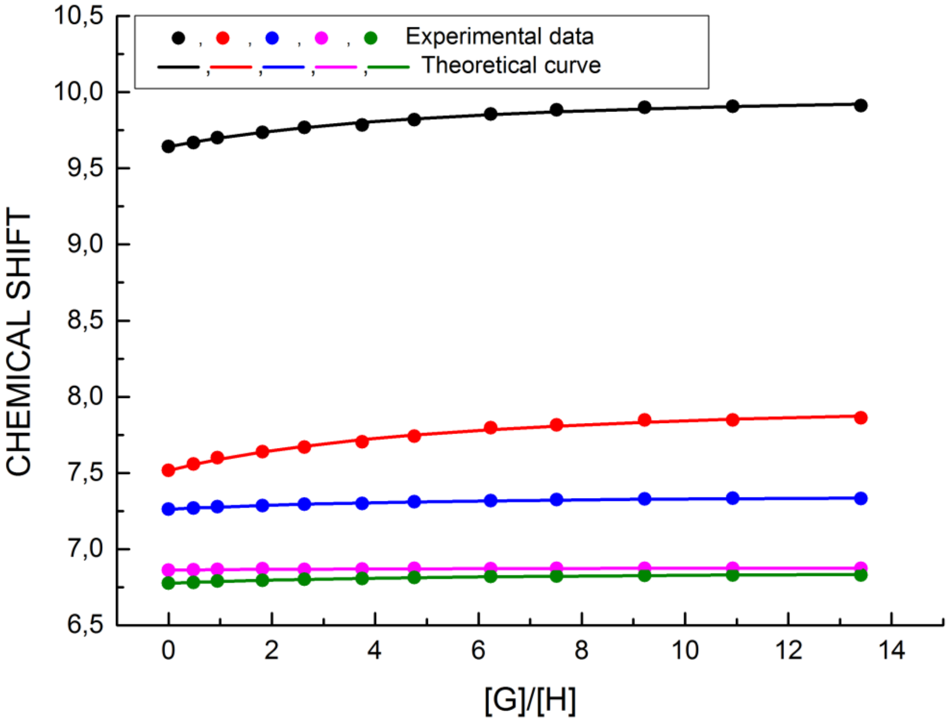

Fig. S37. ^{1}H NMR spectra recorded upon titration of receptor **2** in DMSO-d_6 with TBAPhCOO.

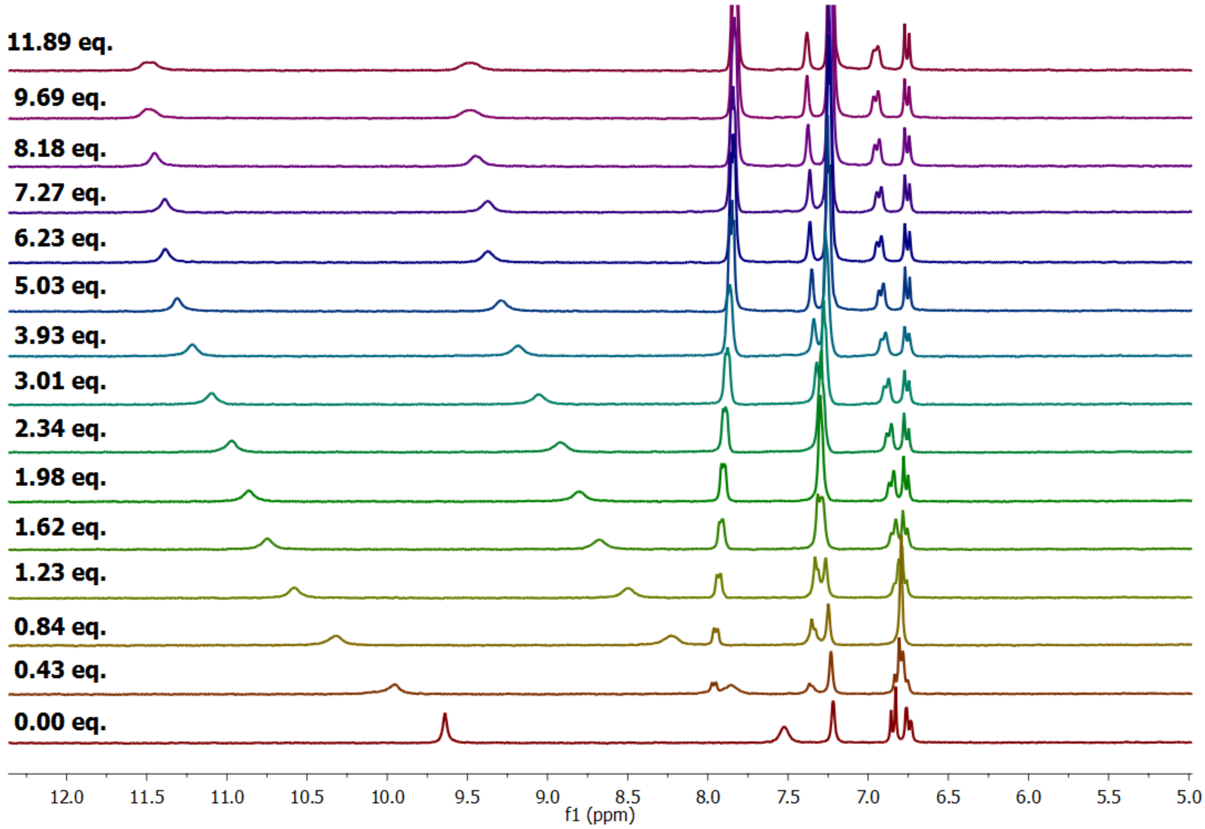

Fig. S38. ^{1}H NMR titration binding isotherms of receptor **2** in DMSO-d_6 upon addition of increasing amounts of TBAPhCOO.

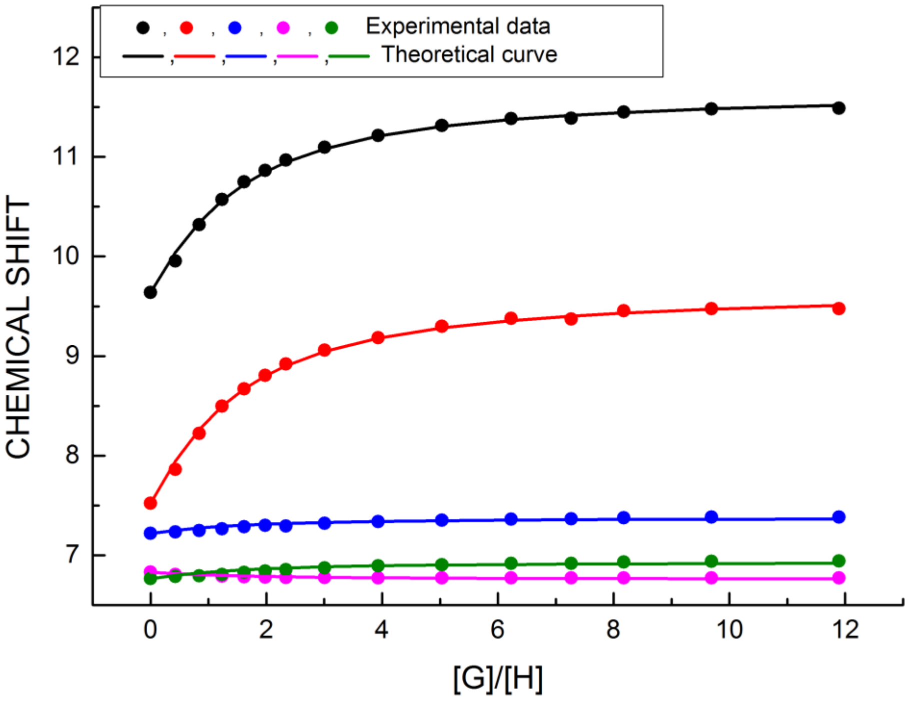

Fig. S39. ^{1}H NMR spectra recorded upon titration of receptor **2** in DMSO-d_6 with TBAPhCOO in the presence of 3 eq. KPF_6.

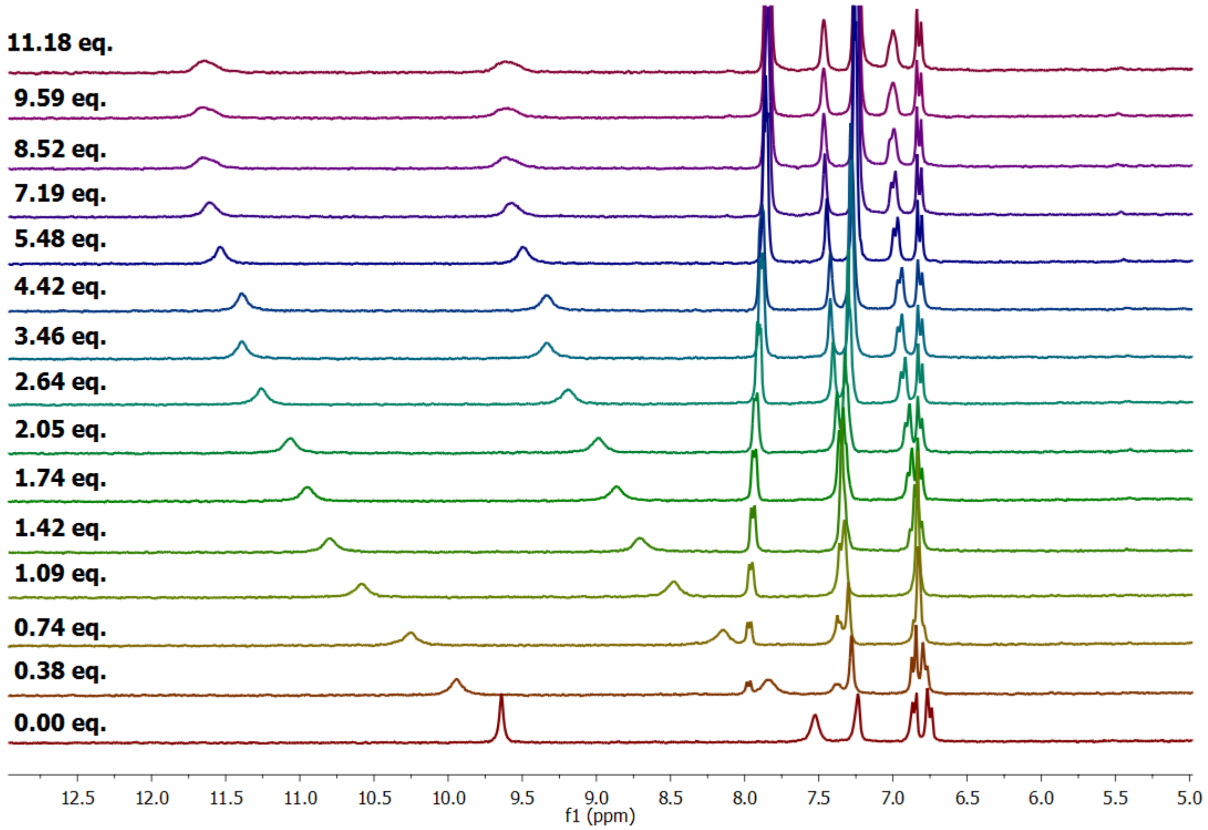

Fig. S40. ^{1}H NMR titration binding isotherms of receptor **2** in DMSO-d_6 upon addition of increasing amounts of TBAPhCOO the presence of 3 eq. KPF_6.

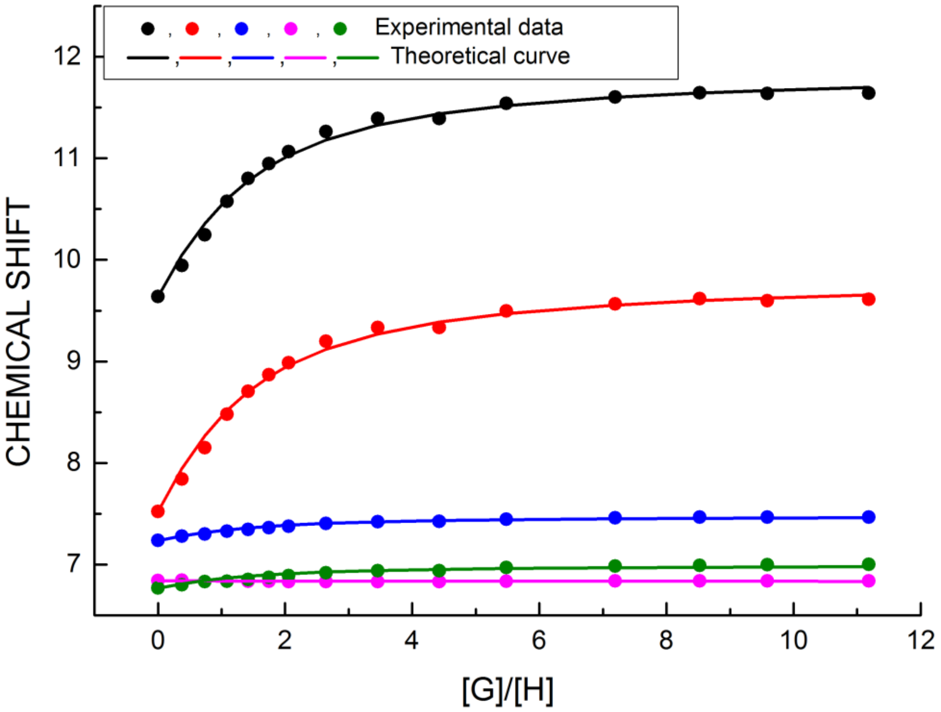

Fig. S41. ^{1}H NMR spectra recorded upon titration of receptor **2** in DMSO-d_6 with $TBACH_3COO$.

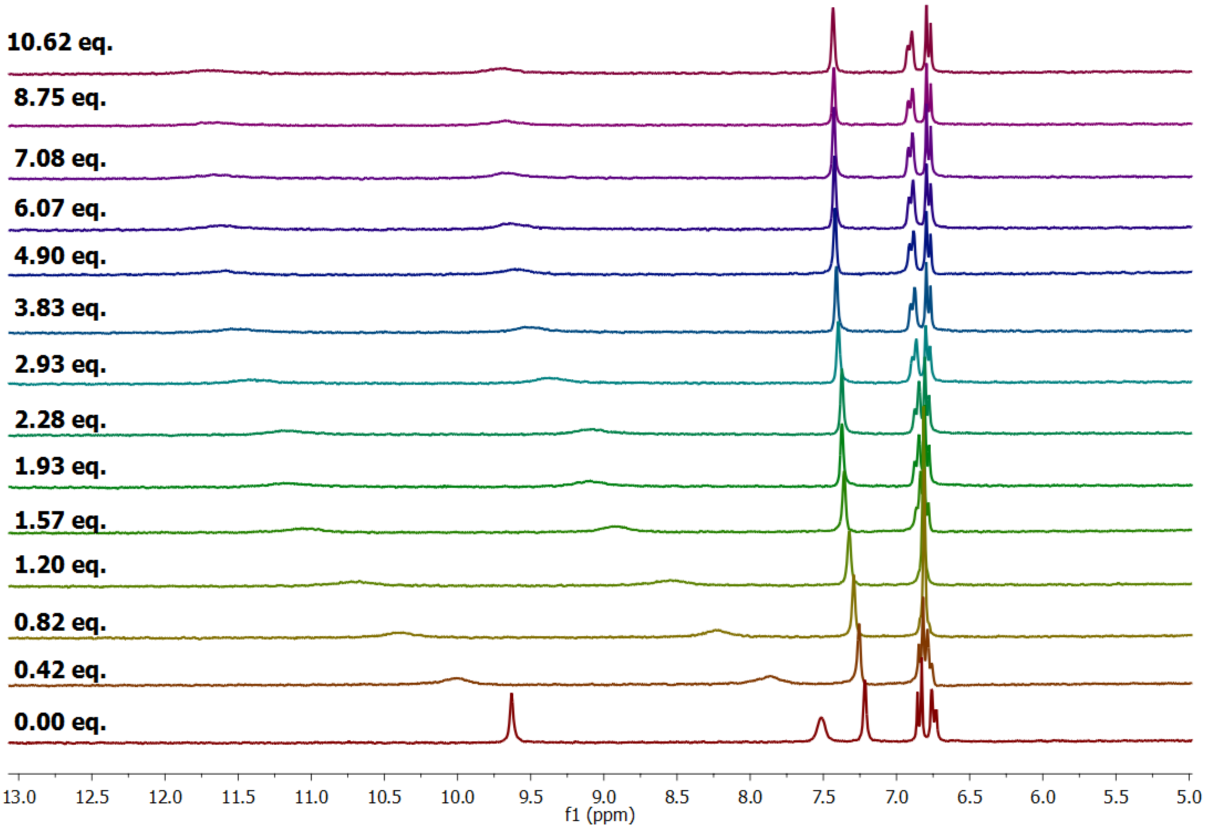

Fig. S42. ^{1}H NMR titration binding isotherms of receptor **2** in DMSO-d_6 upon addition of increasing amounts of $TBACH_3COO$.

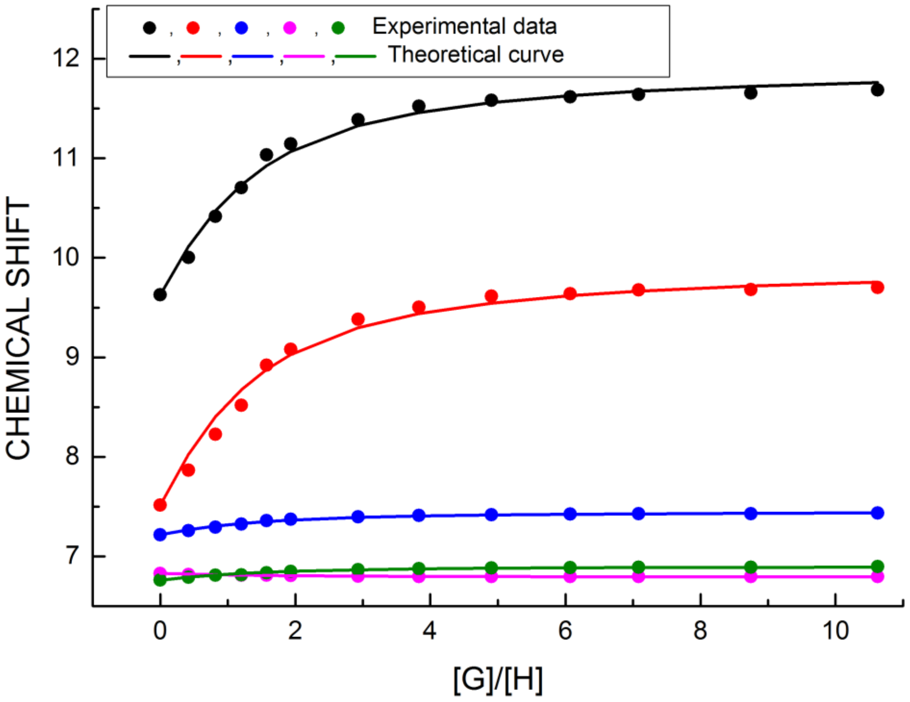

Fig. S43. ^{1}H NMR spectra recorded upon titration of receptor **2** in DMSO-d_6 with $TBACH_3COO$ in the presence of 3 eq. KPF_6.

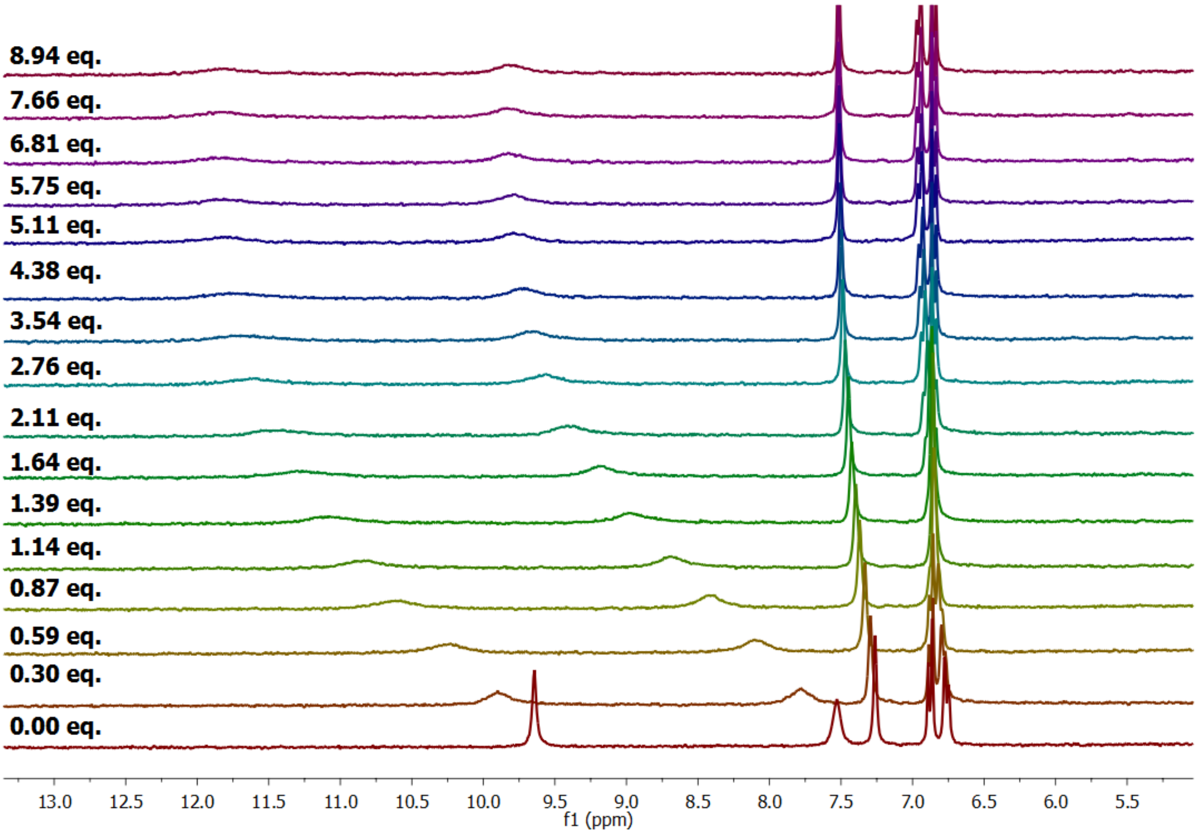

Fig. S44. ^{1}H NMR titration binding isotherms of receptor **2** in DMSO-d_6 upon addition of increasing amounts of TBACH$_3$COO the presence of 3 eq. KPF$_6$.

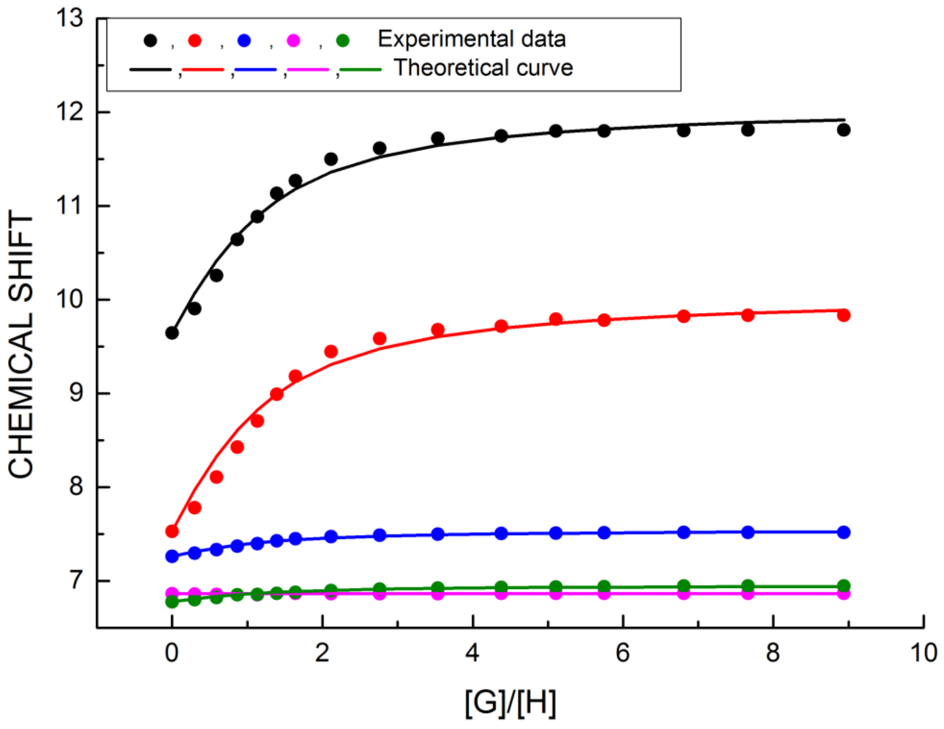

Fig. S45. ^{1}H NMR spectra recorded upon titration of receptor **2** in DMSO-d_6 with TBA$_2$SO$_4$

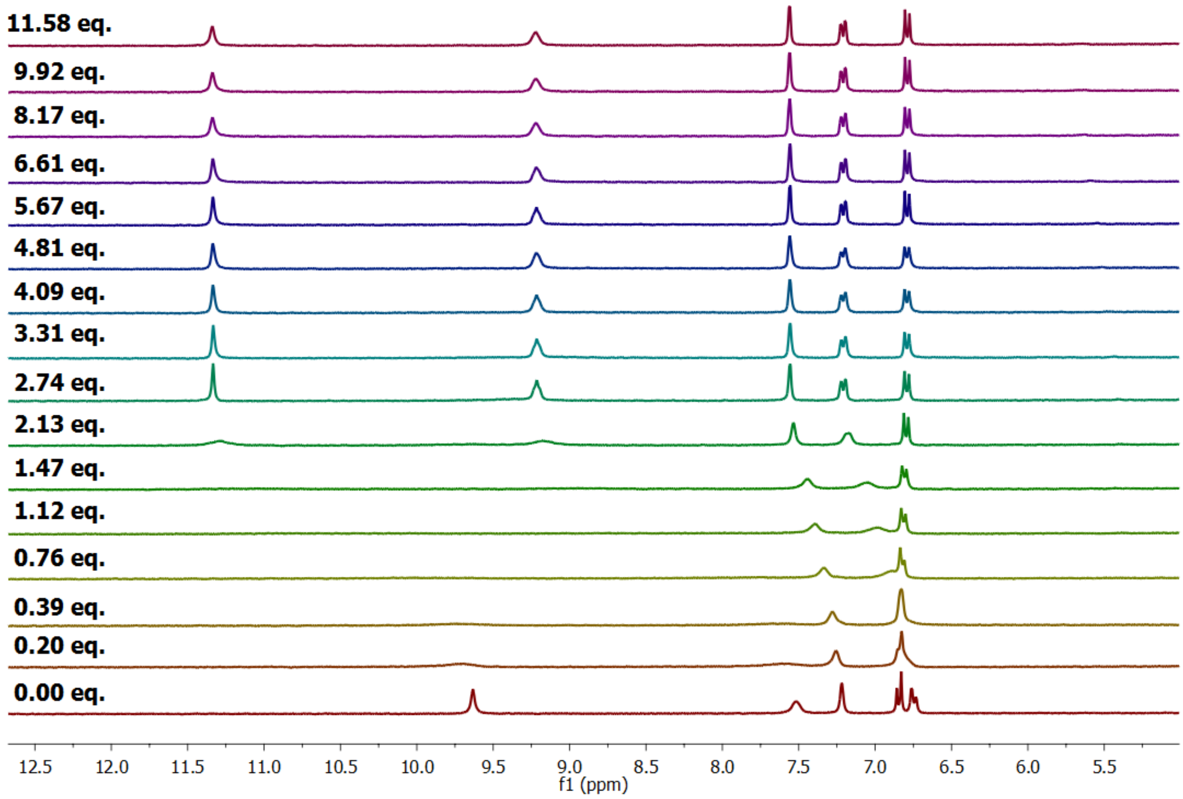

Fig. S46. ^{1}H NMR spectra recorded upon titration of receptor **2** in DMSO-d_6 with TBA_2SO_4 range from 8.00 – 6.50 ppm.

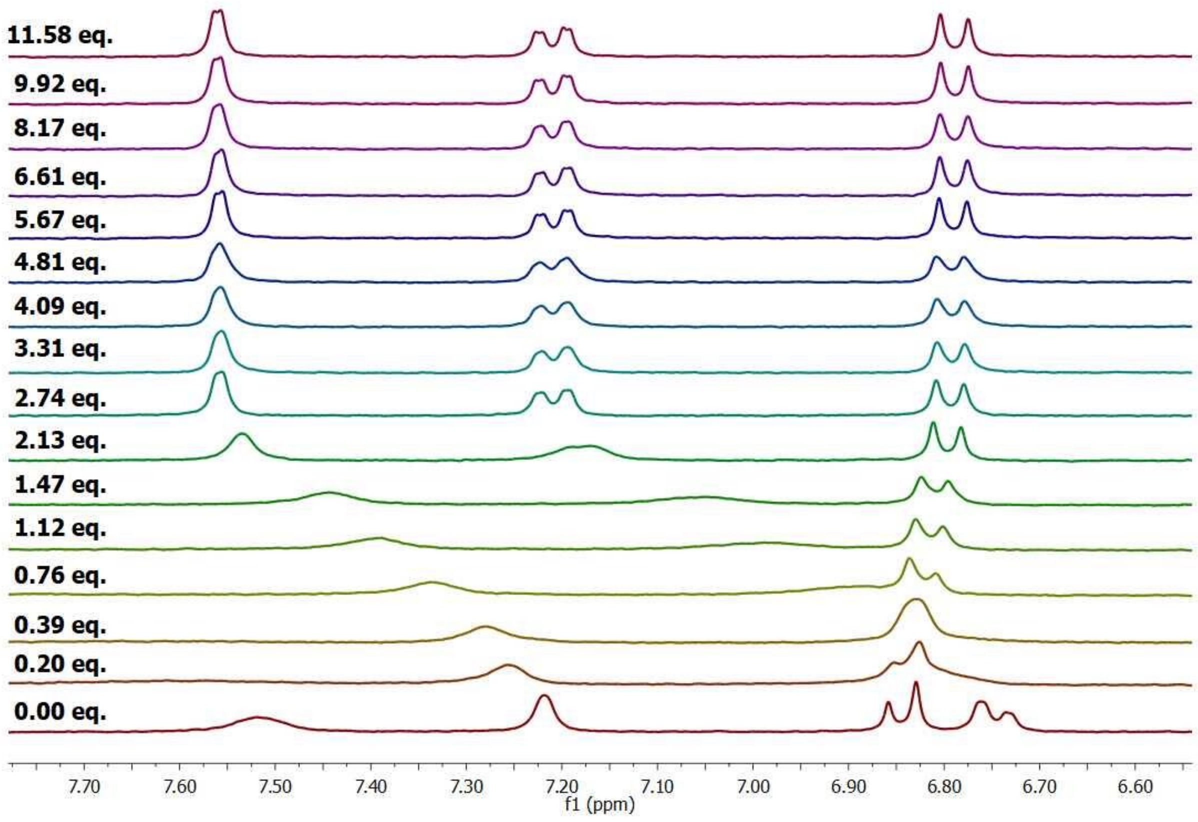

Fig. S47. ^{1}H NMR spectra recorded upon titration of receptor **2** in DMSO-d_6 with TBA_2SO_4 in the presence of 3 eq. KPF_6.

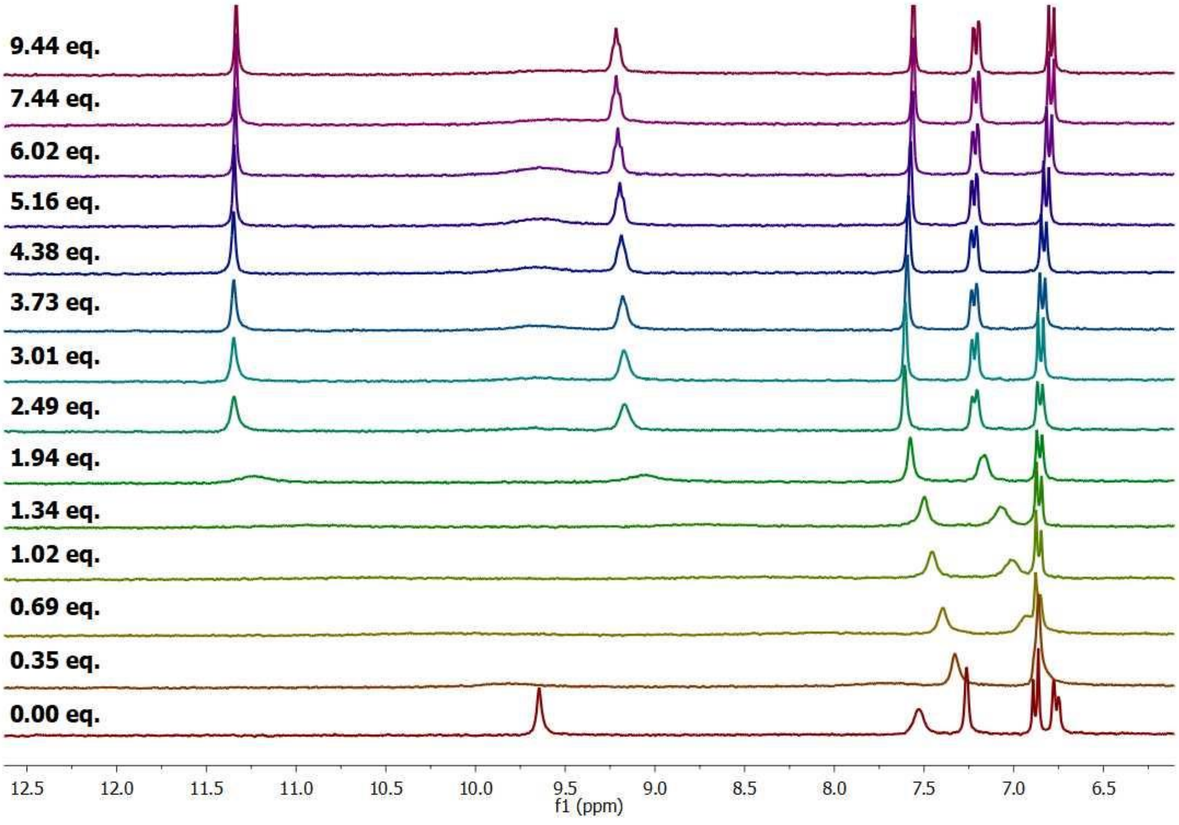

Fig. S48. ^{1}H NMR spectra recorded upon titration of receptor **2** in DMSO-d_6 with TBA_2SO_4 in the presence of 3 eq. KPF_6 range from 8.00 – 6.50 ppm.

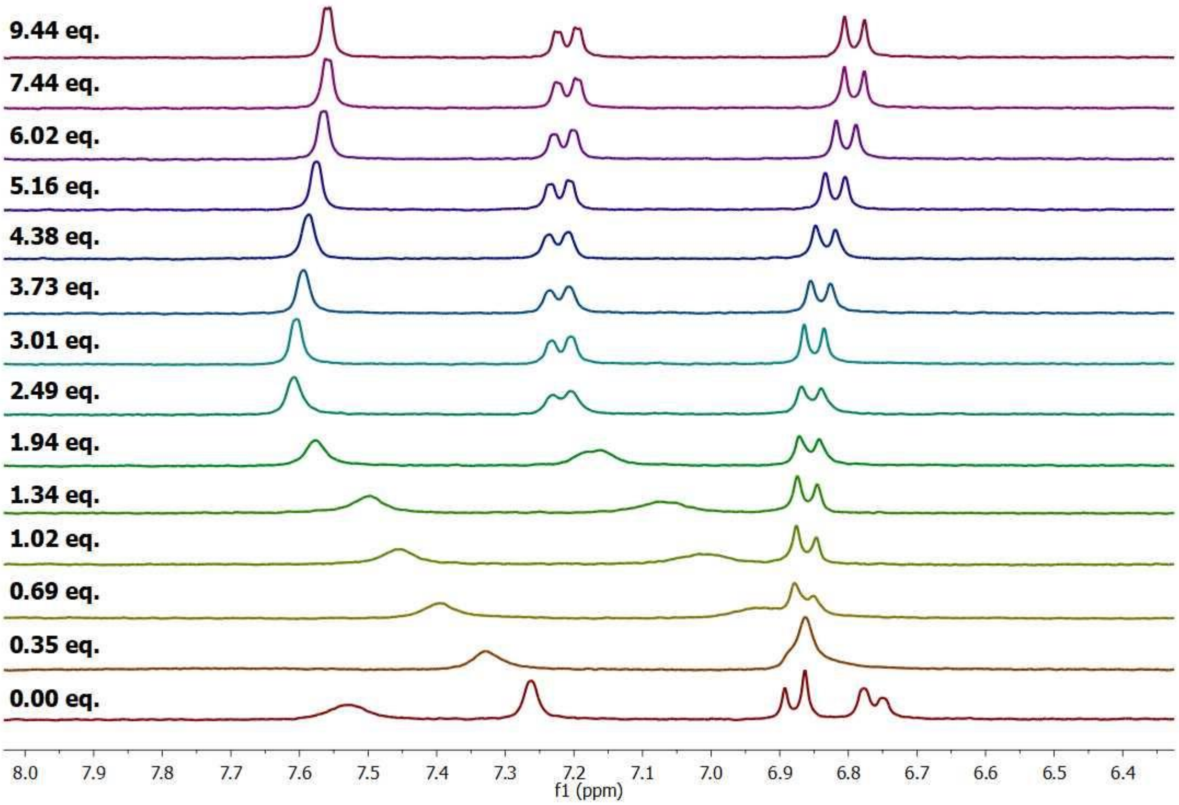

Fig. S49. ^{1}H NMR spectra recorded upon titration of receptor **3** in DMSO-d_6 with TBACl.

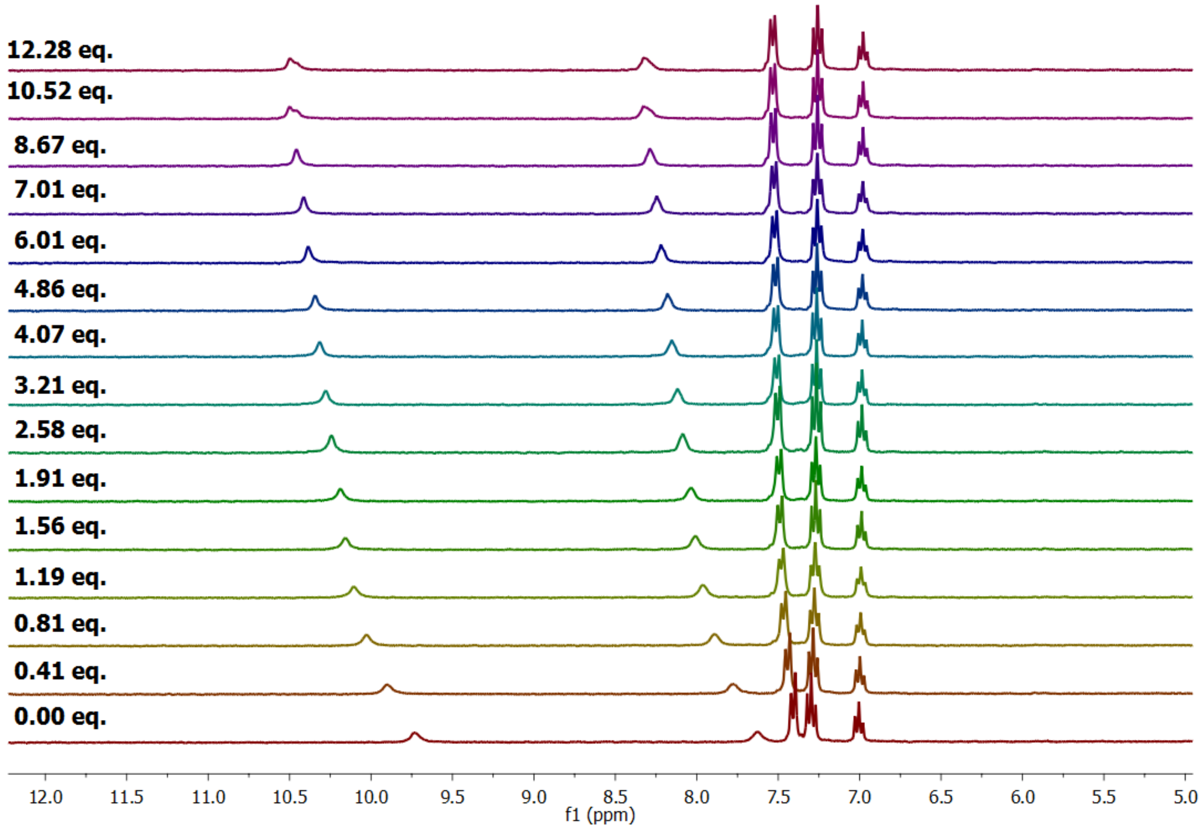

Fig. S50. ^{1}H NMR titration binding isotherms of receptor **3** in DMSO-d_6 upon addition of increasing amounts of TBACl.

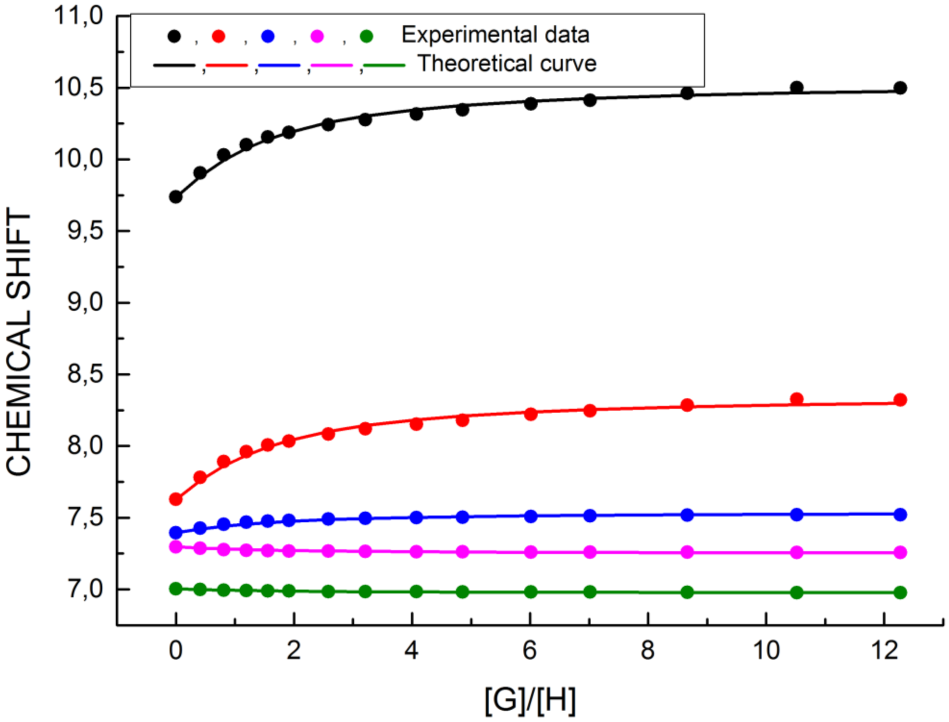

Fig. S51. ^{1}H NMR spectra recorded upon titration of receptor **3** in DMSO-d_6 with TBACl in the presence of 3 eq. $NaClO_4$.

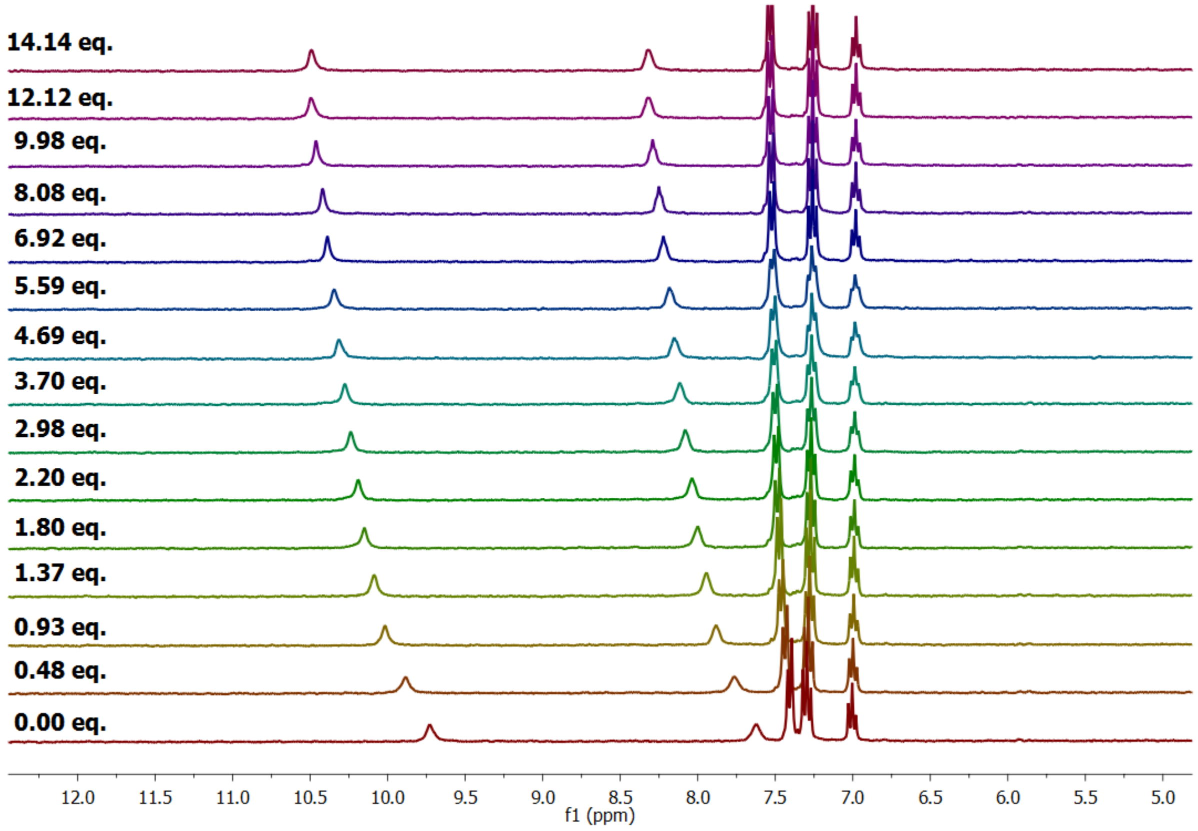

Fig. S52. ^{1}H NMR titration binding isotherms of receptor **3** in DMSO-d_6 upon addition of increasing amounts of TBACl the presence of 3 eq. $NaClO_4$.

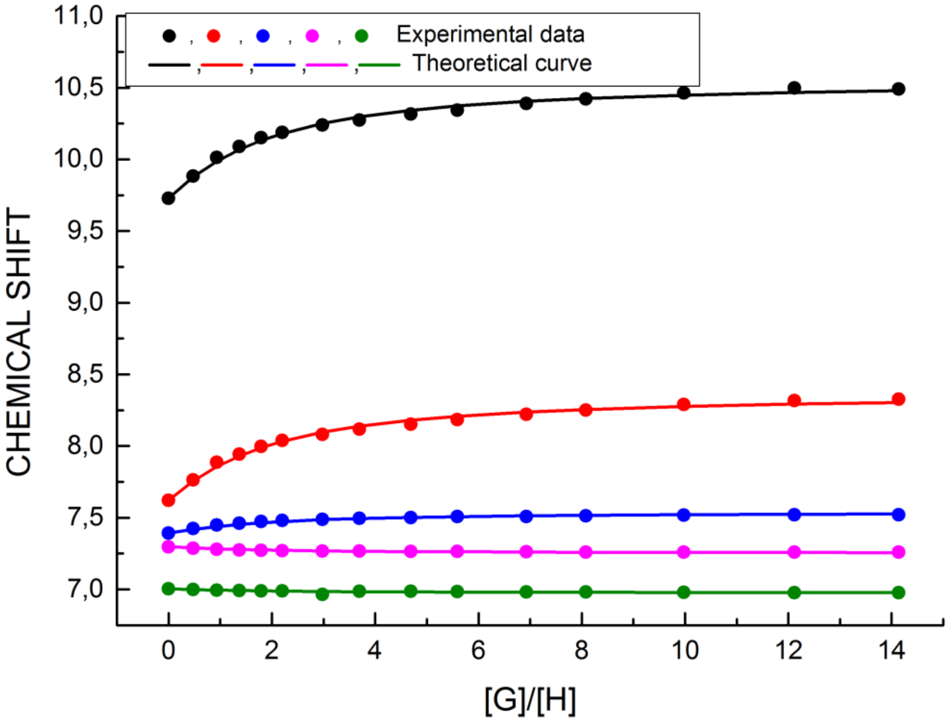

Fig. S53. ^{1}H NMR spectra recorded upon titration of receptor **3** in DMSO-d_6 with TBACl in the presence of 3 eq. KPF_6.

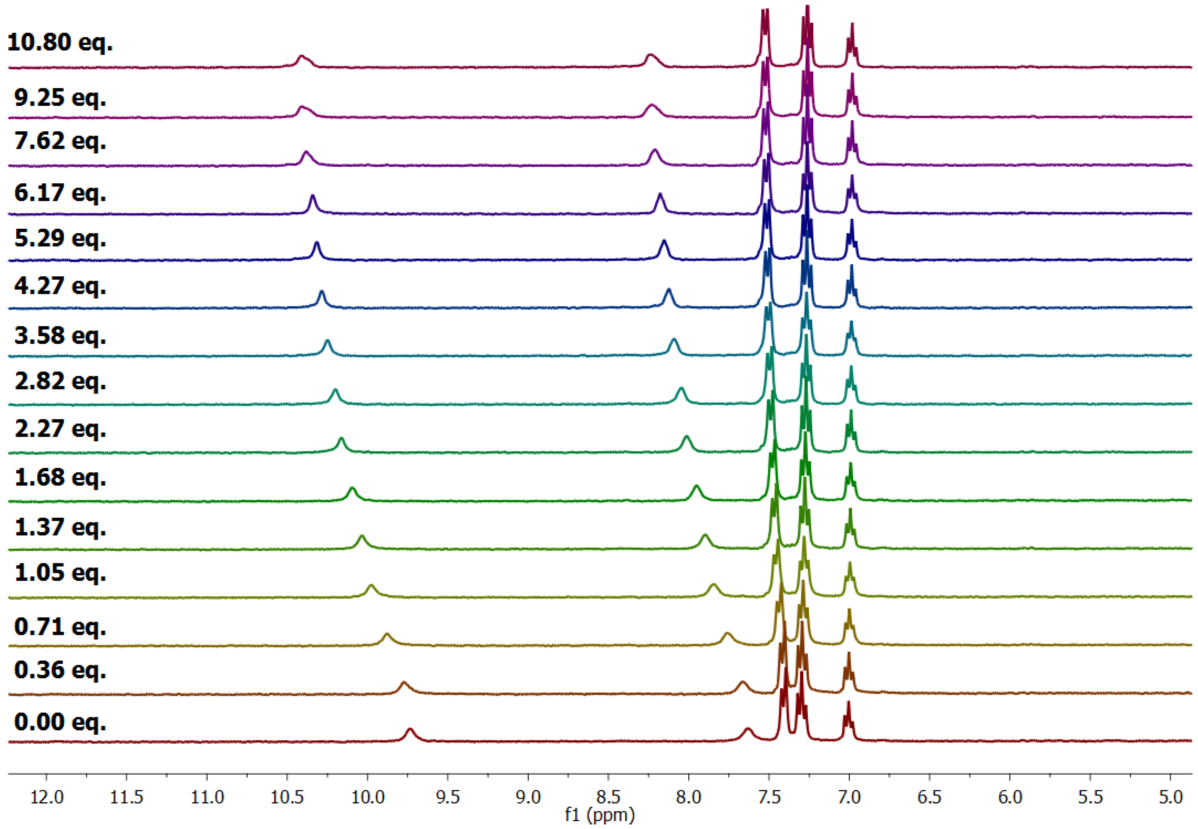

Fig. S54. ^{1}H NMR titration binding isotherms of receptor **3** in DMSO-d_6 upon addition of increasing amounts of TBACl the presence of 3 eq. KPF_6.

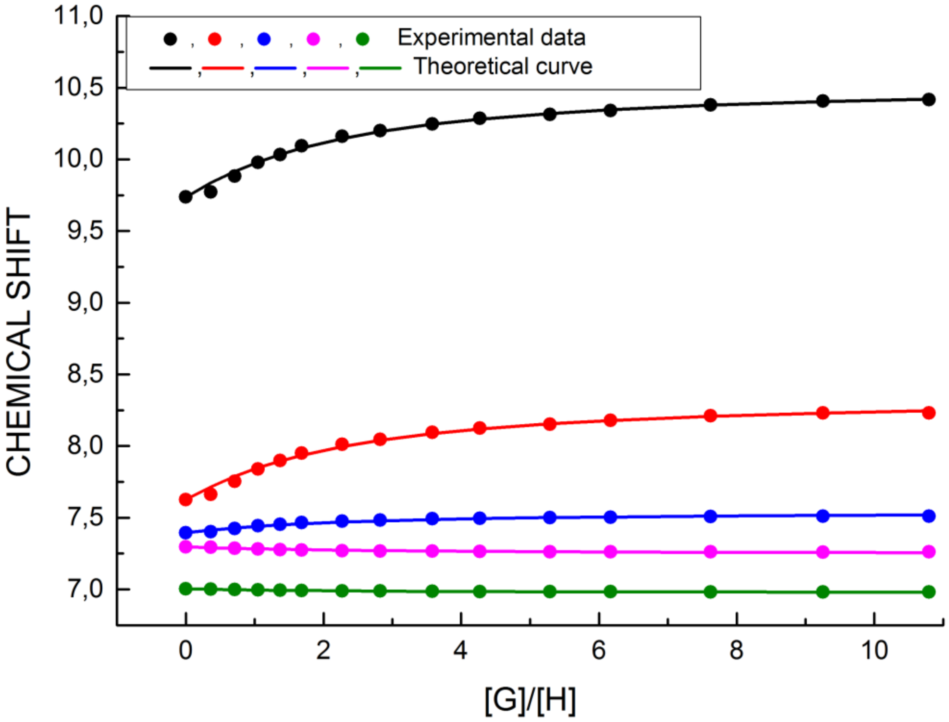

Fig. S55. ^{1}H NMR spectra recorded upon titration of receptor **4** in DMSO-d_6 with TBACl.

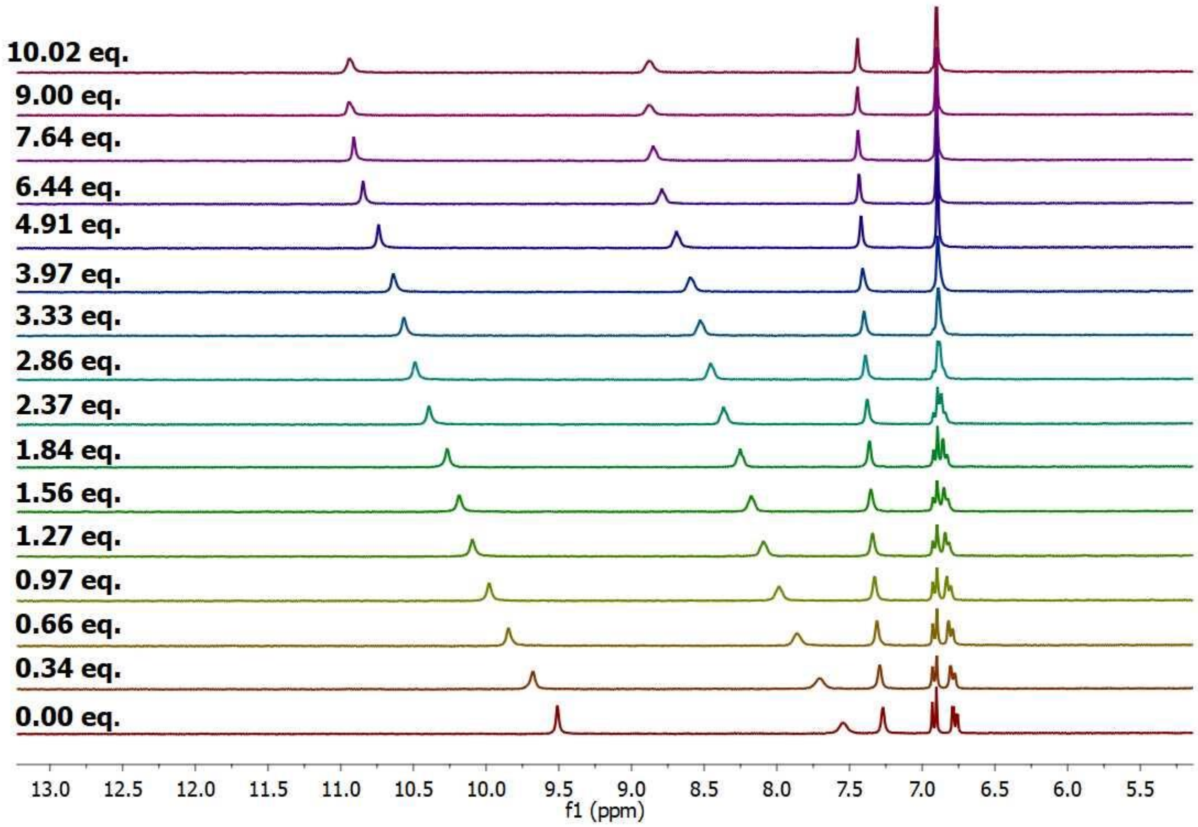

Fig. S56. ^{1}H NMR titration binding isotherms of receptor **4** in DMSO-d_6 upon addition of increasing amounts of TBACl.

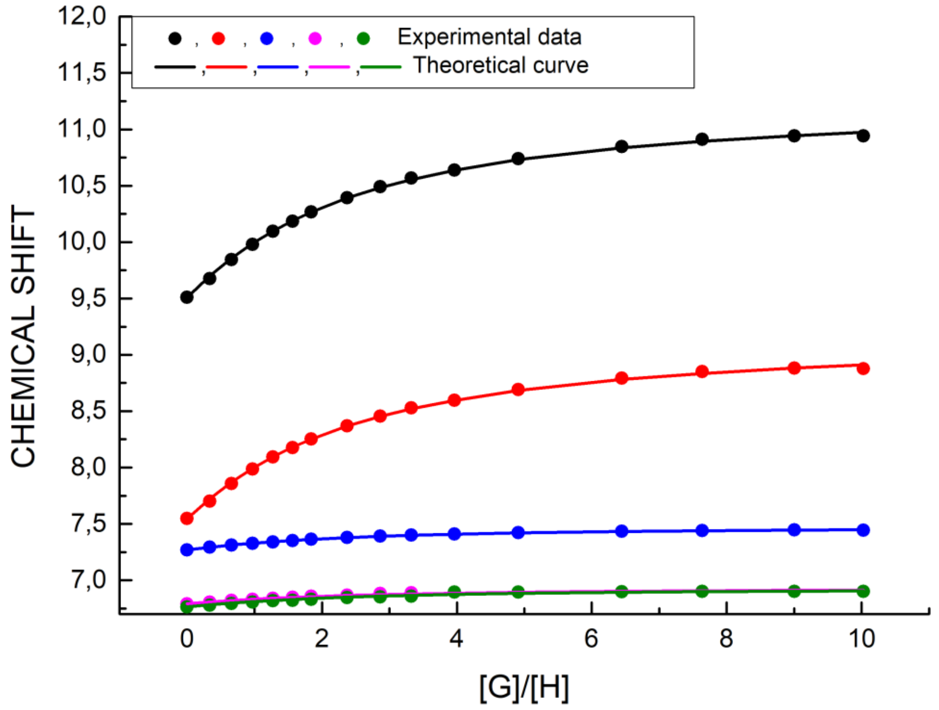

Fig. S57. ^{1}H NMR spectra recorded upon titration of receptor **4** in DMSO-d_6 with TBACl in the presence of 1 eq. $NaClO_4$.

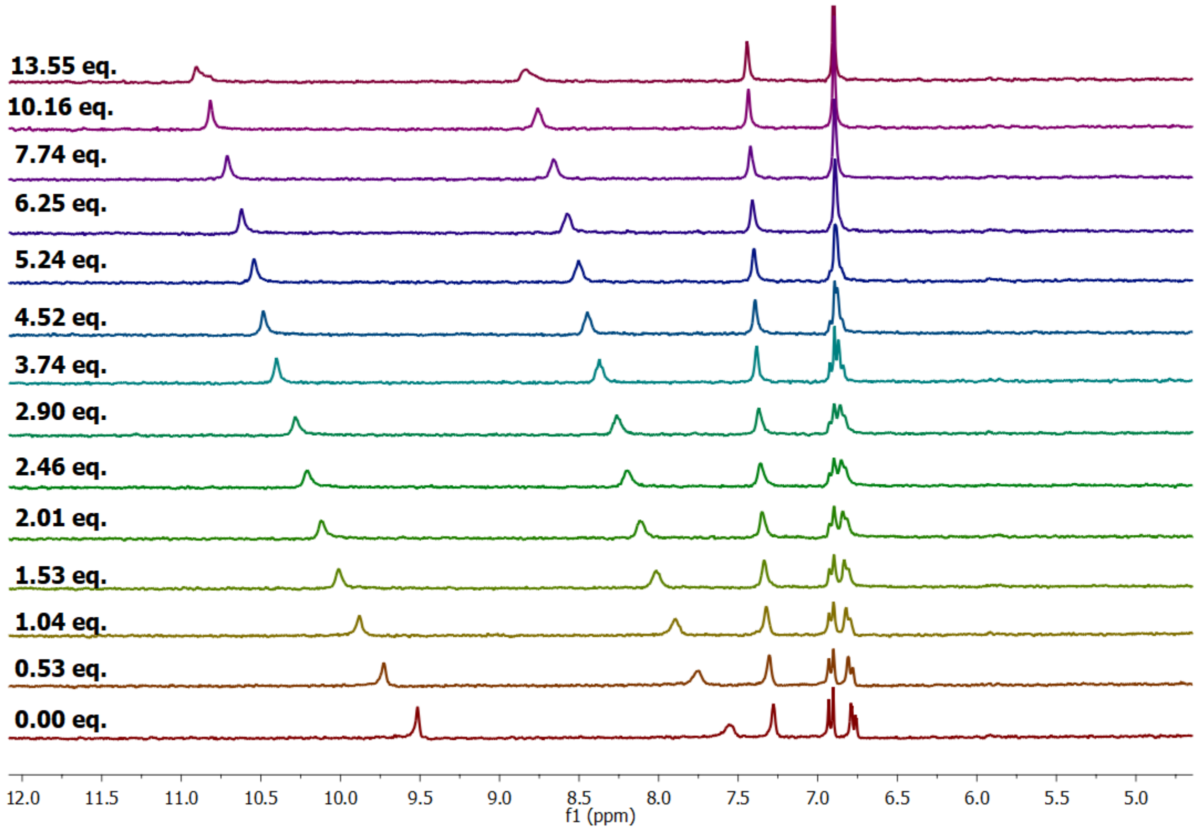

Fig. S58. ^{1}H NMR titration binding isotherms of receptor **4** in DMSO-d_6 upon addition of increasing amounts of TBACl the presence of 1 eq. $NaClO_4$.

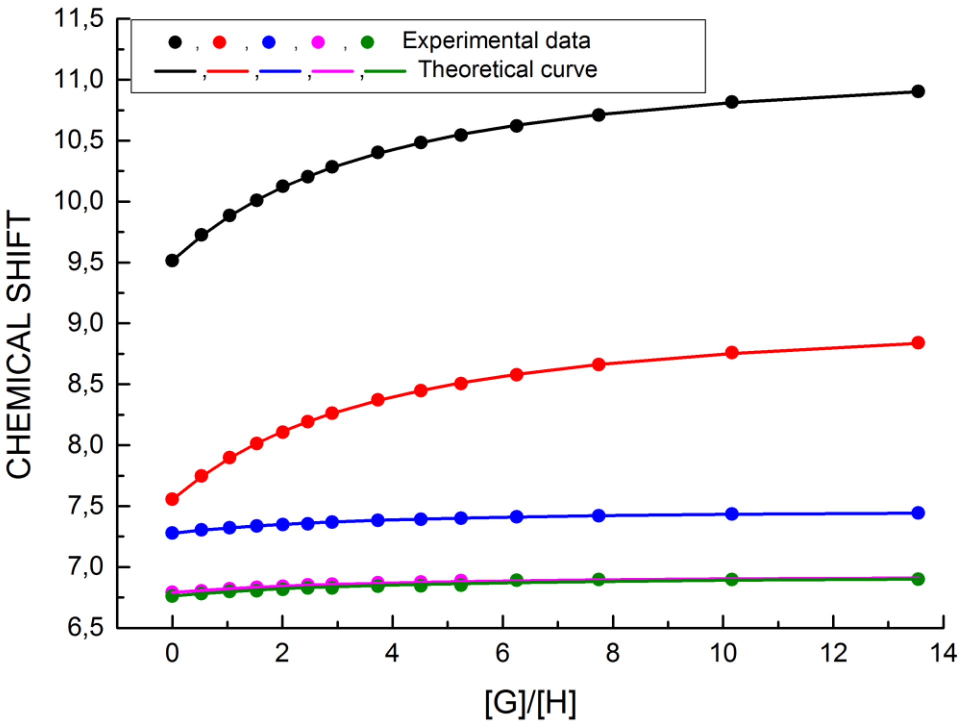

Fig. S59. ^{1}H NMR spectra recorded upon titration of receptor **4** in DMSO-d_6 with TBACl in the presence of 1 eq. KPF_6.

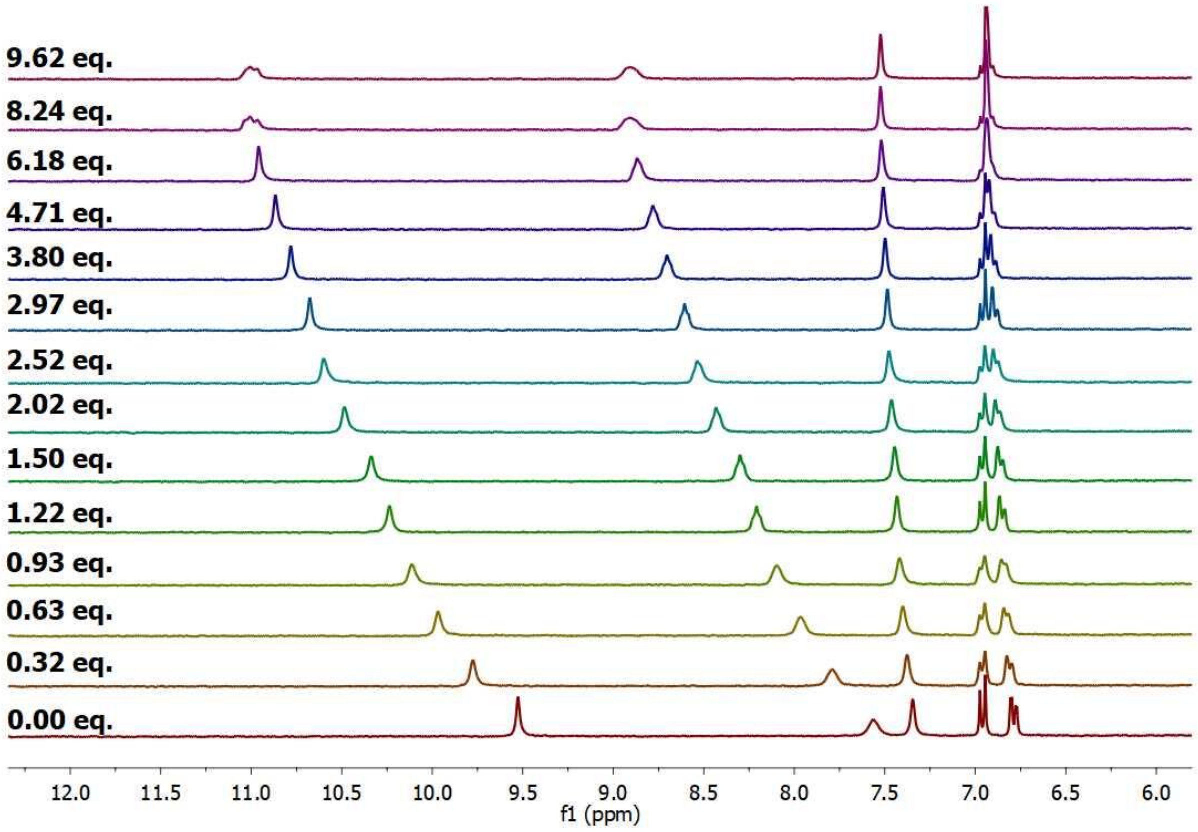

Fig. S60. ^{1}H NMR titration binding isotherms of receptor **4** in DMSO-d_6 upon addition of increasing amounts of TBACl the presence of 1 eq. KPF_6.

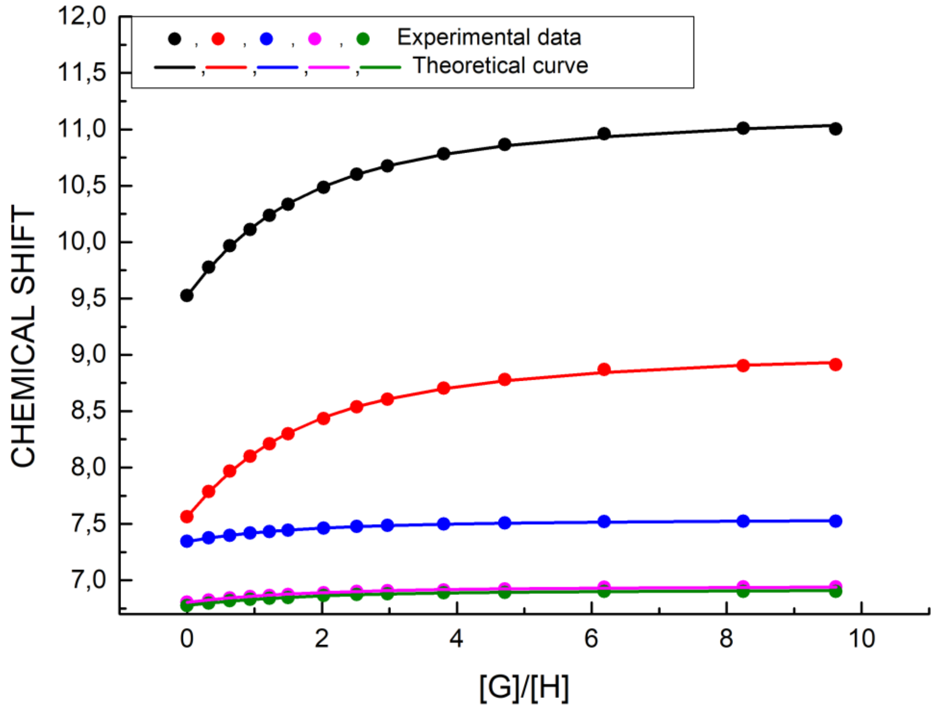

EXTRACTION EXPERIMENTS - ION CHROMATOGRAPHY

A solution of receptor **2** in chloroform (2 ml, ca. 5 mM) was intensive shaking with 5 mM aqueous solutions of suitable salts (2 ml) or mixture of salts or in the case of binary experiments 5 Mm sulfates and 25 Mm of other anions, for 5 minutes. In the case of pH dependent experiments $HClO_4$ was used to adjust pH of aqueous phase. Then 1 mL of aqueous phase was taken and tenfold diluted. The concentration of chloride, bromide, nitrite, nitrate, phosphate and sulfate anions in aqueous phase was determined by high performance ion chromatography (HPIC).

Fig. S61: Chromatograms obtained during extraction experiments after tenfold dilution (a) source phase: 5 Mm KCl solution (b) after extraction with 5 mM of **2** in $CHCl_3$.

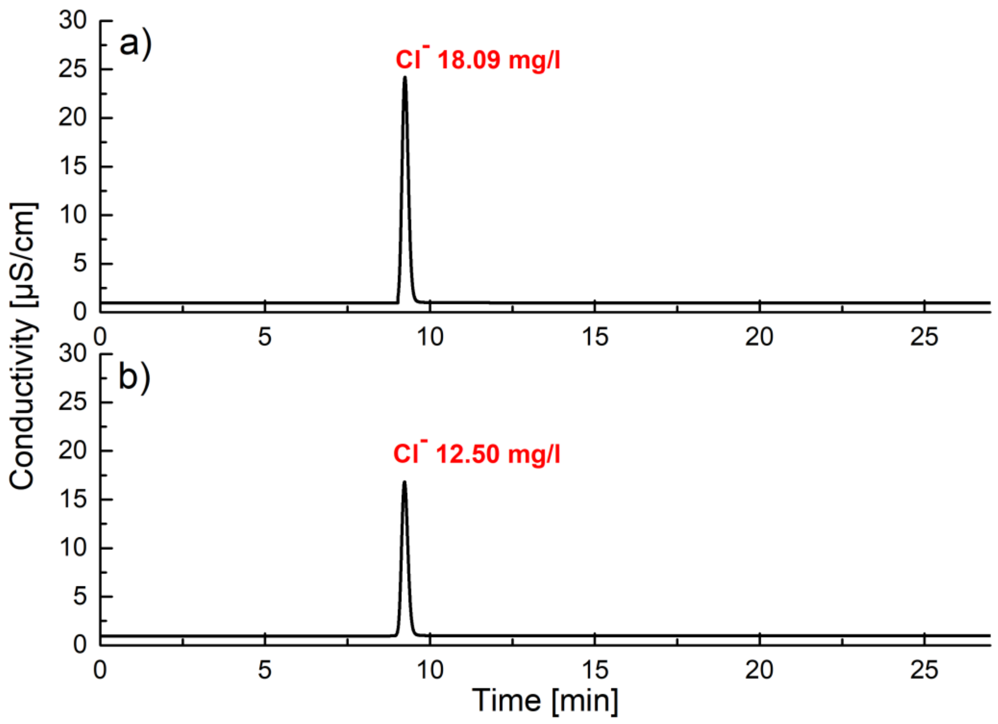

Fig. S62: Chromatograms obtained during extraction experiments after tenfold dilution (a) source phase: 5 Mm KBr solution (b) after extraction with 5 mM of **2** in $CHCl_3$.

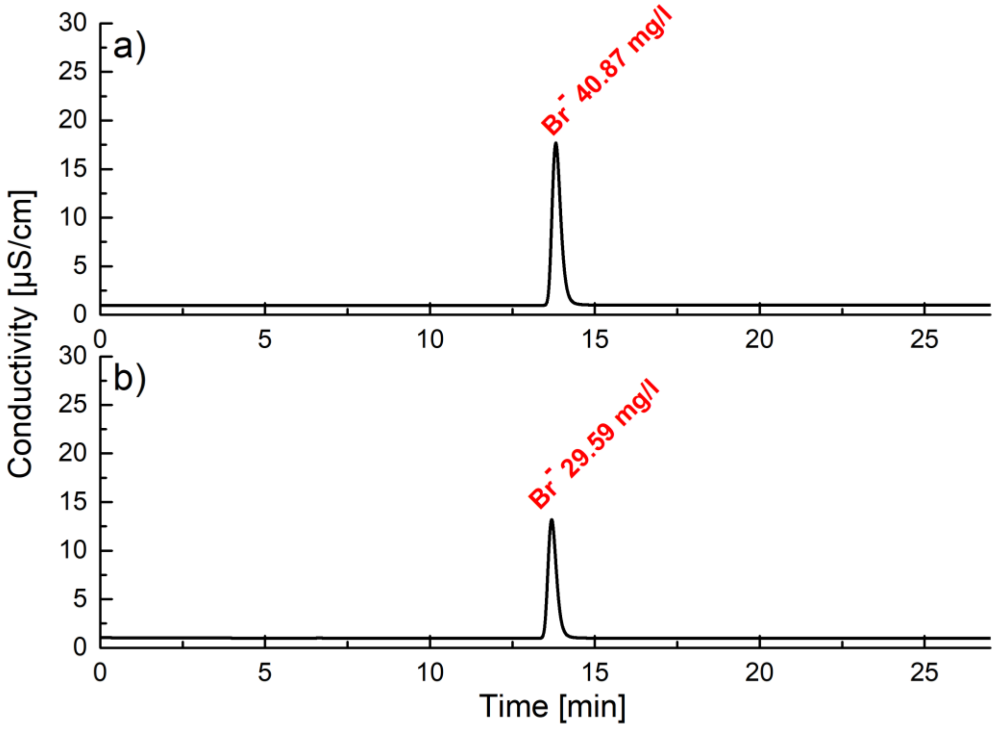

Fig. S63: Chromatograms obtained during extraction experiments after tenfold dilution (a) source phase: 5 Mm KNO_3 solution (b) after extraction with 5 mM of **2** in $CHCl_3$.

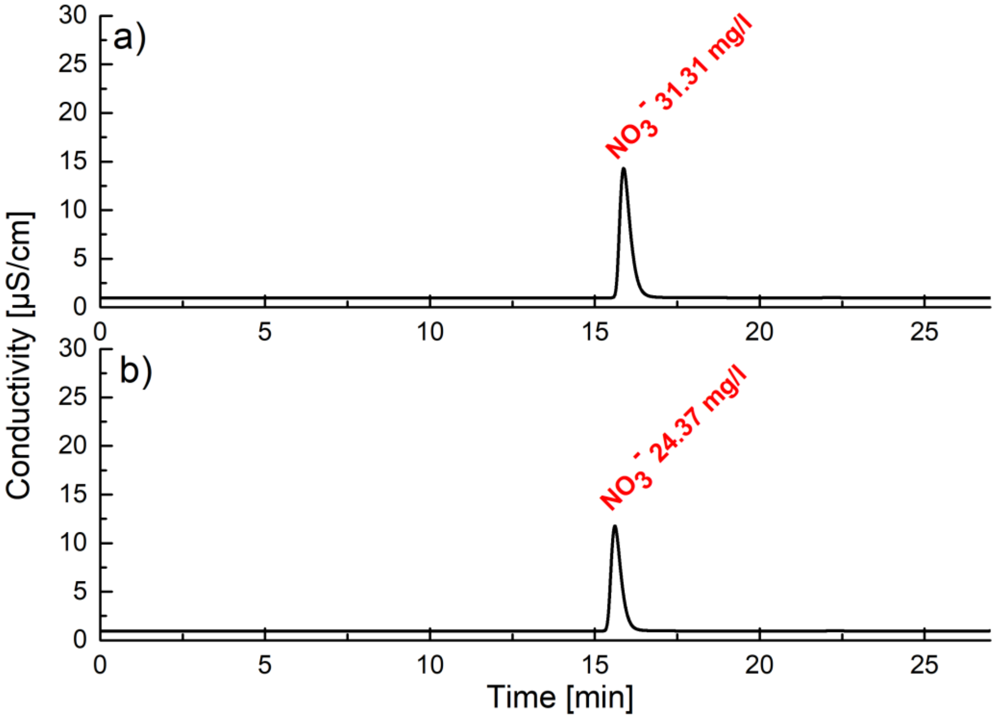

Fig. S64: Chromatograms obtained during extraction experiments after tenfold dilution (a) source phase: 5 Mm KNO_2 solution (b) after extraction with 5 mM of **2** in $CHCl_3$.

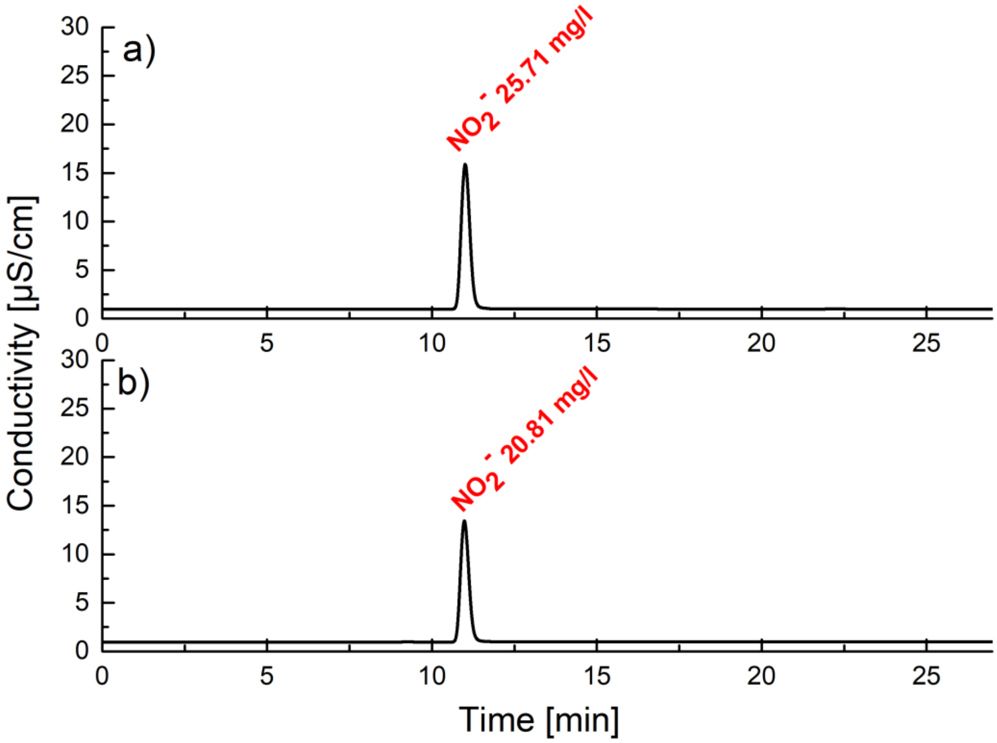

Fig. S65: Chromatograms obtained during extraction experiments after tenfold dilution (a) source phase: 5 Mm K_2SO_4 solution (b) after extraction with 5 mM of **2** in $CHCl_3$.

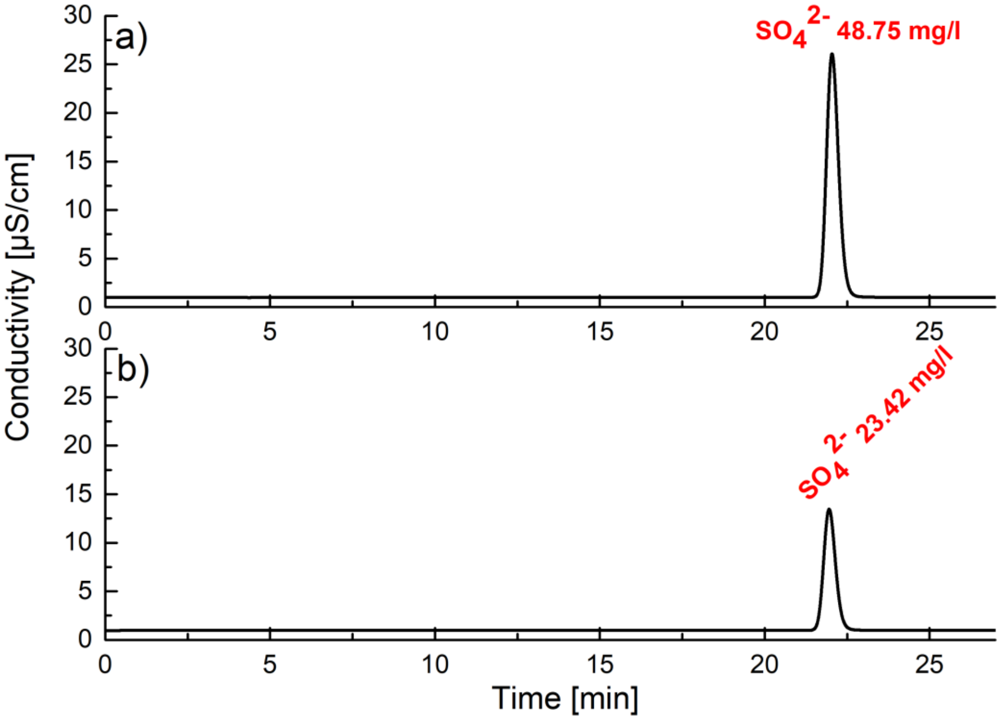

Fig. S66: Chromatograms obtained during extraction experiments after tenfold dilution (a) source phase: 5 Mm KH_2PO_4 solution (b) after extraction with 5 mM of **2** in $CHCl_3$.

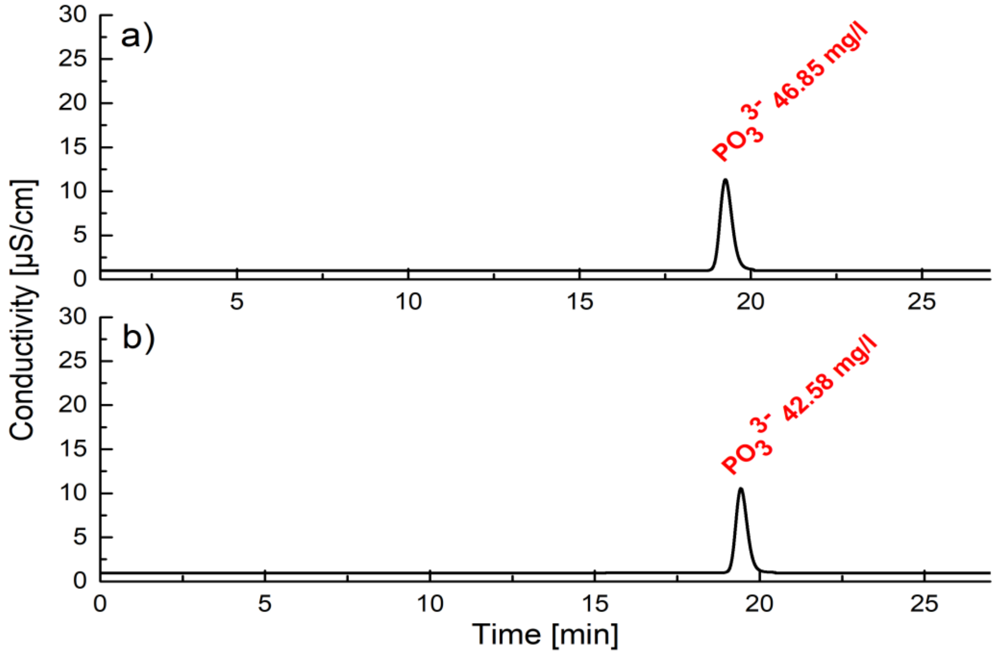

Fig. S67: Chromatograms obtained during extraction experiments with pH = 5 after tenfold dilution (a) source phase: 4.22 Mm K_2SO_4 solution (b) after extraction with 5 mM of **2** in $CHCl_3$.

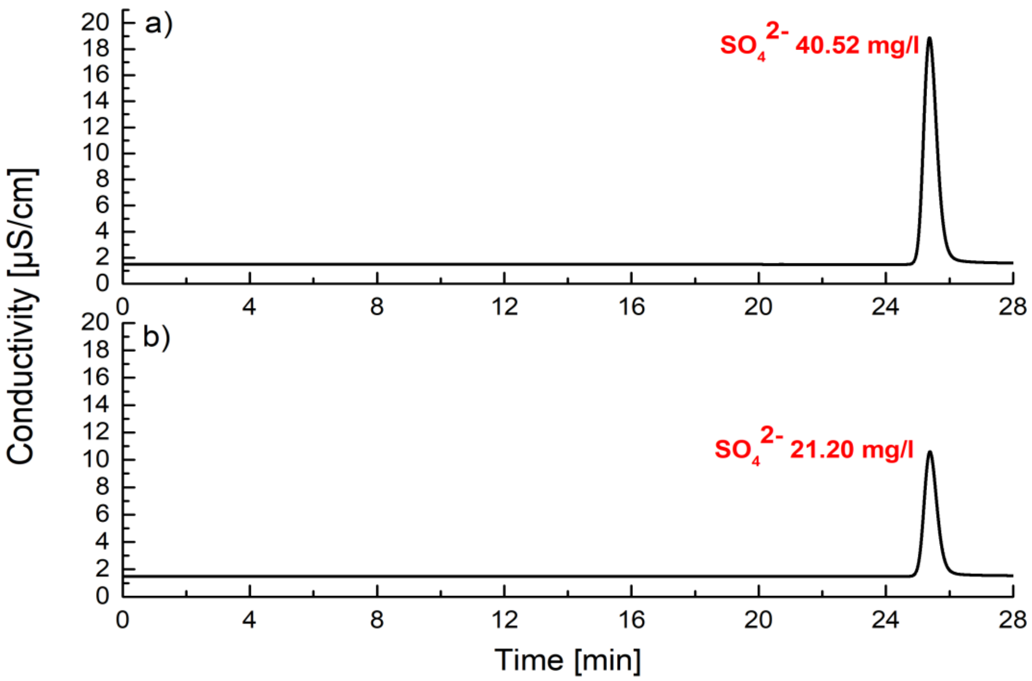

Fig. S68: Chromatograms obtained during extraction experiments with pH = 4 after tenfold dilution (a) source phase: 3.93 Mm K_2SO_4 solution (b) after extraction with 5 mM of **2** in $CHCl_3$.

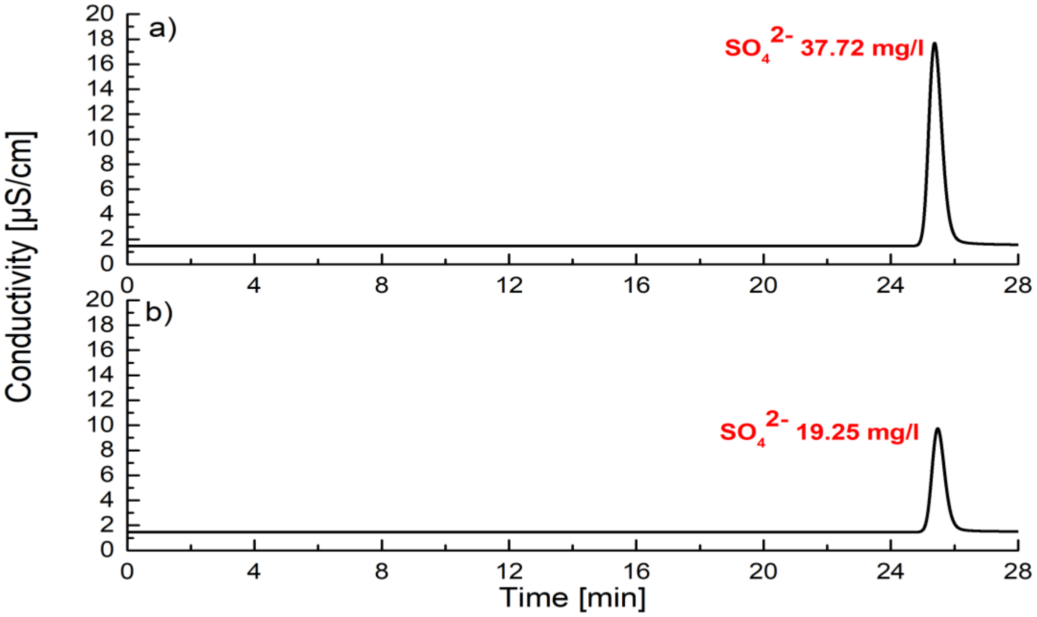

Figure 69. Chromatograms obtained during extraction with pH=4 experiment after tenfold dilution: (a) source phase: mixture of 5 Mm anions; (b) after extraction with 5 mM **2** in $CHCl_3$.

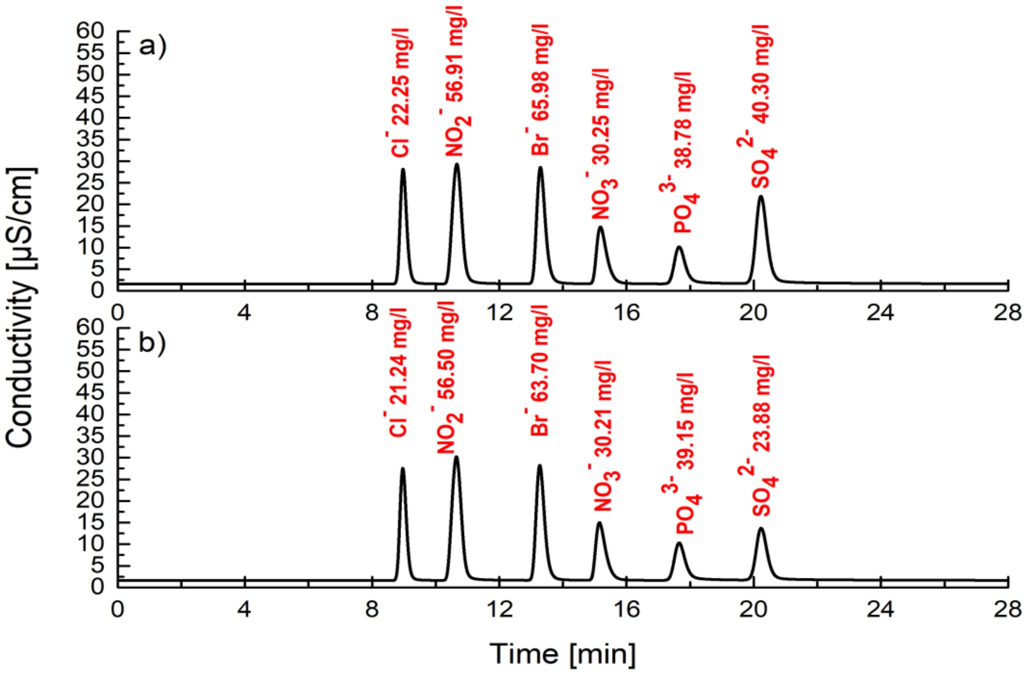

Figure 70. Chromatograms obtained during extraction after tenfold dilution: (a) source phase: mixture of KCl (25mM) and K_2SO_4 (5mM); (b) after extraction with 5.15 mM **2** in $CHCl_3$.

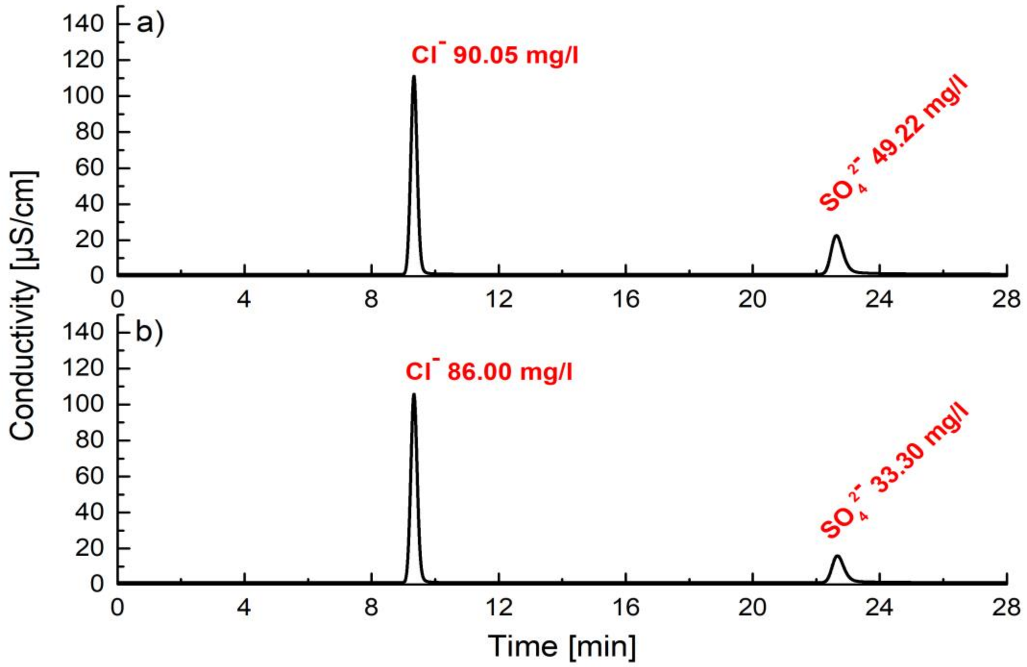

Figure 71. Chromatograms obtained during extraction after tenfold dilution: (a) source phase: mixture of KBr (25mM) and K_2SO_4 (5mM); (b) after extraction with 5.15 mM **2** in $CHCl_3$.

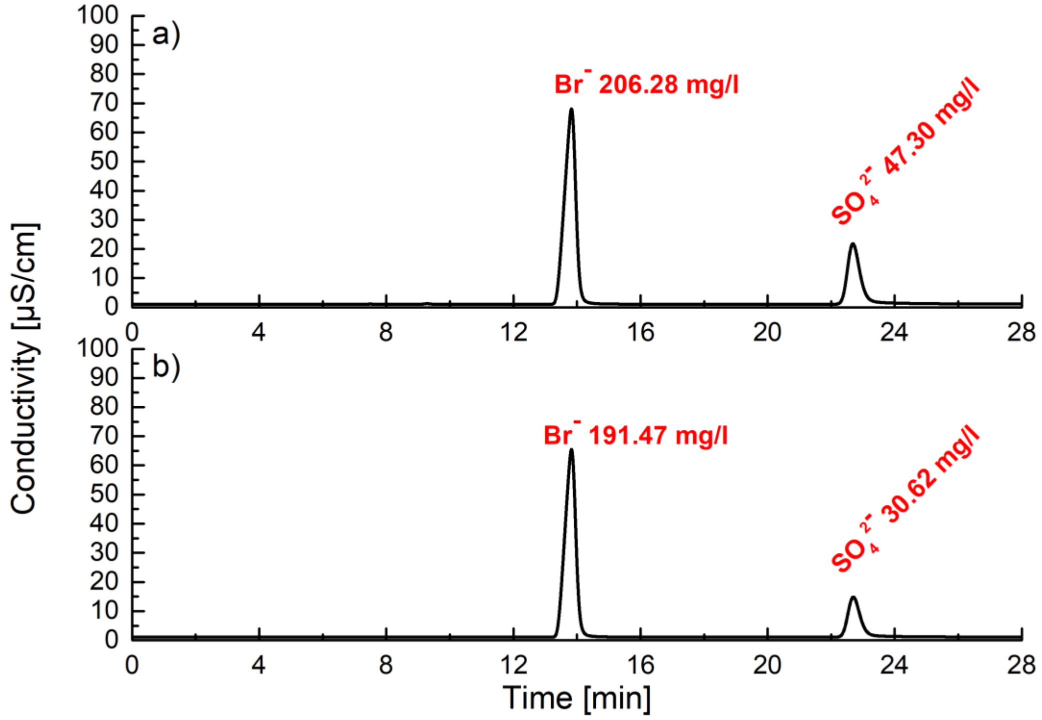

Figure 72. Chromatograms obtained during extraction after tenfold dilution: (a) source phase: mixture of KNO_2 (25mM) and K_2SO_4 (5mM); (b) after extraction with 5.15 mM **2** in $CHCl_3$.

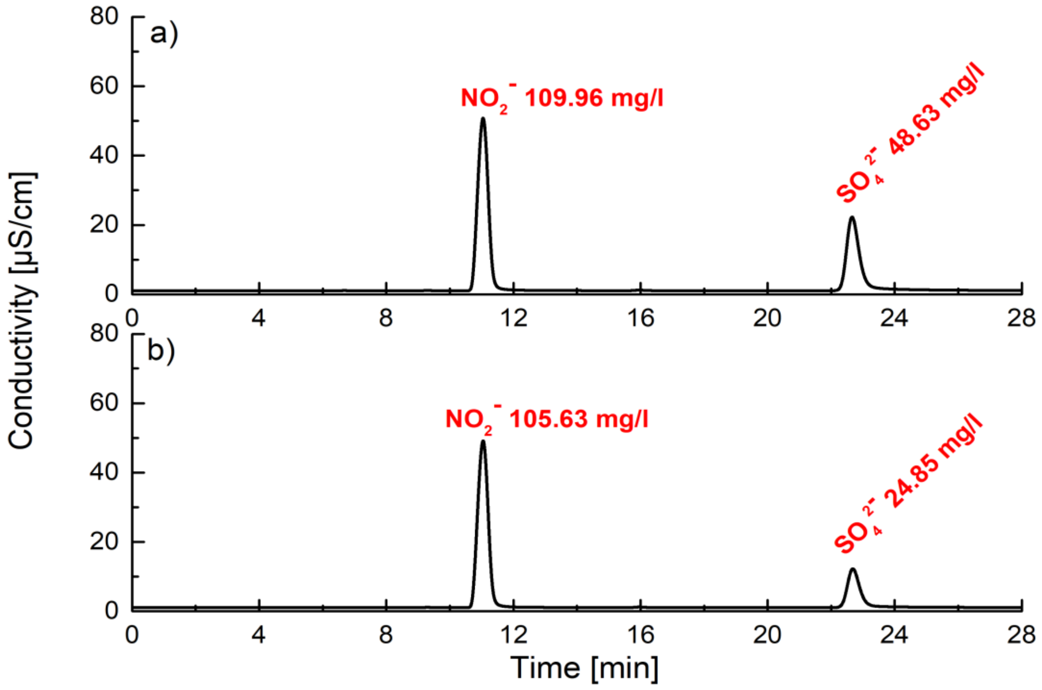

Figure 73. Chromatograms obtained during extraction after tenfold dilution: (a) source phase: mixture of KNO_3 (25mM) and K_2SO_4 (5mM); (b) after extraction with 5.15 mM **2** in $CHCl_3$.

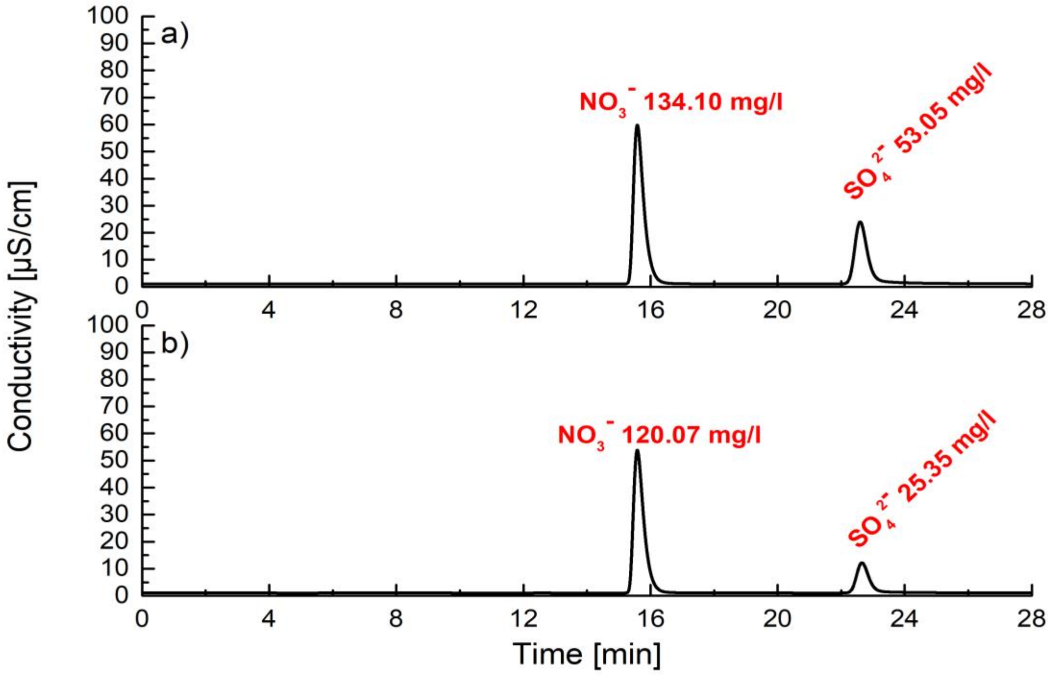

Figure 74. Chromatograms obtained during extraction after tenfold dilution: (a) source phase: mixture of KH_2PO_4 (25mM) and K_2SO_4 (5mM); (b) after extraction with 5.15 mM **2** in $CHCl_3$.

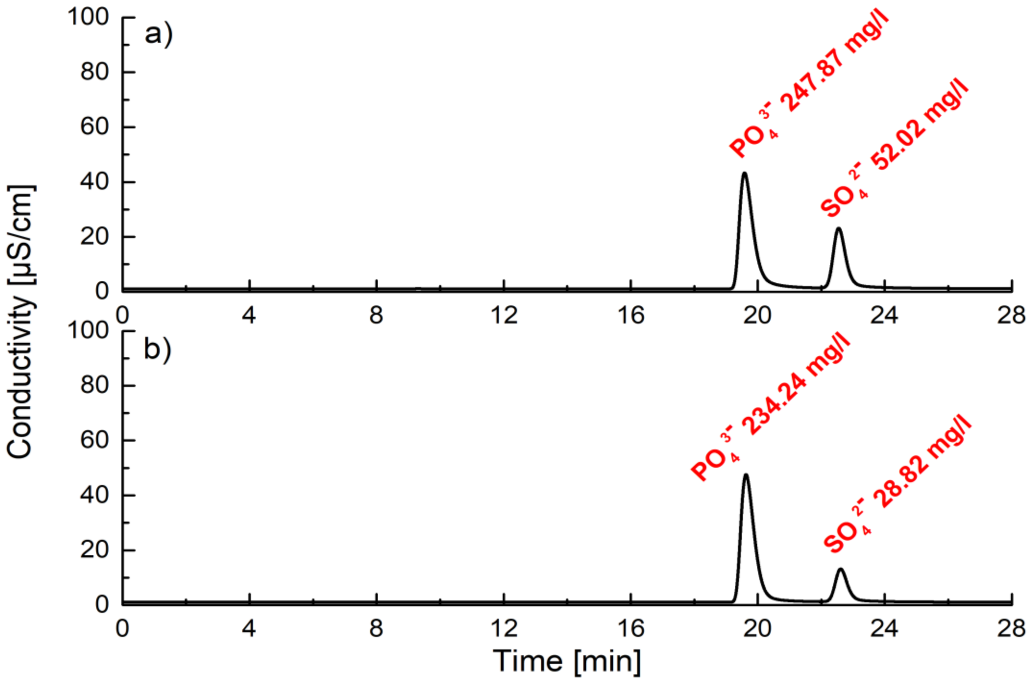

MS EXPERIMENTS

Fig. S75: Mass spectra recorded for organic phase after extraction of 5 Mm aqueous solution of KCl, KBr and K_2SO_4 with 5 mM solution of **2** in $CHCl_3$.

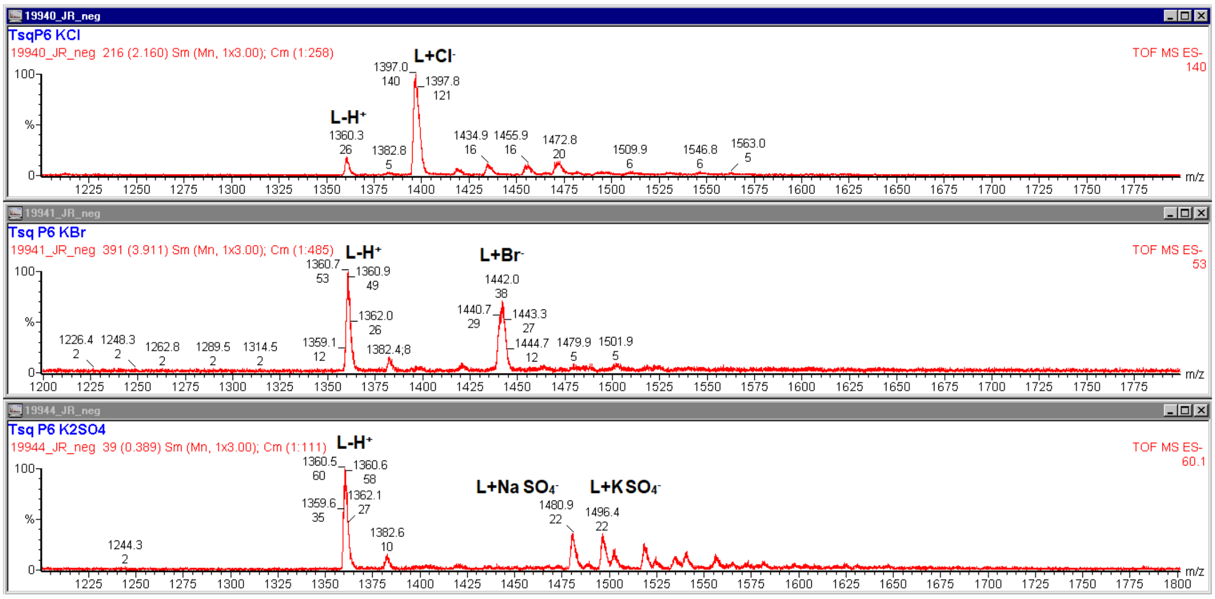

^{1}H NMR DOSY, ROESY AND HSQC EXPERIMENTS

Fig. S76: Diffusion coefficients analysis of water (a) and the unknown signal at 9,63 ppm (b) in the system containing **2** (1.5 mM) and addition 5 equivalents of TBA_2SO_4 in DMSO.

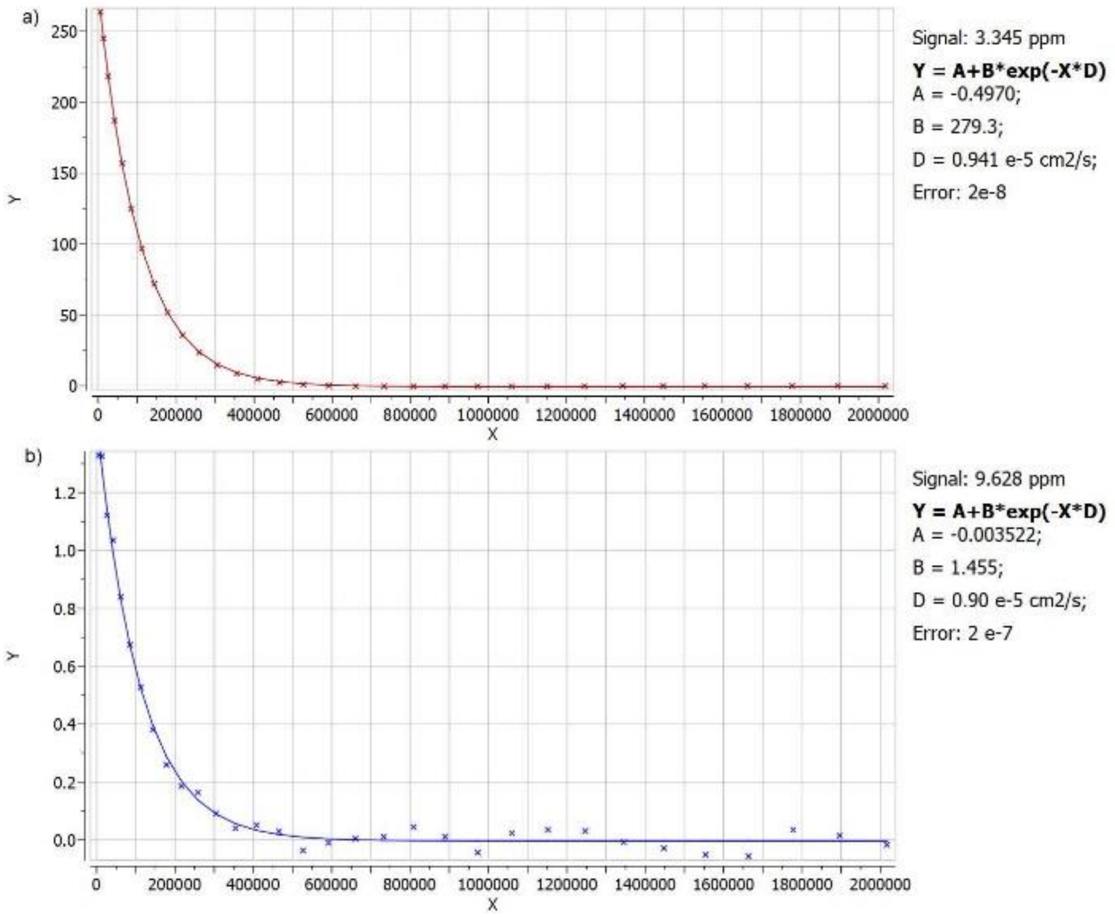

Fig. S77: ROESY spectrum of **2** with 5 equivalents of TBA_2SO_4 in DMSO.

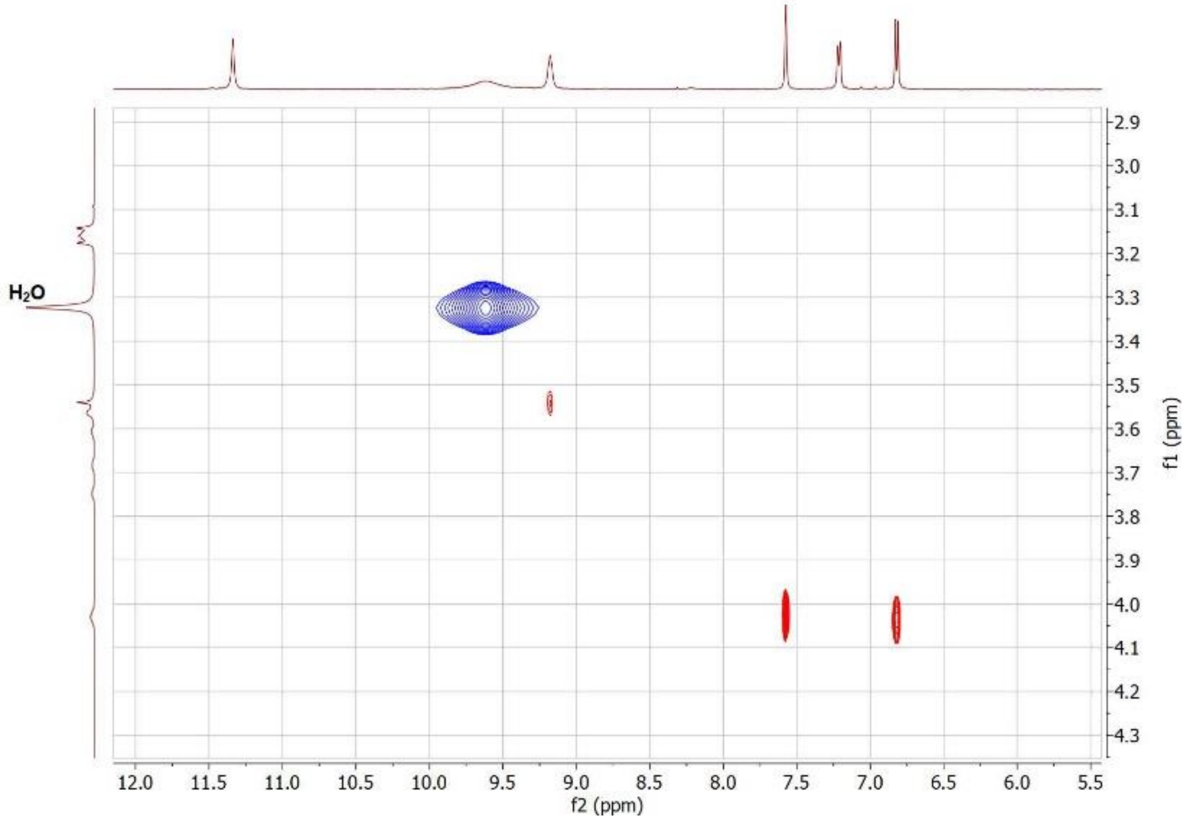

Fig. S78: HSQC spectrum of **2** with 5 equivalents of TBA_2SO_4 in DMSO. The signal near 9.63 ppm is not coupled to any carbon.

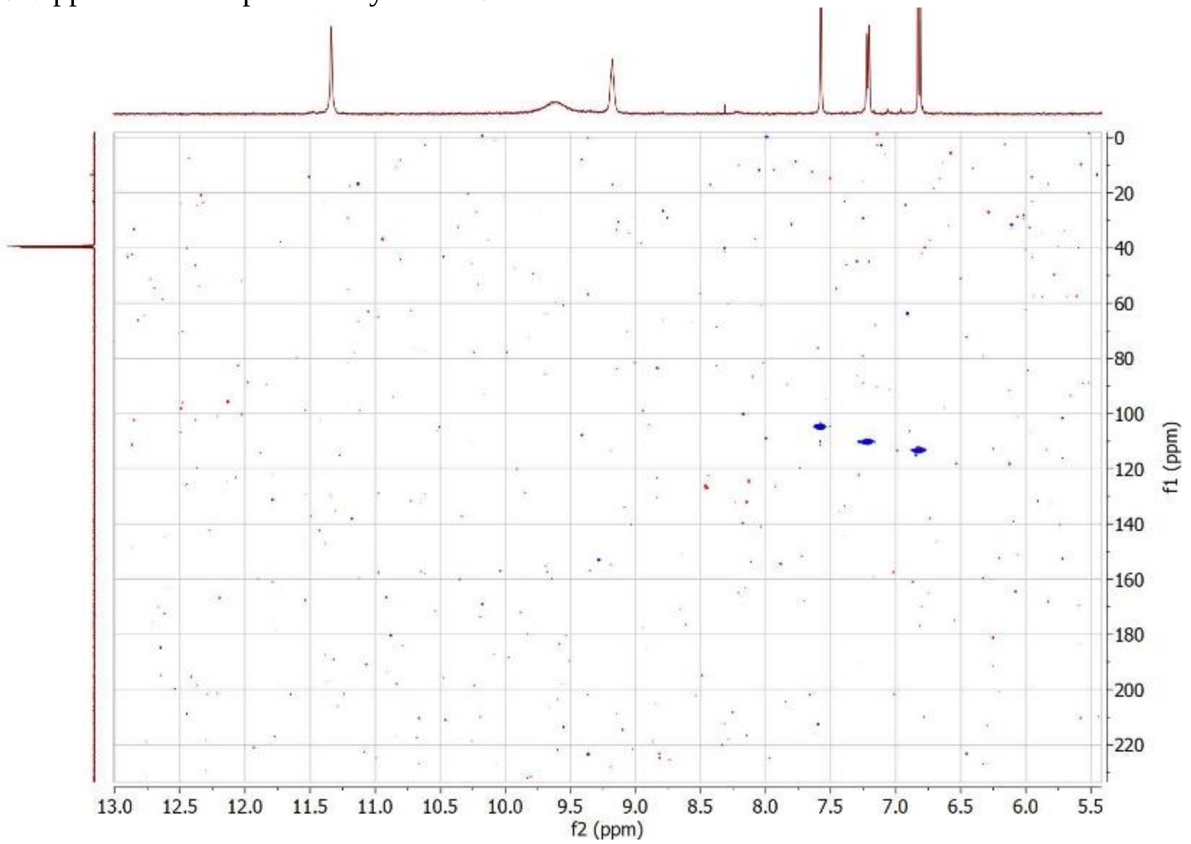

Fig. S79: DOSY spectrum of **2** with 3 eq. of KPF_6 and 1 eq. of TBACl in DMSO.

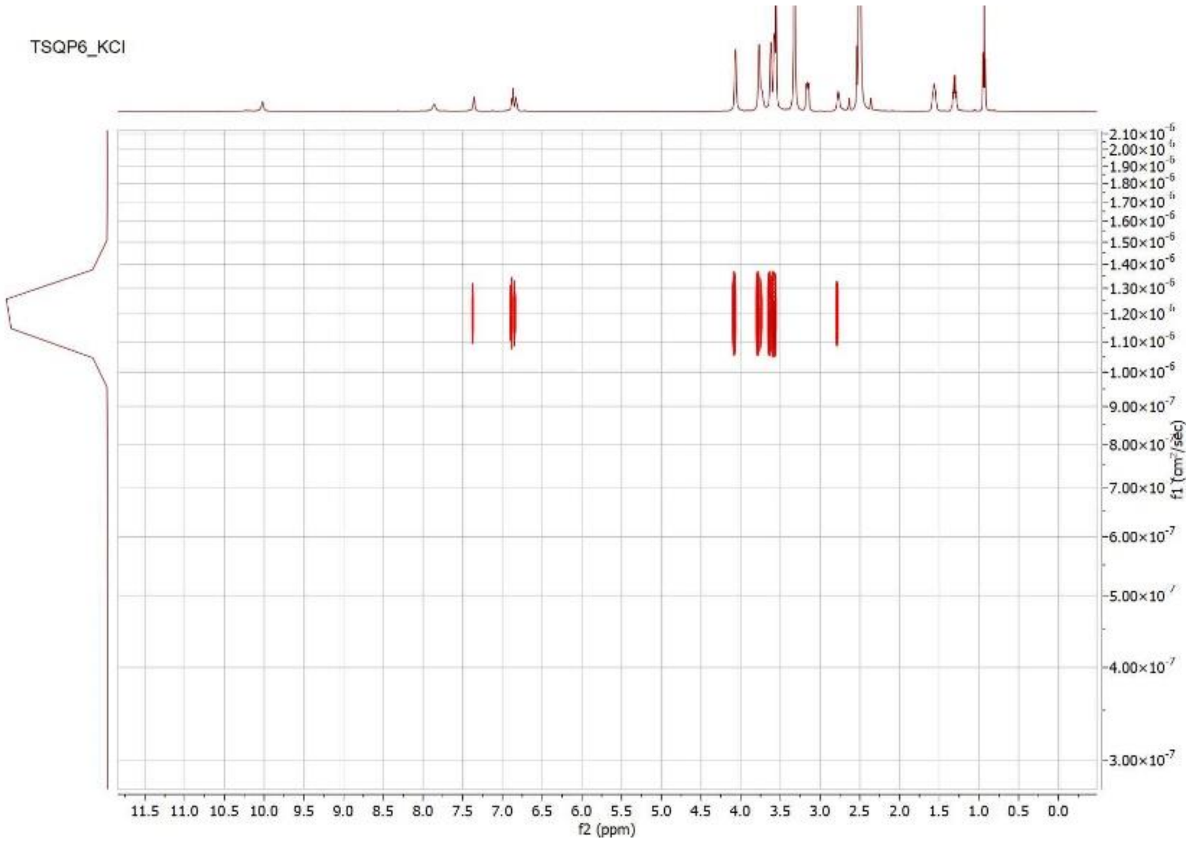

Fig. S80: DOSY spectrum of **2** with 3 eq. of KPF_6 and 1 eq. of TBA_2SO_4 in DMSO.

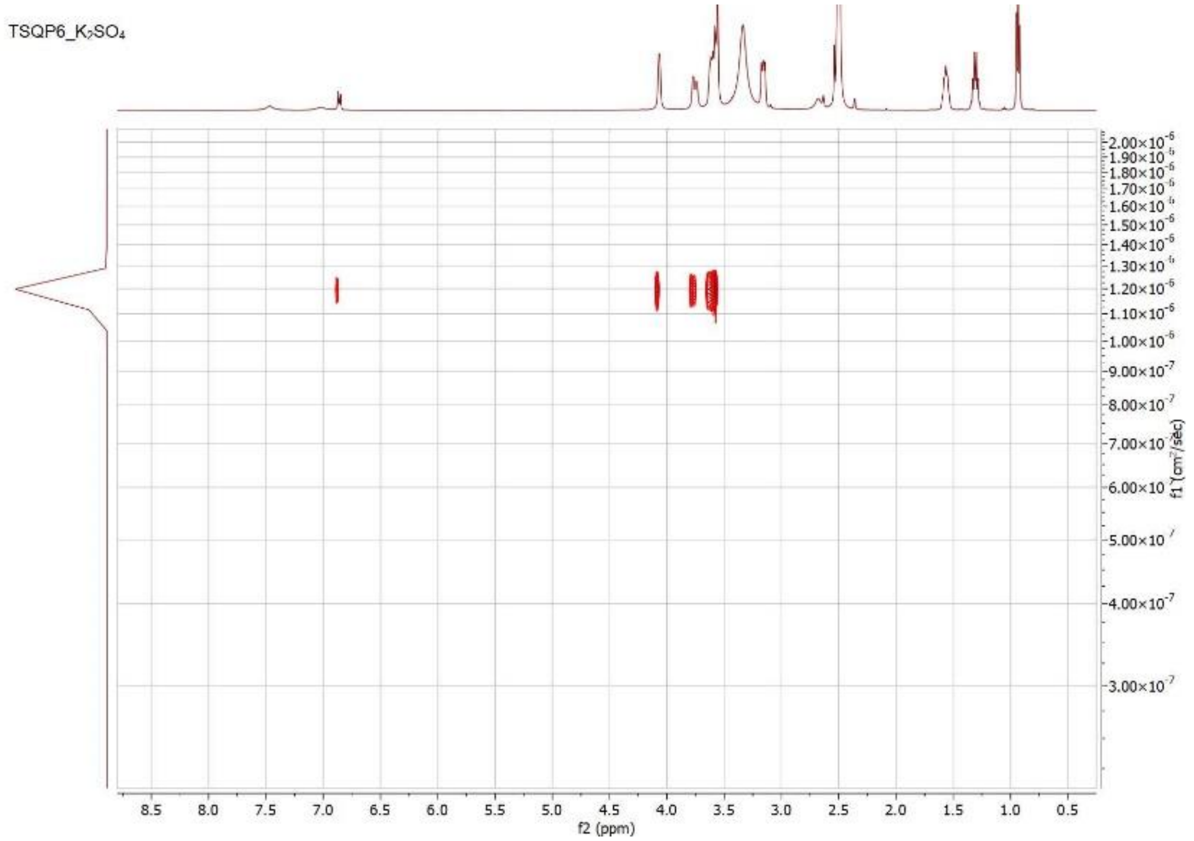

Fig. S81: DOSY spectrum of **2** (1 mM, $CDCl_3$) and TMS in wet $CDCl_3$.

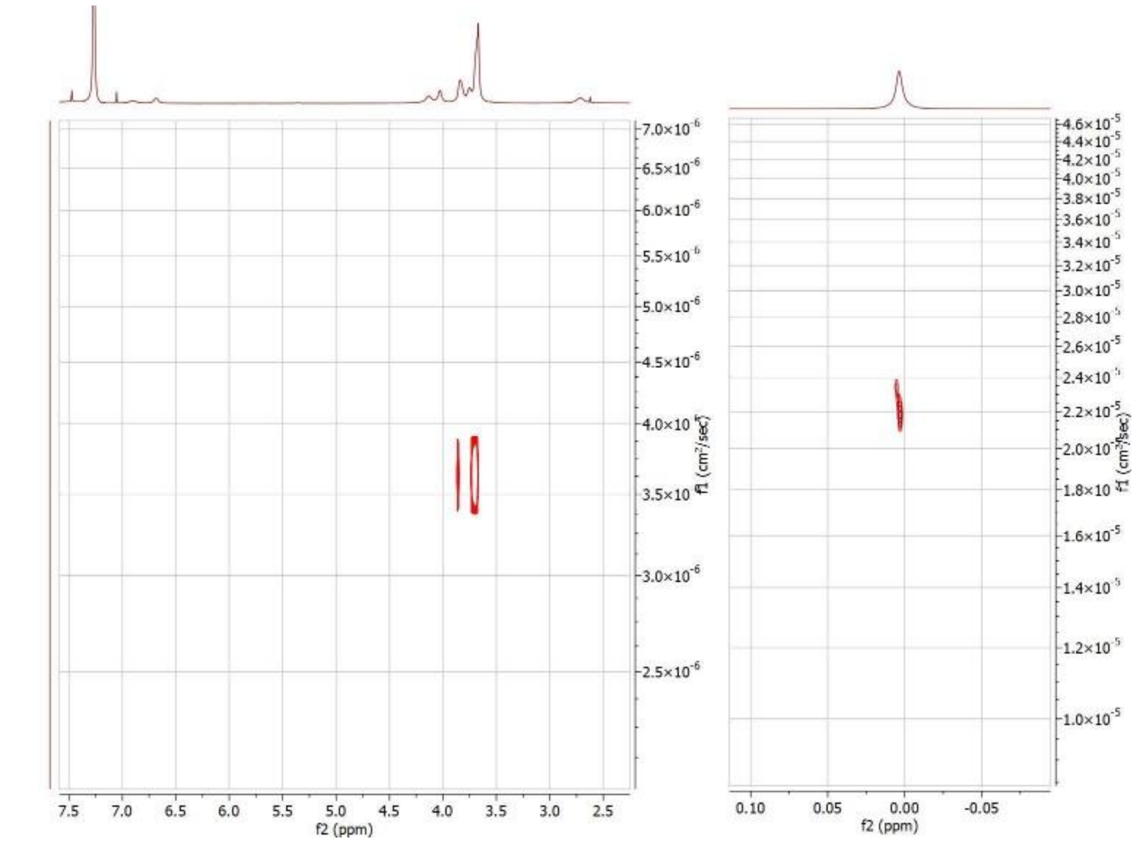

Fig. S82: DOSY spectrum of **2** (1 mM) upon extraction of aq. K_2SO_4 in wet $CDCl_3$.

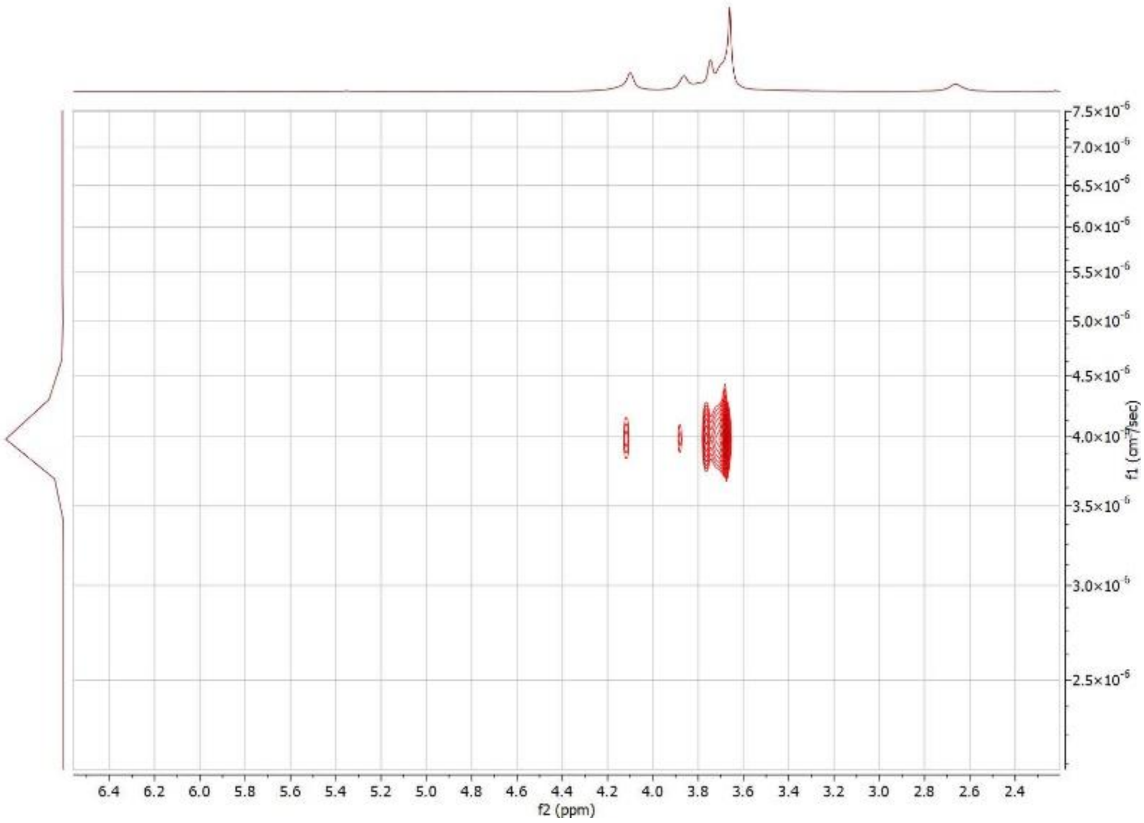

Fig. S83: DOSY spectrum of **2** (1 mM) upon extraction of aq. KCl in wet $CDCl_3$.

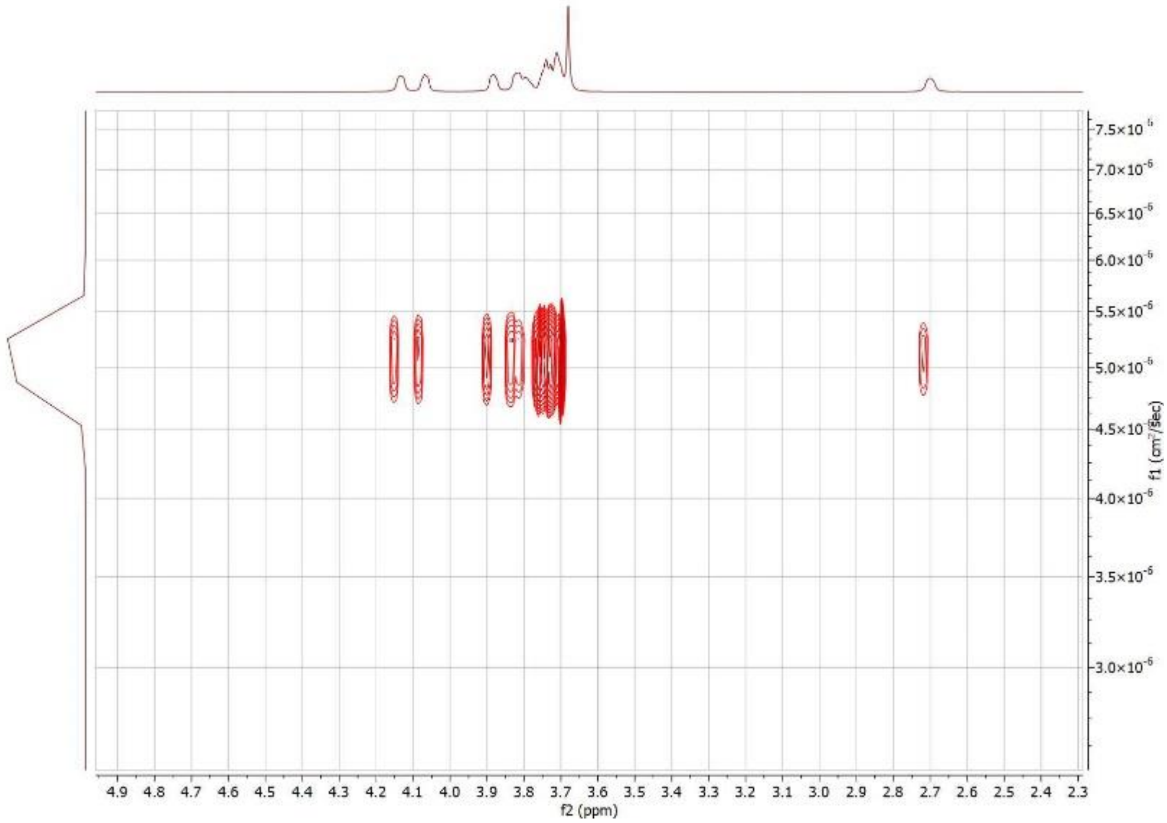

MOLECULAR MODELLING

Molecular modelling of receptor **2** was performed using Spartan 10 for Windows (Wavefunction, Inc. Irvine, CA). Firstly the structure was energy minimized using molecular mechanics and in the case of **2**·KCl complex, after that a Cl^- was placed near to one of anion binding domain and K^+ was placed in one of the crown ether cavity, then was optimized by density functional theory (DFT) calculations at the B3LYP/6-31G* level of theory.

Fig. S84: DFT calculated structure of the **2**, with E = - 4755.6614 au.

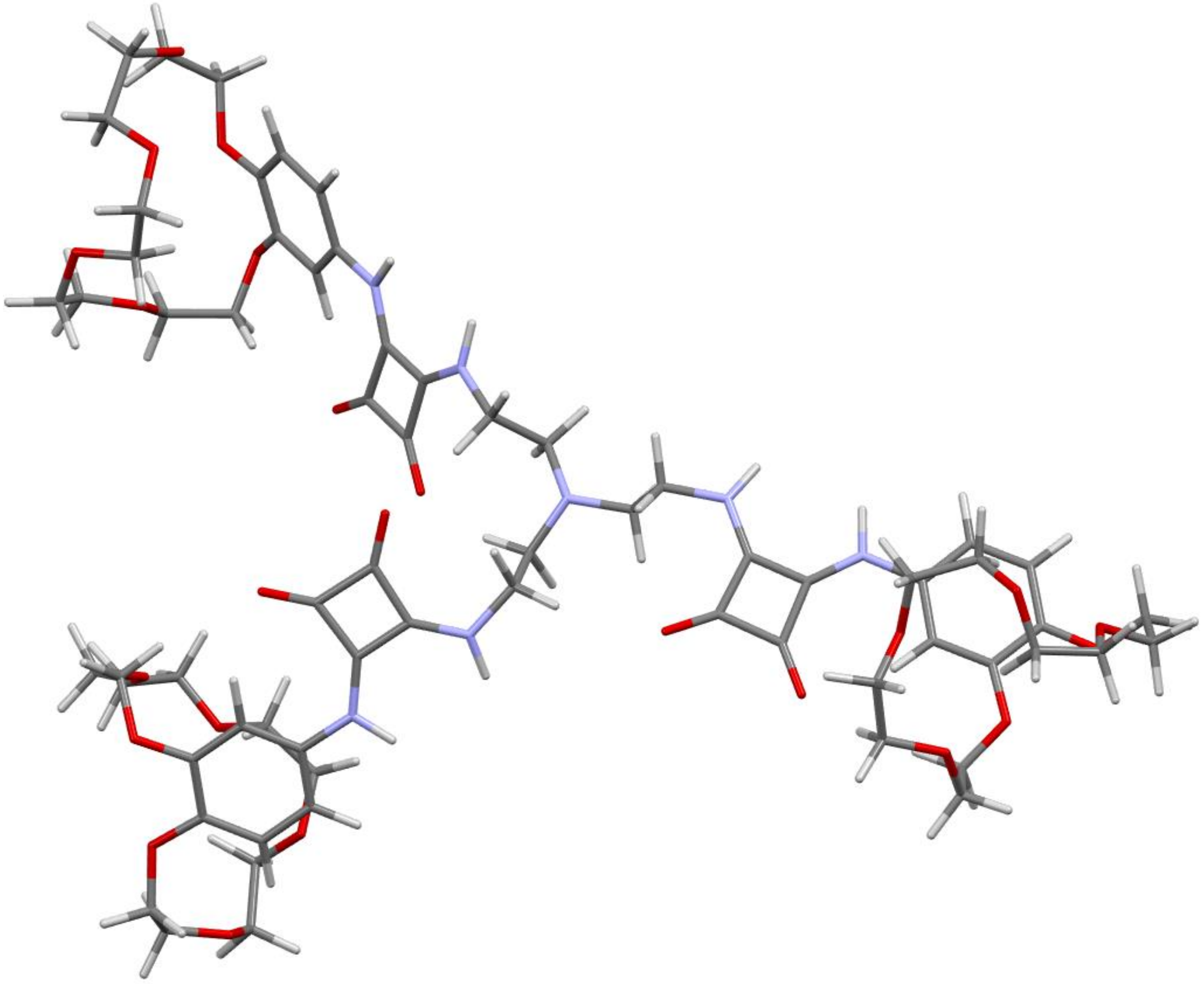

Table 2: Atomic coordinates for **2**.

Atom	X	Y	Z	32N	-0,98734	2,031931	1,488057
1C	-1,20341	-0,04182	0,109884	33H	-1,41183	2,785267	0,961154
2H	-1,55785	0,50048	-0,78372	34N	1,088013	4,499494	1,527095
3H	-0,11167	-0,05478	0,046818	35H	0,192324	4,753286	1,128296
4C	-1,62105	0,712293	1,384656	36N	1,461954	-3,45013	0,529083
5H	-2,70606	0,848153	1,430011	37H	1,448614	-4,44664	0,698301
6H	-1,31116	0,125843	2,251985	38N	4,345191	-4,59258	-0,2869
7C	0,293189	2,158132	1,885737	39H	3,872274	-5,1907	0,380042
8C	1,243104	1,203179	2,494423	40N	-5,33063	-2,481	0,115847
9C	2,26273	2,341272	2,556692	41H	-5,53548	-3,30637	-0,43313
10C	1,217401	3,215569	1,934456	42N	-8,31462	-2,16828	-1,02
11C	-0,69527	-2,41755	-0,25294	32N	-0,98734	2,031931	1,488057
12H	-0,12111	-2,10617	-1,1403	33H	-1,41183	2,785267	0,961154
13H	-1,20615	-3,35419	-0,50876	34N	1,088013	4,499494	1,527095
14C	0,280783	-2,67081	0,913078	35H	0,192324	4,753286	1,128296
15H	0,620682	-1,72598	1,343516	36N	1,461954	-3,45013	0,529083
16H	-0,2301	-3,21327	1,7145	37H	1,448614	-4,44664	0,698301
17C	2,500288	-2,91576	-0,13917	38N	4,345191	-4,59258	-0,2869
18C	2,732736	-1,58821	-0,75379	39H	3,872274	-5,1907	0,380042
19C	4,097613	-2,1078	-1,21724	40N	-5,33063	-2,481	0,115847
20C	3,763122	-3,39318	-0,52635	41H	-5,53548	-3,30637	-0,43313
21C	-3,0319	-1,61284	-0,42199	42N	-8,31462	-2,16828	-1,02
22H	-3,01634	-2,17396	-1,37172	43H	-7,94981	-3,10787	-1,11845
23H	-3,47135	-0,63439	-0,63874	44O	1,211272	0,023189	2,80227
24C	-3,95089	-2,32005	0,590267	45O	3,40626	2,462607	2,952162
25H	-3,56791	-3,31553	0,836966	46O	2,101269	-0,55197	-0,85483
26H	-3,97234	-1,73493	1,512065	47O	5,010471	-1,66969	-1,89023
27C	-6,18243	-1,43902	0,049731	48O	-5,22326	0,658213	1,012304
28C	-6,08282	-0,03217	0,492167	49O	-8,28078	1,107194	-0,09318
29C	-7,50733	0,169604	-0,02678	50O	9,412335	-6,43858	-1,75845
30C	-7,49667	-1,27405	-0,42275	51O	9,9365	-8,18464	0,763077
31N	-1,69585	-1,42288	0,12652	52O	9,112568	-6,03551	2,501584

53O	10,49983	-3,3383	1,75473	86C	11,01527	-2,35075	-0,45962
54O	9,762529	-2,56477	-1,08283	87H	11,49164	-1,43966	-0,86485
55O	8,373992	-4,3665	-3,02026	88H	11,6937	-3,19736	-0,65501
56C	5,623458	-5,06482	-0,67458	89C	9,836769	-2,50548	-2,49612
57C	6,364159	-4,4386	-1,68185	90H	10,63431	-3,1638	-2,87446
58H	5,967811	-3,56833	-2,18979	91H	10,06221	-1,47395	-2,8215
59C	7,627494	-4,91854	-2,01728	92C	8,510735	-2,9436	-3,0916
60C	8,153696	-6,06135	-1,37312	93H	7,683513	-2,42813	-2,59388
61C	7,395293	-6,68821	-0,38442	94H	8,490825	-2,68611	-4,15609
62H	7,821999	-7,51275	0,172926	95O	4,907519	8,56167	1,131971
63C	6,136873	-6,19495	-0,03494	96O	3,56061	9,9888	-1,33989
64H	5,5786	-6,67925	0,763594	97O	3,199977	7,454142	-2,67064
65C	9,845943	-7,78792	-1,61358	98O	5,8252	5,784151	-2,70708
66H	10,43631	-8,00042	-2,5129	99O	6,675196	5,121856	0,11756
67H	8,993919	-8,47814	-1,58786	100O	5,466175	6,405919	2,536352
68C	10,73309	-7,99935	-0,39375	101C	2,053838	5,533831	1,458072
69H	11,36505	-8,8893	-0,55858	102C	3,309167	5,419175	2,064725
70H	11,39712	-7,12912	-0,29589	103H	3,560323	4,532571	2,634565
71C	10,56255	-7,84451	1,994196	104C	4,239818	6,44647	1,933352
72H	11,61583	-8,16673	1,999574	105C	3,914501	7,621887	1,218297
73H	10,02889	-8,40786	2,766475	106C	2,653073	7,729803	0,635371
74C	10,47916	-6,35938	2,316243	107H	2,425936	8,586669	0,014729
75H	11,05689	-6,15929	3,23657	108C	1,724977	6,694013	0,754268
76H	10,9229	-5,75199	1,517448	109H	0,760614	6,785536	0,259025
77C	8,849265	-4,6928	2,872043	110C	4,601527	9,922421	0,841626
78H	9,42047	-4,41735	3,774244	111H	5,334343	10,50251	1,414281
79H	7,782637	-4,67476	3,120872	112H	3,598028	10,18924	1,195919
80C	9,106023	-3,64846	1,790947	113C	4,753797	10,28227	-0,6328
81H	8,752467	-4,01063	0,819116	114H	4,978683	11,3596	-0,71775
82H	8,536576	-2,73856	2,043365	115H	5,612526	9,726979	-1,03453
83C	10,83897	-2,16428	1,040923	116C	3,720031	9,770102	-2,73639
84H	11,7992	-1,82786	1,451836	117H	4,406935	10,51339	-3,17212
85H	10,09387	-1,37327	1,220185	118H	2,728396	9,921144	-3,17438

119C	4,20502	8,366941	-3,07436	152H	-9,91585	-0,03682	-0,78061
120H	4,37859	8,301635	-4,16342	153C	-9,63369	-2,0231	-1,51865
121H	5,16066	8,14693	-2,58218	154C	-11,9087	1,623954	-1,30346
122C	3,450661	6,092731	-2,97658	155H	-12,1032	2,33248	-2,11999
123H	3,673117	5,967898	-4,0496	156H	-10,8285	1,580532	-1,15661
124H	2,508122	5,578587	-2,75859	157C	-12,6258	2,129295	-0,05285
125C	4,553842	5,424719	-2,16417	158H	-12,2978	3,164912	0,140416
126H	4,476454	5,706814	-1,1085	159H	-13,703	2,147452	-0,24927
127H	4,422095	4,332138	-2,23404	160C	-11,2255	1,448829	1,779676
128C	6,912453	4,984412	-2,28033	161H	-11,2072	2,389577	2,35514
129H	7,677447	5,071974	-3,06207	162H	-10,3721	1,455931	1,091229
130H	6,612306	3,927183	-2,21191	163C	-11,0855	0,273731	2,731516
131C	7,543045	5,411691	-0,96214	164H	-10,1988	0,434031	3,367451
132H	8,497095	4,866046	-0,84406	165H	-11,9687	0,221196	3,385495
133H	7,776425	6,488911	-0,99105	166C	-11,1177	-2,14441	2,644165
134C	7,290971	5,301097	1,380444	167H	-11,3074	-1,98486	3,714457
135H	7,78305	6,28437	1,439806	168H	-10,1783	-2,71196	2,550762
136H	8,060437	4,524803	1,541566	169C	-12,2613	-2,97139	2,053416
137C	6,243031	5,206417	2,475587	170H	-12,208	-2,94467	0,957507
138H	5,618661	4,320505	2,325557	171H	-12,1352	-4,01246	2,377691
139H	6,743752	5,116935	3,445591	172C	-14,1308	-1,44638	1,895013
140O	-12,4486	0,363327	-1,71164	173H	-13,3754	-0,79122	1,454821
141O	-12,4633	1,317139	1,092388	174H	-14,6509	-0,8885	2,685294
142O	-10,9583	-0,90613	1,958437	175C	-15,1517	-1,80592	0,829904
143O	-13,546	-2,58636	2,522199	176H	-15,8595	-2,56141	1,209225
144O	-14,4908	-2,28657	-0,3317	177H	-15,7267	-0,8963	0,585104
145O	-13,5069	-1,70565	-3,02455	178C	-15,3092	-2,28291	-1,47981
146C	-10,1911	-3,10663	-2,19881	179H	-15,7276	-1,2781	-1,65293
147C	-11,4915	-3,00112	-2,69021	180H	-16,1549	-2,98691	-1,37205
148H	-11,9249	-3,82952	-3,24195	181C	-14,4814	-2,69469	-2,68392
149C	-12,243	-1,8427	-2,50744	182H	-14,008	-3,66433	-2,49233
150C	-11,6637	-0,74014	-1,83396	183H	-15,1371	-2,79322	-3,55596
151C	-10,3626	-0,84454	-1,34004	184H	-9,61919	-4,01817	-2,35866

Fig. S85: DFT calculated structure of the complex **2** and KCl, with E = - 5755.0122 au.

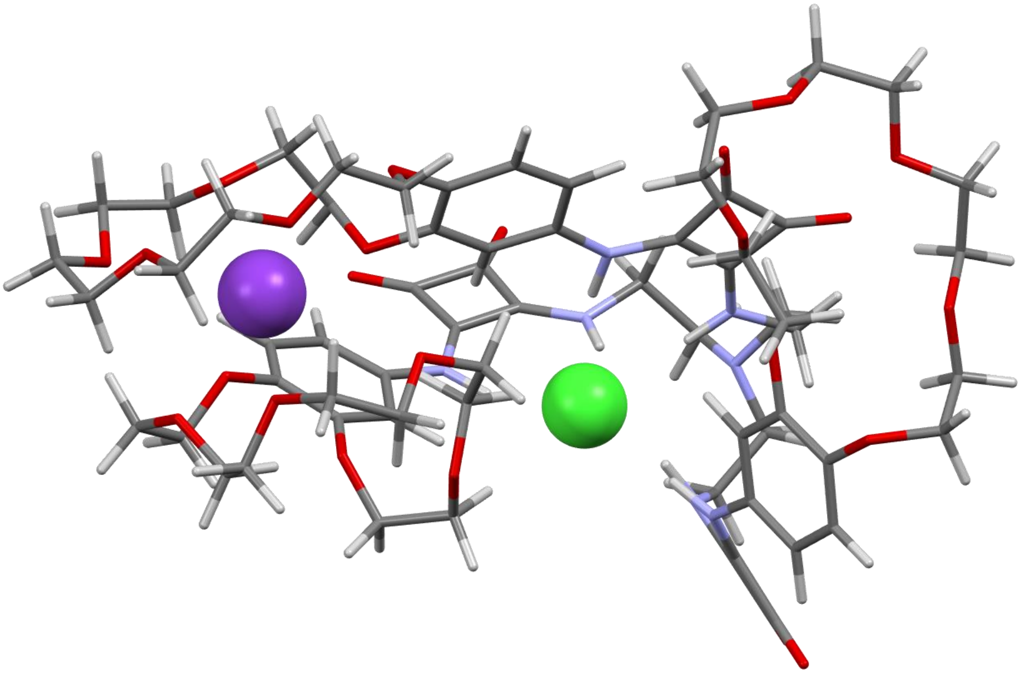

Table 3: Atomic coordinates for complex **2** and KCl.

Atom	X	Y	Z	31N	-4,92058	4,512872	-1,46167
1C	-5,66492	3,412128	-2,12572	32N	-4,189	1,582596	-1,37464
2H	-5,24224	3,242986	-3,10503	33H	-3,48661	2,151062	-0,9412
3H	-6,71245	3,679937	-2,25923	34N	-1,41246	0,031952	-1,71033
4C	-5,56589	2,09538	-1,34402	35H	-1,31595	0,91357	-1,23501
5H	-5,87134	2,239954	-0,31575	36N	-2,06583	4,896614	-2,27967
6H	-6,20865	1,351005	-1,78942	37H	-2,21057	4,298051	-1,48851
7C	-3,86811	0,379297	-1,82857	38N	0,765192	4,620617	-0,82824
8C	-4,69151	-0,74097	-2,35167	39H	0,006152	4,247245	-0,2846
9C	-3,38387	-1,50794	-2,55758	40N	-4,18978	4,515859	1,438868
10C	-2,69004	-0,3322	-1,99483	41H	-3,4782	4,018927	0,939183
11C	-4,44024	5,533391	-2,42504	42N	-3,88847	1,655388	2,820239
12H	-4,17186	6,422543	-1,87259	43H	-3,14205	1,799387	2,163343
13H	-5,22658	5,803551	-3,13005	44O	-5,87207	-0,92718	-2,5191
14C	-3,20054	5,077082	-3,19486	45O	-3,07465	-2,59644	-2,99625
15H	-3,39705	4,151066	-3,72172	46O	-0,82309	6,732	-4,63196
16H	-2,92511	5,824448	-3,92386	47O	2,176411	6,413728	-3,30166
17C	-0,85039	5,354603	-2,55455	48O	-6,25239	5,544897	3,713291
18C	-0,33485	6,194998	-3,6713	49O	-5,92933	2,663218	5,299533
19C	1,055046	6,053895	-3,02895	50Cl	-1,56616	2,656002	0,414509
20C	0,396725	5,256188	-1,97173	51C	0,969771	-0,03951	-1,56313
21C	-5,71572	5,146781	-0,37662	52C	1,043218	-2,67849	-2,2851
22H	-6,39104	4,410853	0,037041	53C	2,173967	-0,70877	-1,60904
23H	-6,3222	5,96164	-0,77057	54C	-0,22674	-0,68957	-1,8525
24C	-4,82178	5,650969	0,765274	55C	-0,1709	-2,02179	-2,248
25H	-4,0546	6,313514	0,389133	56C	2,225021	-2,04851	-1,9434
26H	-5,4192	6,185512	1,489723	57O	3,343858	-0,00559	-1,33068
27C	-4,69861	3,983413	2,549092	58O	3,448338	-2,71571	-1,92612
28C	-5,64357	4,515632	3,568398	59C	3,877643	0,752951	-2,47165
29C	-5,49233	3,160833	4,291379	60C	3,49212	-3,92808	-1,10842
30C	-4,58513	2,769081	3,182075	61C	4,892004	-4,49453	-1,18064

62C	4,960293	-0,04636	-3,17167	95K	5,603393	-0,98542	0,175811
63O	6,177278	-0,11158	-2,39547	96H	-1,06369	-2,53482	-2,5283
64O	5,724993	-3,84288	-0,19464	97C	4,369111	3,833909	-0,71537
65C	6,957863	1,104092	-2,31439	98C	2,07241	3,356608	0,697814
66C	8,380397	0,695647	-2,00602	99C	4,402018	2,970059	0,353598
67C	7,011863	-4,4743	-0,0229	100C	3,195662	4,442654	-1,12547
68C	7,849316	-3,62242	0,904905	101C	2,021666	4,17078	-0,43699
69O	8,220046	-2,45651	0,149564	102C	3,24613	2,764479	1,095327
70O	8,40564	0,211693	-0,65009	103O	5,571709	2,264532	0,647166
71C	9,556805	-0,56975	-0,27773	104O	3,339219	1,936675	2,193997
72C	9,125152	-1,54108	0,798946	105C	6,322196	2,718414	1,80604
73H	1,060611	-3,7046	-2,59056	106C	2,550419	2,156671	3,396677
74H	0,970512	0,990932	-1,27112	107C	1,288112	1,302949	3,441002
75H	3,234819	-3,68133	-0,09141	108C	7,416585	1,699303	2,06191
76H	2,795358	-4,6499	-1,51073	109O	6,922296	0,375692	2,35544
77H	3,068991	0,967613	-3,15666	110O	1,592198	-0,07317	3,165484
78H	4,248208	1,672401	-2,06104	111C	5,973986	0,247068	3,437953
79H	4,645028	-1,06818	-3,29582	112C	5,967396	-1,2071	3,857246
80H	5,170927	0,399326	-4,13898	113C	1,430914	-0,43745	1,770588
81H	6,584986	1,737558	-1,52566	114C	1,455893	-1,94359	1,649239
82H	6,927284	1,627929	-3,26508	115O	2,80666	-2,46154	1,759094
83H	8,677279	-0,08975	-2,68803	116O	5,548013	-2,01002	2,734126
84H	9,048467	1,543185	-2,11594	117C	4,69421	-3,14574	3,02368
85H	9,912158	-1,13769	-1,1277	118C	3,242382	-2,7241	3,109223
86H	10,34987	0,077494	0,07861	119H	1,163616	3,154673	1,221
87H	9,992253	-2,07646	1,176224	120H	5,277883	4,019442	-1,25271
88H	8,622172	-1,02748	1,603014	121H	3,192869	5,109318	-1,96044
89H	8,738073	-4,17362	1,200097	122H	3,20486	1,862852	4,202315
90H	7,283677	-3,3378	1,778157	123H	2,304058	3,2058	3,497704
91H	6,874913	-5,46699	0,393631	124H	6,771478	3,68101	1,594608
92H	7,520896	-4,55199	-0,97461	125H	5,654972	2,820144	2,646153
93H	5,296017	-4,34328	-2,17213	126H	8,00268	1,571917	1,169982
94H	4,856413	-5,55786	-0,96548	127H	8,037275	2,048801	2,881934

128H	4,996546	0,547688	3,097257	161O	-3,47775	-6,49151	-2,63002
129H	6,279515	0,859971	4,282108	162C	-4,19254	-5,78463	-3,65869
130H	6,959914	-1,51125	4,160287	163C	-5,66327	-5,88102	-3,31145
131H	5,285588	-1,32896	4,691107	164H	-6,19882	-1,72984	4,848029
132H	4,820012	-3,81658	2,191825	165H	-3,32969	-0,29727	1,178498
133H	5,013664	-3,62318	3,943643	166H	-7,3806	-4,78703	2,819446
134H	2,653064	-3,53152	3,531723	167H	-7,74739	-3,22022	3,553379
135H	3,109453	-1,84062	3,713044	168H	-3,49879	-4,45073	1,947108
136H	1,102638	-2,2344	0,674348	169H	-4,93354	-4,56221	0,917015
137H	0,82076	-2,38195	2,408688	170H	-3,61227	-4,33174	-1,07214
138H	0,475517	-0,0707	1,415923	171H	-2,18545	-3,86099	-0,13613
139H	2,221244	-0,00962	1,180332	172H	-1,8785	-7,5023	-0,80362
140H	0,538039	1,667252	2,751132	173H	-0,79696	-6,11092	-0,71828
141H	0,879675	1,352633	4,441399	174H	-2,02179	-5,02121	-2,55921
142C	-3,95022	-0,59183	1,998125	175H	-1,43444	-6,60827	-3,03503
143C	-5,57422	-1,39874	4,042788	176H	-3,91171	-4,74166	-3,67768
144C	-4,41437	-1,89658	2,015374	177H	-4,00238	-6,23617	-4,62753
145C	-4,3207	0,322995	2,970858	178H	-6,26686	-5,64072	-4,18294
146C	-5,12926	-0,09244	4,021554	179H	-5,88924	-6,89077	-2,99261
147C	-5,24838	-2,29828	3,048431	180H	-7,91832	-4,69705	-2,76413
148O	-4,04571	-2,67755	0,961549	181H	-7,6304	-5,76422	-1,38823
149O	-5,76339	-3,59318	3,060518	182H	-8,53349	-3,36462	-0,97221
150C	-3,95632	-4,13817	1,022466	183H	-6,8616	-2,86721	-1,24177
151C	-7,1832	-3,72885	2,78167	184H	-7,15586	-2,21215	1,254735
152C	-7,59498	-3,18721	1,422614	185H	-8,67653	-3,07879	1,408131
153C	-3,07088	-4,48795	-0,1561	186H	-5,41784	0,593735	4,787574
154O	-2,72816	-5,87606	0,013371	161O	-3,47775	-6,49151	-2,63002
155O	-7,18694	-4,11751	0,409613	162C	-4,19254	-5,78463	-3,65869
156C	-1,8154	-6,43253	-0,93277	163C	-5,66327	-5,88102	-3,31145
157C	-2,13	-6,08371	-2,38435	164H	-6,19882	-1,72984	4,848029
158C	-7,49556	-3,68523	-0,93328	165H	-3,32969	-0,29727	1,178498
159C	-7,30593	-4,85558	-1,88008	166H	-7,3806	-4,78703	2,819446
160O	-5,92534	-4,93657	-2,26102	167H	-7,74739	-3,22022	3,553379

168H	-3,49879	-4,45073	1,947108	182H	-8,53349	-3,36462	-0,97221
169H	-4,93354	-4,56221	0,917015	183H	-6,8616	-2,86721	-1,24177
170H	-3,61227	-4,33174	-1,07214	184H	-7,15586	-2,21215	1,254735
171H	-2,18545	-3,86099	-0,13613	185H	-8,67653	-3,07879	1,408131
172H	-1,8785	-7,5023	-0,80362	186H	-5,41784	0,593735	4,787574
173H	-0,79696	-6,11092	-0,71828	182H	-8,53349	-3,36462	-0,97221
174H	-2,02179	-5,02121	-2,55921	183H	-6,8616	-2,86721	-1,24177
175H	-1,43444	-6,60827	-3,03503	184H	-7,15586	-2,21215	1,254735
176H	-3,91171	-4,74166	-3,67768	185H	-8,67653	-3,07879	1,408131
177H	-4,00238	-6,23617	-4,62753	186H	-5,41784	0,593735	4,787574
178H	-6,26686	-5,64072	-4,18294				
179H	-5,88924	-6,89077	-2,99261				
180H	-7,91832	-4,69705	-2,76413				
181H	-7,6304	-5,76422	-1,38823				
182H	-8,53349	-3,36462	-0,97221				
183H	-6,8616	-2,86721	-1,24177				
184H	-7,15586	-2,21215	1,254735				
185H	-8,67653	-3,07879	1,408131				
186H	-5,41784	0,593735	4,787574				
168H	-3,49879	-4,45073	1,947108				
169H	-4,93354	-4,56221	0,917015				
170H	-3,61227	-4,33174	-1,07214				
171H	-2,18545	-3,86099	-0,13613				
172H	-1,8785	-7,5023	-0,80362				
173H	-0,79696	-6,11092	-0,71828				
174H	-2,02179	-5,02121	-2,55921				
175H	-1,43444	-6,60827	-3,03503				
176H	-3,91171	-4,74166	-3,67768				
177H	-4,00238	-6,23617	-4,62753				
178H	-6,26686	-5,64072	-4,18294				
179H	-5,88924	-6,89077	-2,99261				
180H	-7,91832	-4,69705	-2,76413				
181H	-7,6304	-5,76422	-1,38823				

Fig. S86: DFT calculated structure of the complex **2** and K_2SO_4 with E = **-** 13242.3183 au.

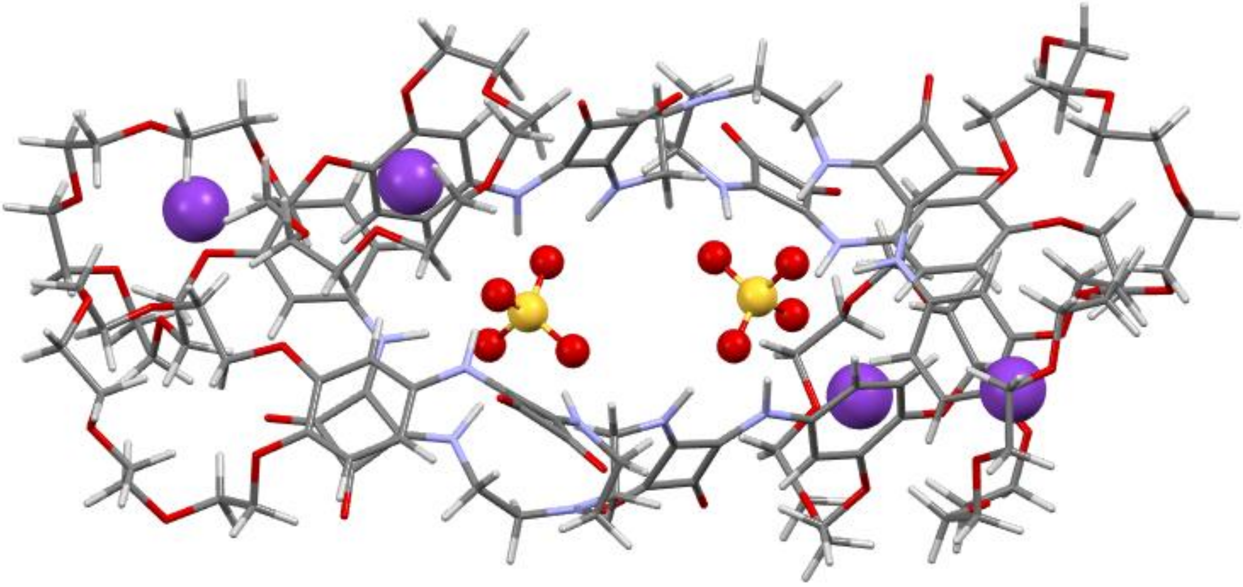

Table 4: Atomic coordinates for complex **2** and K_2SO_4.

Atom	X	Y	Z	32N	3,252961	2,623387	2,948078
1C	2,333563	1,86341	5,151742	33H	3,130626	1,839817	2,160646
2H	3,340764	2,05832	5,5832	34N	6,144208	2,473465	1,498698
3H	1,594536	2,207904	5,91653	35H	5,494909	2,101356	0,734548
4C	2,126102	2,750254	3,892182	36N	4,894788	-0,88254	3,251272
5H	1,198436	2,443638	3,346227	37H	4,228885	-0,77521	2,412978
6H	1,982585	3,807881	4,213032	38N	6,239907	-2,27614	0,71558
7C	4,321471	3,384049	3,028842	39H	5,345473	-1,69629	0,51531
8C	4,696527	4,657197	3,808386	40N	-0,55565	-0,97831	2,680584
9C	6,044791	4,618173	3,012174	41H	-1,09069	-0,34603	1,974841
10C	5,593307	3,399464	2,385561	42N	-3,51979	-2,30938	2,395784
11C	3,257444	-0,53016	5,141682	43H	-3,3542	-1,71096	1,524869
12H	2,936532	-1,53783	4,778776	44O	4,145212	5,297388	4,646373
13H	3,426538	-0,64034	6,241459	45O	7,002322	5,354566	3,014349
14C	4,610498	-0,14479	4,500741	46O	7,283159	-1,9676	5,249466
15H	4,643942	0,9453	4,263908	47O	8,524002	-3,7418	2,780494
16H	5,425559	-0,31817	5,245265	48O	0,394875	-3,35852	4,732422
17C	5,963052	-1,66235	3,14012	49O	-2,52904	-4,79621	4,442765
18C	7,035609	-2,17221	4,102286	50O	3,918703	-0,94099	0,762868
19C	7,616167	-2,95526	2,882983	51O	4,509994	1,186367	-0,35155
20C	6,57432	-2,3579	2,061939	52O	2,618517	-0,07484	-1,21276
21C	0,850835	-0,08134	4,50654	53O	2,500115	1,000461	1,06758
22H	0,079566	0,708356	4,650028	54S	3,351235	0,276136	0,035543
23H	0,561552	-0,93361	5,170562	55O	-3,29826	1,247807	-1,05004
24C	0,821658	-0,5648	3,034991	56O	-3,67828	-0,96212	0,020557
25H	1,148514	0,248086	2,320672	57O	-1,96483	0,514867	0,938165
26H	1,546821	-1,39355	2,873614	58O	-1,67553	-0,52745	-1,34344
27C	-1,11175	-2,05488	3,196843	59S	-2,62103	0,070502	-0,32709
28C	-0,62228	-3,16283	4,145839	60C	-1,42511	-1,31774	-5,44317
29C	-2,03667	-3,7956	3,972781	61H	-2,40134	-1,6768	-5,8371
30C	-2,39096	-2,6869	3,118468	62H	-0,67265	-1,50859	-6,24687
31N	2,155873	0,42498	4,942967	63C	-1,00909	-2,18031	-4,21729

64H	-0,12623	-1,72817	-3,70058	97N	-5,76758	2,215391	-0,82799
65H	-0,70058	-3,192	-4,56473	98H	-4,73424	1,890977	-0,70642
66C	-2,98604	-3,24117	-3,24077	99N	1,222421	1,522428	-2,93669
67C	-3,04701	-4,65407	-3,85325	100H	1,732486	0,872947	-2,24321
68C	-4,39062	-4,82567	-3,0335	101N	4,230443	2,752329	-2,65461
69C	-4,23314	-3,4499	-2,60434	102H	4,075482	2,000257	-1,92547
70C	-2,74856	0,881215	-5,31215	103O	-2,36267	-5,23645	-4,63266
71H	-2,58694	1,910121	-4,90828	104O	-5,12208	-5,7666	-2,90767
72H	-2,97552	1,008972	-6,39946	105O	-7,09887	1,414726	-5,23387
73C	-3,99962	0,251565	-4,65342	106O	-8,55038	2,876029	-2,69412
74H	-3,82957	-0,82487	-4,40862	107O	0,338613	3,96247	-4,949
75H	-4,84537	0,266686	-5,38359	108O	3,317732	5,272341	-4,70884
76C	-5,58501	1,466895	-3,23287	109K	-9,19811	6,496255	2,762859
77C	-6,82038	1,684096	-4,10457	110O	-9,66972	4,075901	1,489838
78C	-7,46774	2,354273	-2,85585	111O	-10,1566	6,560322	0,046179
79C	-6,25301	2,078486	-2,12434	112O	-10,6565	8,671045	1,448934
80C	-0,26884	0,835865	-4,80455	113O	-12,1594	6,65589	3,064693
81H	0,616264	0,234675	-5,11365	114O	-10,9073	4,488842	4,717718
82H	-0,21552	1,794126	-5,38105	115O	-8,10735	4,253381	3,814148
83C	-0,16765	1,155926	-3,29285	116C	-10,4162	4,211633	0,252313
84H	-0,47139	0,273763	-2,65463	117H	-11,4351	4,417895	0,638913
85H	-0,87876	1,9634	-3,00983	118H	-10,4209	3,229443	-0,28112
86C	1,813792	2,592305	-3,44136	119C	-9,89331	5,315912	-0,6614
87C	1,354872	3,717635	-4,37658	120H	-8,80618	5,23778	-0,87066
88C	2,790482	4,297553	-4,22876	121H	-10,4367	5,307567	-1,62664
89C	3,111891	3,174914	-3,36518	122C	-10,0545	7,728848	-0,78727
90N	-1,49121	0,124545	-5,19664	123H	-10,9165	7,730126	-1,48154
91N	-2,11231	-2,25634	-3,24263	124H	-9,12259	7,70364	-1,38508
92H	-2,16278	-1,4381	-2,50848	125C	-10,0763	8,939792	0,162019
93N	-5,05081	-2,56127	-1,89595	126H	-9,05623	9,279003	0,435579
94H	-4,6673	-2,1659	-1,00207	127H	-10,6176	9,784706	-0,30948
95N	-4,38655	0,938929	-3,4024	128C	-12,0625	8,303511	1,401509
96H	-3,65647	1,000419	-2,5859	129H	-12,1914	7,39105	0,77813

130H	-12,6275	9,146324	0,961558	163H	5,649267	-8,70259	-6,73364
131C	-12,4719	8,052964	2,851967	164H	7,397157	-8,31458	-6,45829
132H	-11,9237	8,689136	3,571725	165C	6,673599	-10,0379	-4,59957
133H	-13,5605	8,206557	2,989819	166H	7,6964	-10,1577	-5,0092
134C	-12,5788	6,130845	4,347525	167H	5,972805	-10,6856	-5,16374
135H	-13,6627	6,310115	4,478013	168C	6,620998	-10,3454	-3,10323
136H	-12,0244	6,622859	5,168324	169H	5,772044	-9,84626	-2,59694
137C	-12,2685	4,636097	4,255097	170H	6,558935	-11,4381	-2,92671
138H	-12,9174	4,050802	4,934688	171C	8,075946	-10,0987	-1,1683
139H	-12,3837	4,257148	3,219793	172H	8,086914	-11,1927	-0,99595
140C	-10,2986	3,232563	4,306861	173H	7,257988	-9,64319	-0,57693
141H	-10,4355	3,068822	3,217613	174C	9,436211	-9,47695	-0,84771
142H	-10,7934	2,416841	4,866229	175H	9,861935	-9,91602	0,077121
143C	-8,82772	3,360435	4,696649	176H	10,15834	-9,60244	-1,67827
144H	-8,70625	3,856175	5,681787	177C	10,36959	-7,28705	-0,37263
145H	-8,33314	2,370251	4,710625	178H	11,02898	-7,32297	-1,26071
146K	7,730349	-6,94487	-2,86009	179H	10,90503	-7,7064	0,502918
147O	8,816221	-4,51446	-3,72038	180C	9,871562	-5,8779	-0,06831
148O	7,240803	-6,02276	-5,60179	181H	9,009295	-5,87451	0,633793
149O	6,22725	-8,66551	-4,73448	182H	10,67739	-5,23866	0,353306
150O	7,884827	-9,85866	-2,58633	183K	-7,45981	-6,35576	2,414728
151O	9,16991	-8,07556	-0,60652	184O	-7,06324	-4,76857	4,778247
152O	9,524154	-5,29604	-1,36252	185O	-5,73994	-7,33016	4,557733
153C	8,510943	-4,09306	-5,05905	186O	-6,27172	-8,99388	2,126002
154H	9,349022	-4,55775	-5,62445	187O	-9,00335	-8,51681	1,065317
155H	8,587893	-2,98999	-5,13638	188O	-10,0218	-5,74533	1,198069
156C	7,148276	-4,57549	-5,57045	189O	-8,85763	-3,78626	3,186801
157H	6,304605	-4,25694	-4,92963	190C	-6,17159	-5,31591	5,777653
158H	6,969452	-4,21376	-6,6028	191H	-6,89815	-5,85932	6,418766
159C	6,011638	-6,67225	-6,00964	192H	-5,72123	-4,48058	6,352403
160H	5,713015	-6,28714	-7,00561	193C	-5,07514	-6,23418	5,241148
161H	5,199273	-6,46383	-5,28519	194H	-4,36366	-5,71035	4,554547
162C	6,364692	-8,15682	-6,08711	195H	-4,48612	-6,66174	6,077383

196C	-4,81579	-8,15783	3,805141	229C	-10,9518	0,536721	-0,0615
197H	-3,97689	-8,46029	4,465321	230H	-11,4696	-0,36788	0,294872
198H	-4,40255	-7,58838	2,946802	231H	-10,1621	0,83586	0,660617
199C	-5,63524	-9,37277	3,37494	232C	-13,1561	1,450575	-0,56827
200H	-4,98177	-10,2438	3,172028	233H	-13,094	0,993157	-1,57745
201H	-6,39594	-9,6485	4,131646	234H	-13,5298	2,486125	-0,66062
202C	-7,24493	-9,96265	1,665233	235C	-14,0126	0,619737	0,388855
203H	-7,95989	-10,2137	2,474272	236H	-13,506	0,492509	1,366654
204H	-6,70109	-10,8775	1,356159	237H	-15,0064	1,072963	0,541482
205C	-7,94321	-9,30991	0,472702	238C	-14,9786	-0,97234	-1,19777
206H	-7,2608	-8,67236	-0,12564	239H	-15,6344	-0,11104	-1,41376
207H	-8,39918	-10,0744	-0,18771	240H	-15,5854	-1,85127	-0,91248
208C	-9,82921	-7,8219	0,096436	241C	-14,0683	-1,28535	-2,38634
209H	-10,3949	-8,57689	-0,48456	242H	-13,0571	-1,58486	-2,05287
210H	-9,2078	-7,21517	-0,59453	243H	-14,4912	-2,06115	-3,04662
211C	-10,7675	-6,9568	0,939159	244C	-12,9817	-0,03213	-4,15921
212H	-11,6814	-6,69219	0,371504	245H	-13,3974	-0,50609	-5,06197
213H	-11,0537	-7,4516	1,887668	246H	-12,0829	-0,58751	-3,82504
214C	-10,7049	-4,80581	2,068759	247C	-12,6876	1,459903	-4,35642
215H	-10,8514	-5,24664	3,072583	248H	-13,5291	1,981206	-4,85056
216H	-11,6896	-4,55876	1,626207	249H	-12,4588	1,956557	-3,39729
217C	-9,79178	-3,57961	2,094319	250C	-10,2844	1,412746	-4,79722
218H	-9,29085	-3,42467	1,115253	251H	-10,1889	1,662158	-3,72022
219H	-10,3604	-2,66605	2,358243	252H	-9,69198	2,144436	-5,38576
220O	-9,02562	-0,15858	-1,14436	253C	-9,85679	-0,01976	-5,10083
221O	-11,8408	1,670804	-0,0276	254H	-10,5391	-0,5076	-5,81855
222O	-14,1736	-0,75041	-0,02122	255H	-8,82006	-0,05153	-5,48411
223O	-14,0041	-0,04444	-3,12932	256K	8,015517	7,308515	-1,89267
224O	-11,6214	1,648431	-5,29301	257O	9,394234	4,876048	-2,51416
225O	-9,99248	-0,83276	-3,892	258O	9,645626	6,211715	0,176133
226C	-10,3515	0,351207	-1,45855	259O	8,239796	8,774744	0,558564
227H	-10,9081	-0,36708	-2,08603	260O	7,797225	10,31798	-1,94261
228H	-10,2127	1,30577	-2,01045	261O	6,896598	8,663071	-4,23737

262O	7,68982	5,854491	-4,29203	295O	13,19248	-3,35217	2,286139
263C	10,22211	4,507211	-1,3845	296O	12,1121	-1,39521	4,84429
264H	11,12089	5,132485	-1,57181	297O	11,43709	1,792673	6,476909
265H	10,49589	3,436556	-1,48362	298O	10,69504	2,39564	3,341665
266C	9,616904	4,771591	-0,00419	299C	12,42299	0,849584	1,58608
267H	8,584766	4,38122	0,115234	300H	13,11919	0,389713	2,318761
268H	10,2421	4,315932	0,793655	301H	12,31297	1,92309	1,806325
269C	8,930623	6,650003	1,364214	302C	12,85248	0,567139	0,147696
270H	9,335591	6,115423	2,249554	303H	11,97561	0,358071	-0,49314
271H	7,848503	6,400848	1,290929	304H	13,45727	1,384066	-0,28566
272C	9,200521	8,148336	1,452791	305C	13,24399	-1,81512	0,48576
273H	9,001059	8,527172	2,475186	306H	13,55867	-2,50666	-0,31378
274H	10,23461	8,40227	1,152378	307H	12,14088	-1,80197	0,558896
275C	8,506179	10,17404	0,31145	308C	13,88171	-2,1633	1,837928
276H	9,499132	10,3006	-0,1638	309H	14,94144	-2,4483	1,744842
277H	8,48821	10,71563	1,277769	310H	13,78603	-1,32749	2,555796
278C	7,369879	10,64647	-0,59557	311C	12,68332	-3,31183	3,633743
279H	6,409657	10,14778	-0,35617	312H	13,5163	-3,12843	4,339109
280H	7,238687	11,74354	-0,52869	313H	12,31017	-4,34611	3,747778
281C	6,807331	10,63619	-2,95379	314C	11,57637	-2,27665	3,820587
282H	6,731304	11,73836	-3,02461	315H	11,36942	-1,70032	2,895509
283H	5,817348	10,21471	-2,68622	316H	10,63249	-2,71655	4,18875
284C	7,350553	10,03617	-4,25083	317C	11,29251	-0,21999	5,064234
285H	6,917998	10,54676	-5,13367	318H	10,27234	-0,52296	5,350568
286H	8,455343	10,08189	-4,30341	319H	11,25443	0,373594	4,126394
287C	7,280957	7,905217	-5,41392	320C	12,02747	0,509741	6,190187
288H	8,380342	7,911364	-5,53782	321H	11,90903	-0,00422	7,163513
289H	6,806192	8,36854	-6,30138	322H	13,09999	0,624057	5,962389
290C	6,725643	6,504755	-5,17417	323C	11,59448	2,812352	5,474918
291H	5,700769	6,520285	-4,73463	324H	12,47031	2,620297	4,829317
292H	6,702042	5,908605	-6,10851	325H	11,76443	3,721894	6,081221
293O	11,18739	0,136549	1,85667	326C	10,31088	2,92294	4,649211
294O	13,77366	-0,53811	0,064758	327H	9,496161	2,313331	5,08032

328H	9,975028	3,962163	4,502257	361C	-7,78642	3,1213	0,264133
329C	7,221797	4,785632	-3,52729	362H	-5,74814	3,222534	3,600274
330C	6,523556	2,558545	-2,0027	363H	-4,66777	2,37619	1,523758
331C	5,924267	4,287113	-3,62246	364H	-8,37108	3,016346	-0,65163
332C	8,158122	4,219444	-2,63266	365H	8,293564	-4,08505	0,772654
333C	7,8096	3,050421	-1,93437	366C	7,953299	-3,78942	-0,22527
334C	5,529238	3,217004	-2,79181	367C	7,139387	-2,90897	-2,78312
335H	5,203433	4,730439	-4,31353	368C	8,536378	-4,31616	-1,36924
336H	8,547724	2,526352	-1,32406	369C	6,91385	-2,83458	-0,34726
337H	6,236238	1,66897	-1,42121	370C	6,516531	-2,40733	-1,65489
338H	-4,48524	-3,81011	4,474641	371C	8,13699	-3,88428	-2,6607
339C	-5,23063	-3,46628	3,759779	372H	5,723601	-1,66329	-1,75439
340C	-7,15942	-2,41228	1,980407	373H	6,84937	-2,52235	-3,75933
341C	-6,56674	-3,84662	3,85833	374H	7,063248	0,551566	-8,9E-05
342C	-4,82957	-2,62827	2,692982	375C	7,813698	0,945045	0,693206
343C	-5,83147	-2,07691	1,828677	376C	9,671497	1,94063	2,525455
344C	-7,54219	-3,33694	2,969159	377C	7,446653	2,013074	1,572926
345H	-5,51431	-1,38006	1,037718	378C	9,074522	0,383799	0,754388
346H	-7,90465	-1,95845	1,322799	379C	10,01366	0,863805	1,68382
347C	-8,77746	-1,28158	-3,36642	380C	8,411106	2,529317	2,474215
348C	-6,30021	-2,19928	-2,37038	381H	9,345078	-0,45982	0,118449
349C	-8,34634	-0,90989	-2,07586	382H	8,169939	3,36821	3,118735
350C	-7,9947	-2,17492	-4,11923				
351C	-6,78499	-2,63881	-3,64117				
352C	-7,1082	-1,34103	-1,58683				
353H	-8,36042	-2,49195	-5,09554				
354H	-6,20022	-3,33728	-4,22981				
355H	-6,76624	-0,99001	-0,61309				
356C	-7,62462	3,676458	2,635017				
357C	-6,43893	2,687056	0,27279				
358C	-8,35611	3,622387	1,428262				
359C	-6,29506	3,200387	2,656092				
360C	-5,70052	2,732968	1,507602				

NMR SPECTRA

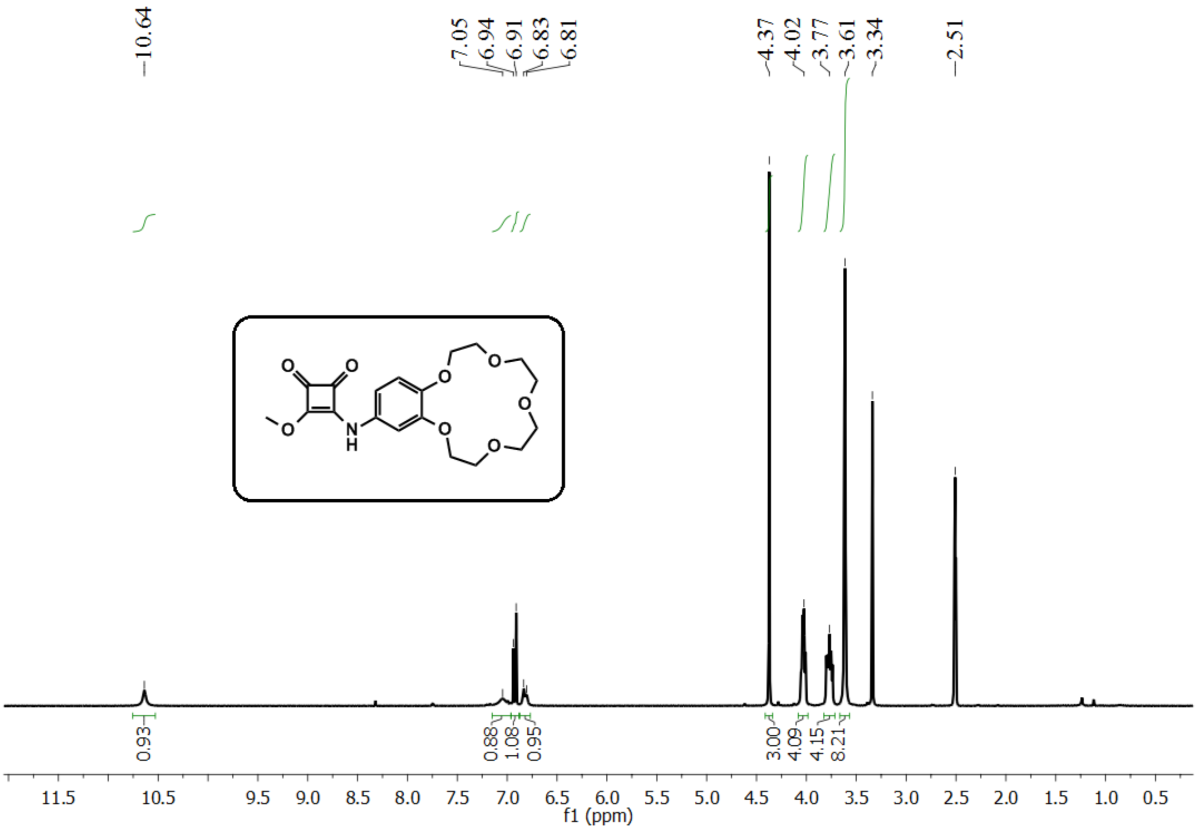
10.64
7.05
6.94
6.91
6.83
6.81
4.37
4.02
3.77
3.61
3.34
2.51
0.93
0.88
1.08
0.95
3.00
4.09
4.15
8.21
11.5
10.5
9.5
9.0
8.5
8.0
7.5
7.0
6.5
6.0
5.5
5.0
4.5
4.0
3.5
3.0
2.5
2.0
1.5
1.0
0.5
f1 (ppm)

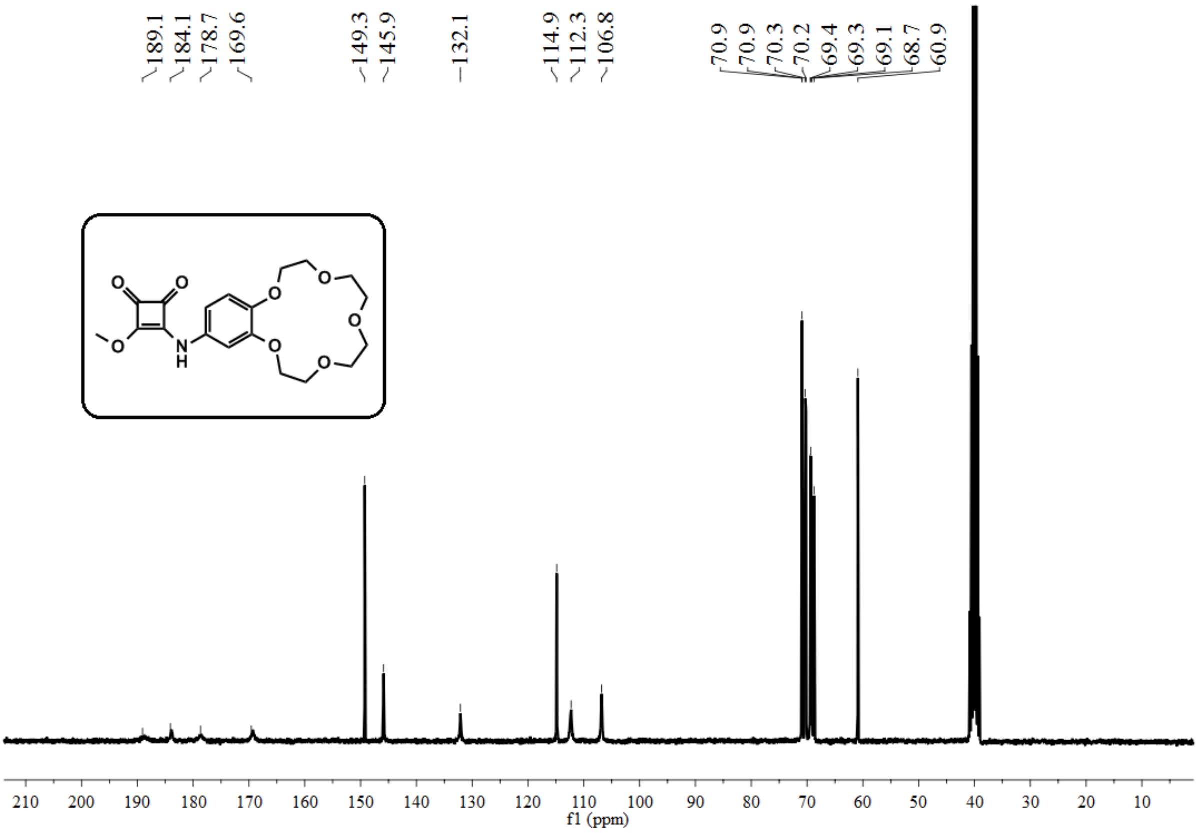
189.1
184.1
178.7
169.6
149.3
145.9
132.1
114.9
112.3
106.8
70.9
70.9
70.3
70.2
69.4
69.3
69.1
68.7
60.9
210
200
190
180
170
160
150
140
130
120
110
100
90
80
70
60
50
40
30
20
10
f1 (ppm)

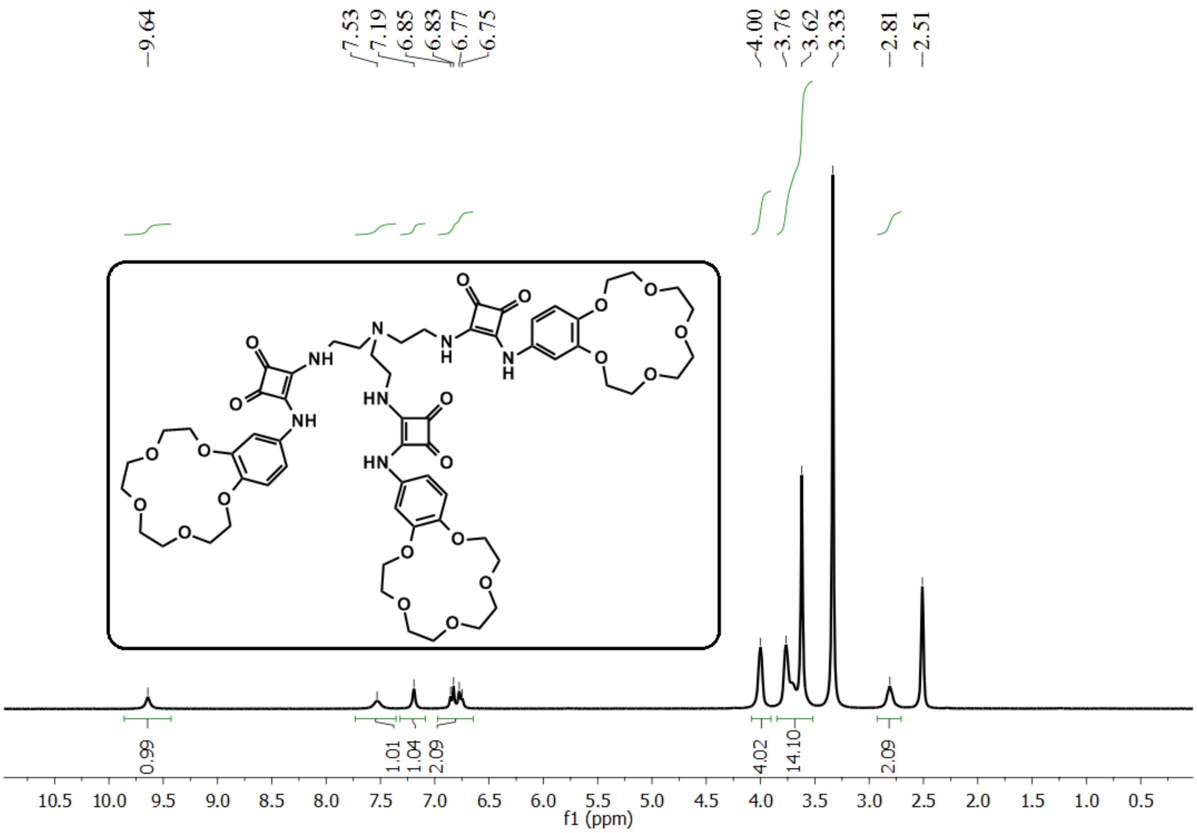
9.64
7.53
7.19
6.85
6.83
6.77
6.75
4.00
3.76
3.62
3.33
2.81
2.51
0.99
1.01
1.04
2.09
4.02
14.10
2.09
NH
HN
O
N
10.5 10.0 9.5 9.0 8.5 8.0 7.5 7.0 6.5 6.0 5.5 5.0 4.5 4.0 3.5 3.0 2.5 2.0 1.5 1.0 0.5
f1 (ppm)

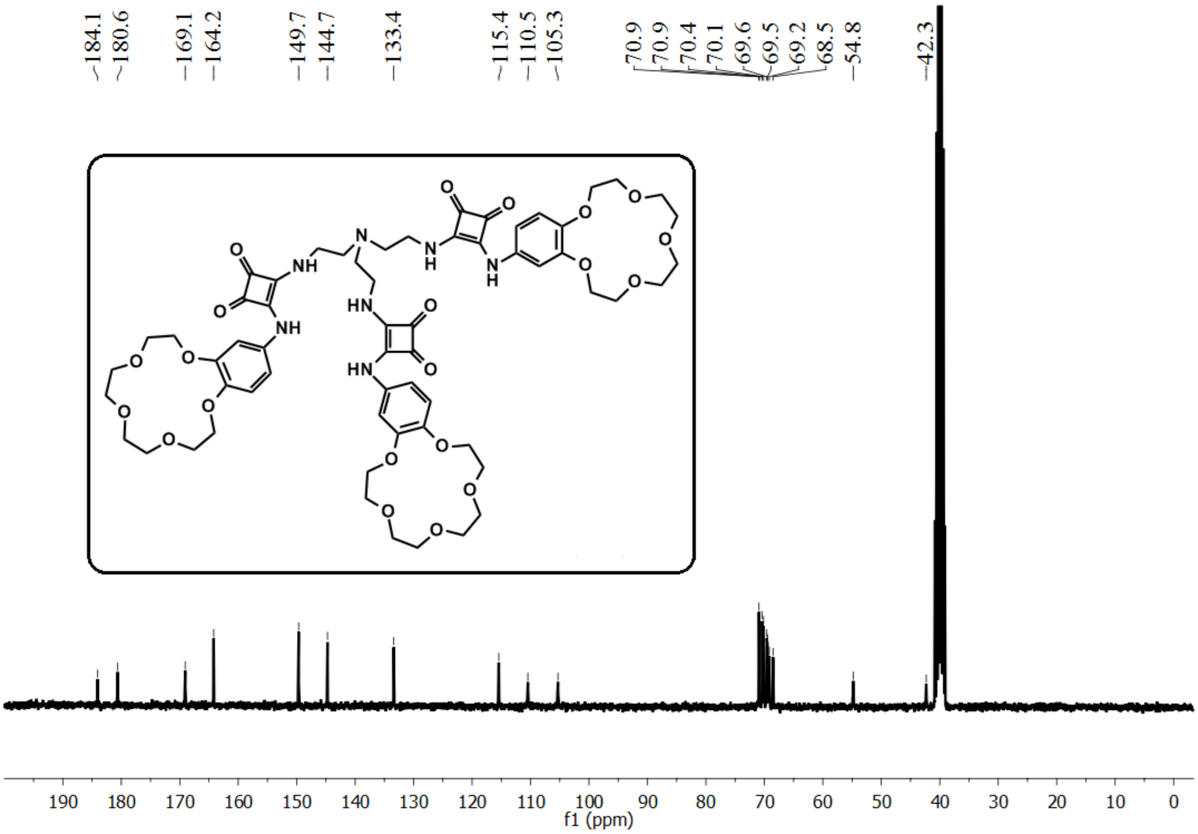
184.1
180.6
169.1
164.2
149.7
144.7
133.4
115.4
110.5
105.3
70.9
70.9
70.4
70.1
69.6
69.5
69.2
68.5
54.8
42.3
NH
HN
O
N
190 180 170 160 150 140 130 120 110 100 90 80 70 60 50 40 30 20 10 0
f1 (ppm)

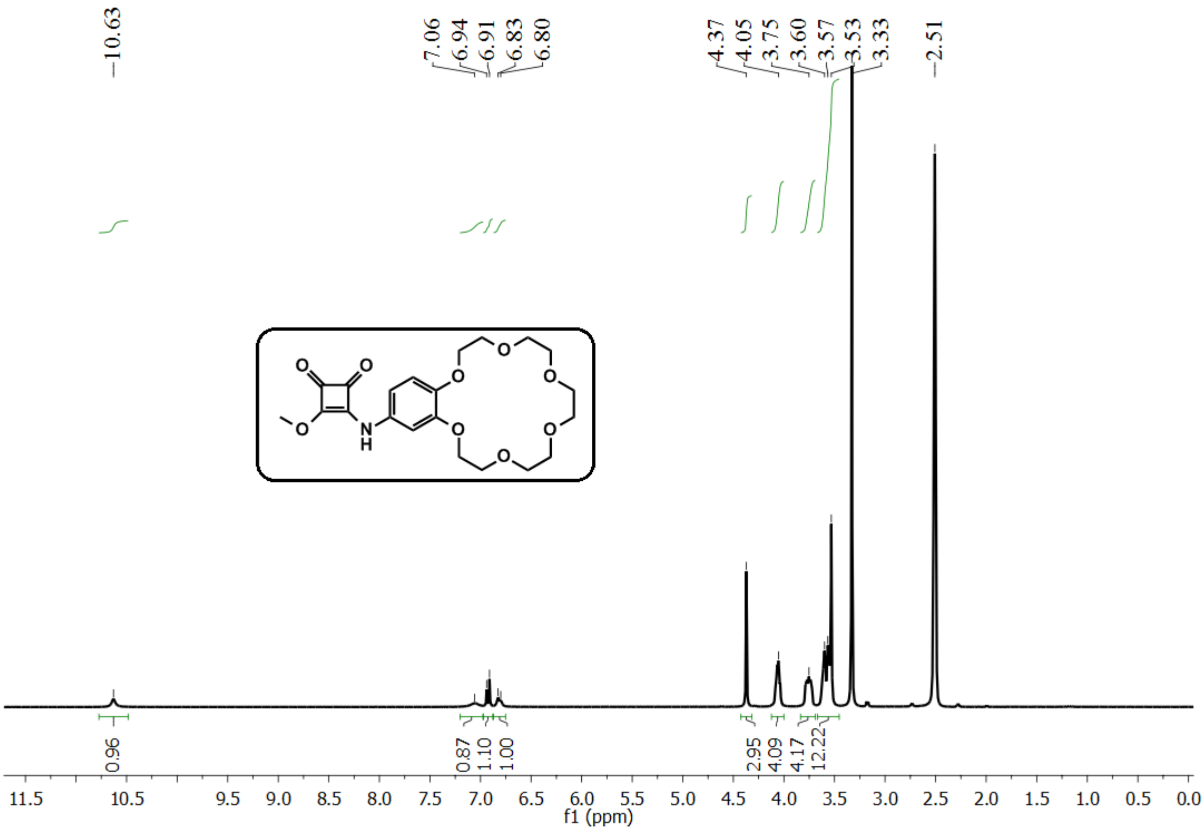
10.63
7.06
6.94
6.91
6.83
6.80
4.37
4.05
3.75
3.60
3.57
3.53
3.33
2.51
0.96
0.87
1.10
1.00
2.95
4.09
4.17
12.22
f1 (ppm)

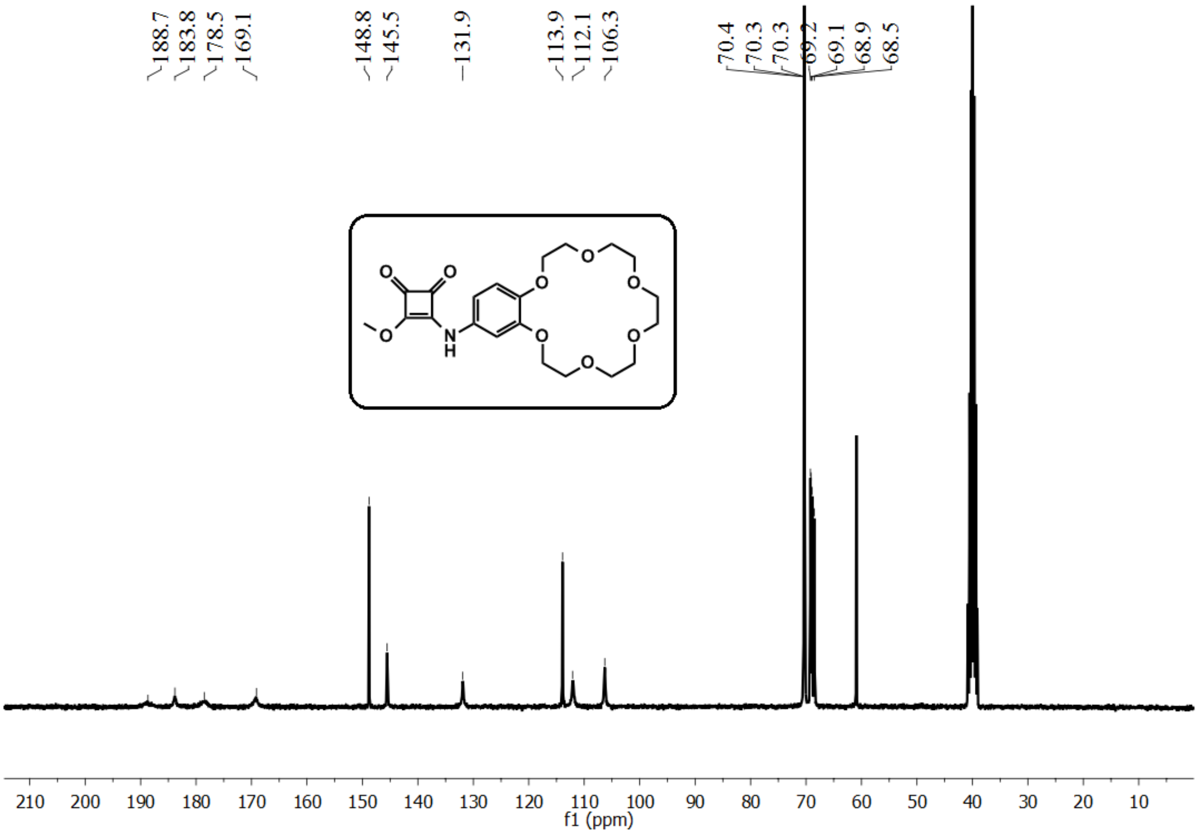
188.7
183.8
178.5
169.1
148.8
145.5
131.9
113.9
112.1
106.3
70.4
70.3
70.3
69.2
69.1
68.9
68.5
f1 (ppm)

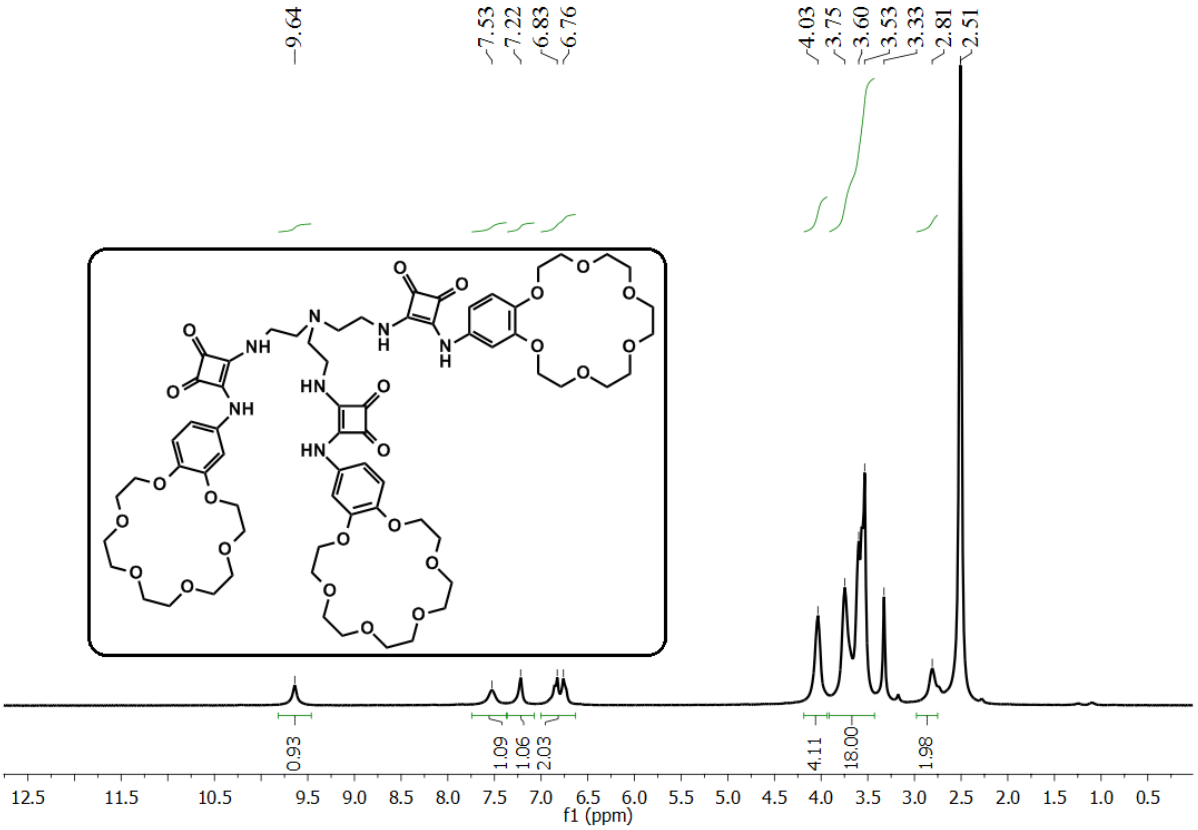
9.64
7.53
7.22
6.83
6.76
4.03
3.75
3.60
3.53
3.33
2.81
2.51
0.93
1.09
1.06
2.03
4.11
18.00
1.98
12.5 11.5 10.5 9.5 9.0 8.5 8.0 7.5 7.0 6.5 6.0 5.5 5.0 4.5 4.0 3.5 3.0 2.5 2.0 1.5 1.0 0.5
f1 (ppm)

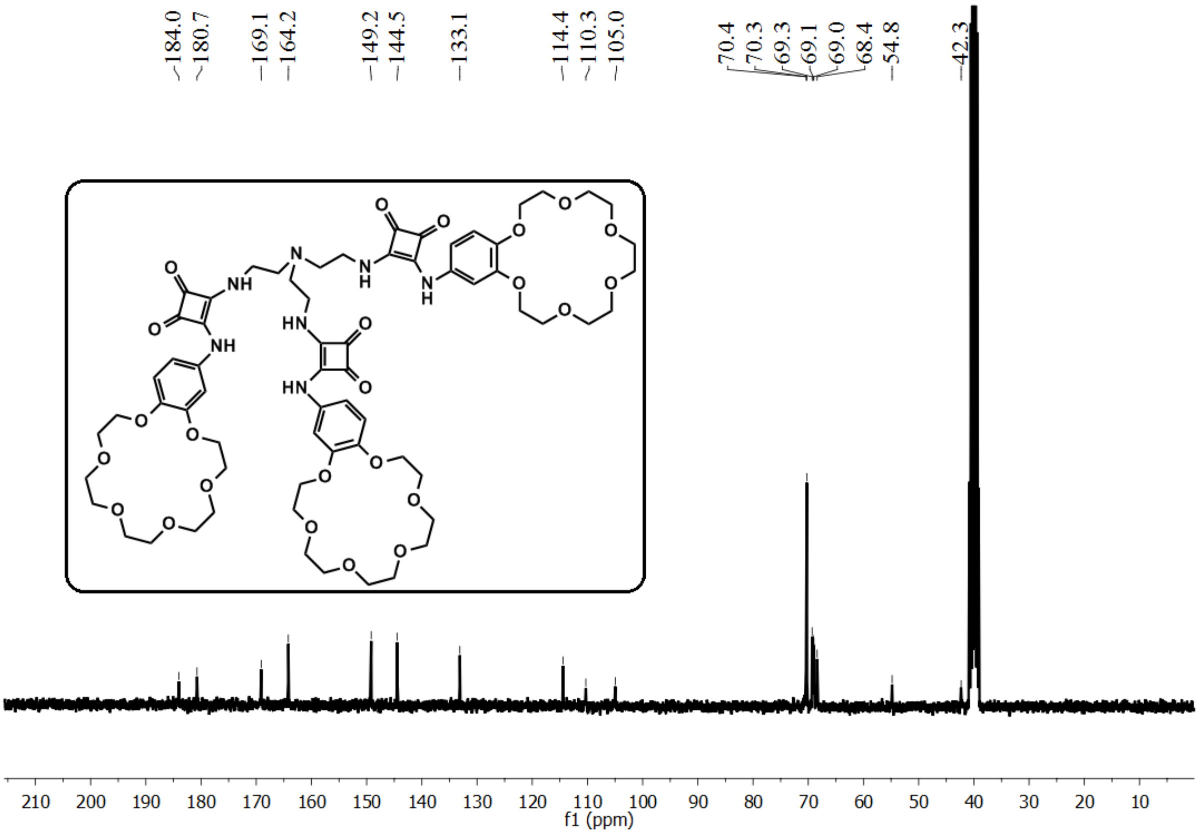
184.0
180.7
169.1
164.2
149.2
144.5
133.1
114.4
110.3
105.0
70.4
70.3
69.3
69.1
69.0
68.4
54.8
42.3
210 200 190 180 170 160 150 140 130 120 110 100 90 80 70 60 50 40 30 20 10
f1 (ppm)

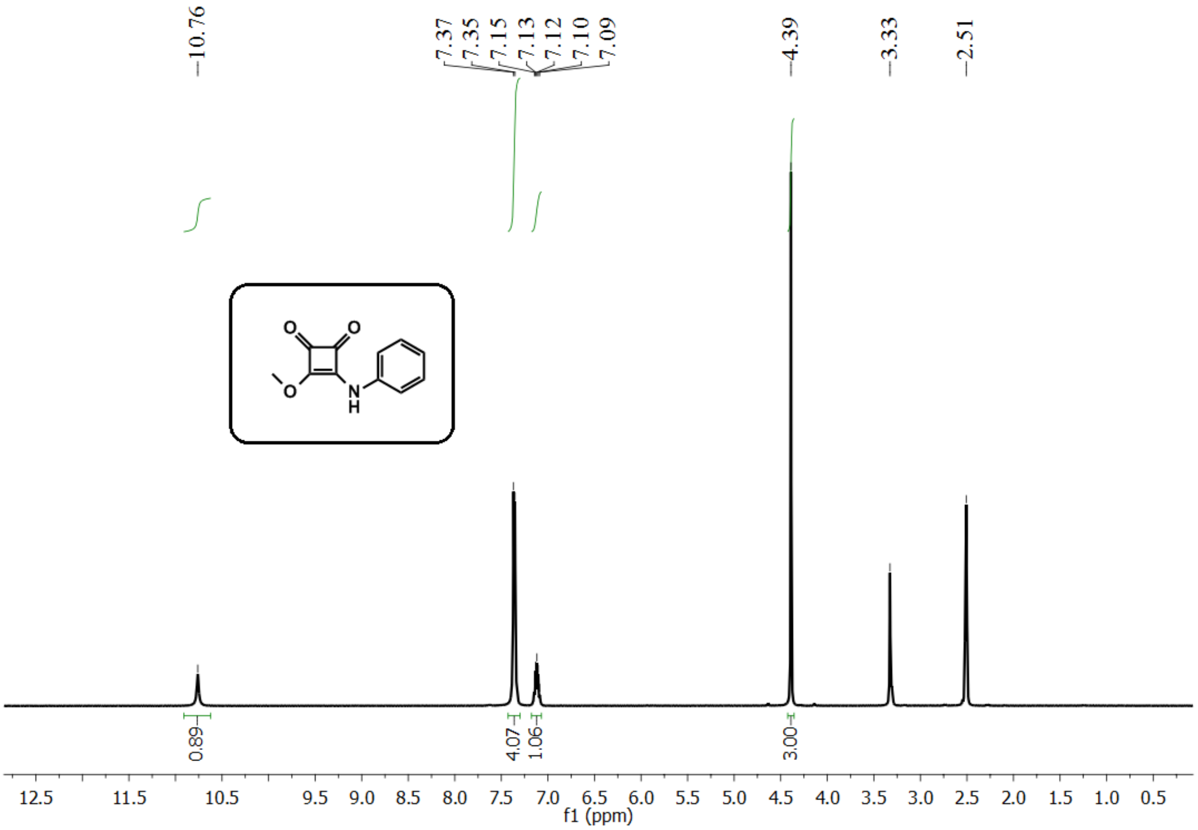
10.76
7.37
7.35
7.15
7.13
7.12
7.10
7.09
4.39
3.33
2.51
0.89
4.07
1.06
3.00
O
O
O
N
H
12.5 11.5 10.5 9.5 9.0 8.5 8.0 7.5 7.0 6.5 6.0 5.5 5.0 4.5 4.0 3.5 3.0 2.5 2.0 1.5 1.0 0.5
f1 (ppm)

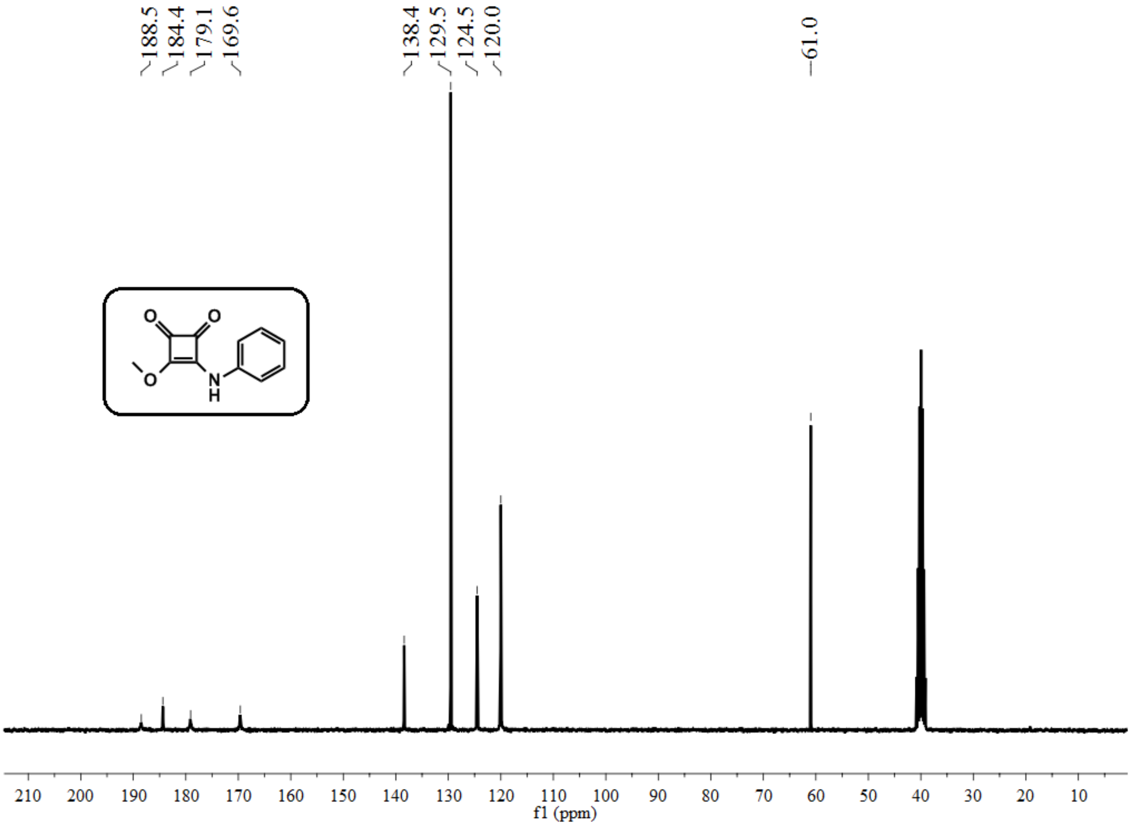
188.5
184.4
179.1
169.6
138.4
129.5
124.5
120.0
61.0
O
O
O
N
H
210 200 190 180 170 160 150 140 130 120 110 100 90 80 70 60 50 40 30 20 10
f1 (ppm)

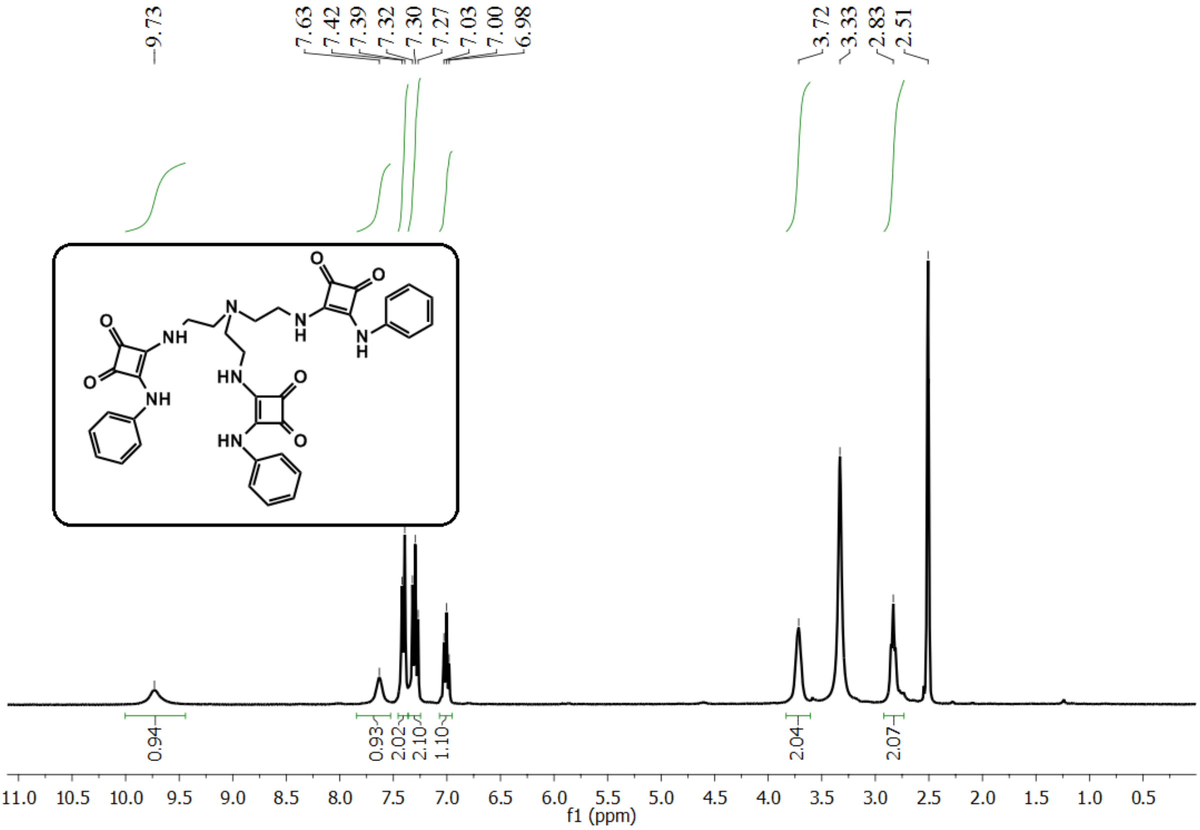
9.73
7.63
7.42
7.39
7.32
7.30
7.27
7.03
7.00
6.98
3.72
3.33
2.83
2.51
0.94
0.93
2.02
2.10
1.10
2.04
2.07
f1 (ppm)

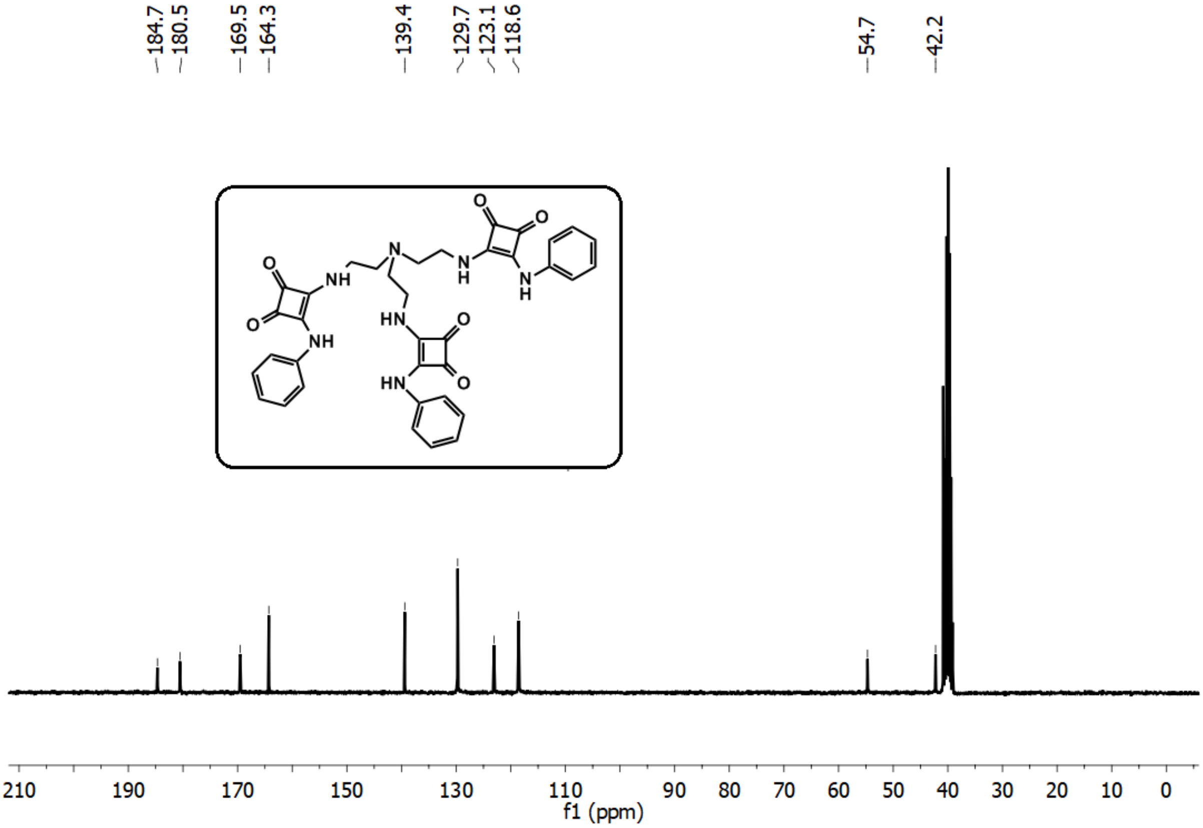
184.7
180.5
169.5
164.3
139.4
129.7
123.1
118.6
54.7
42.2
f1 (ppm)

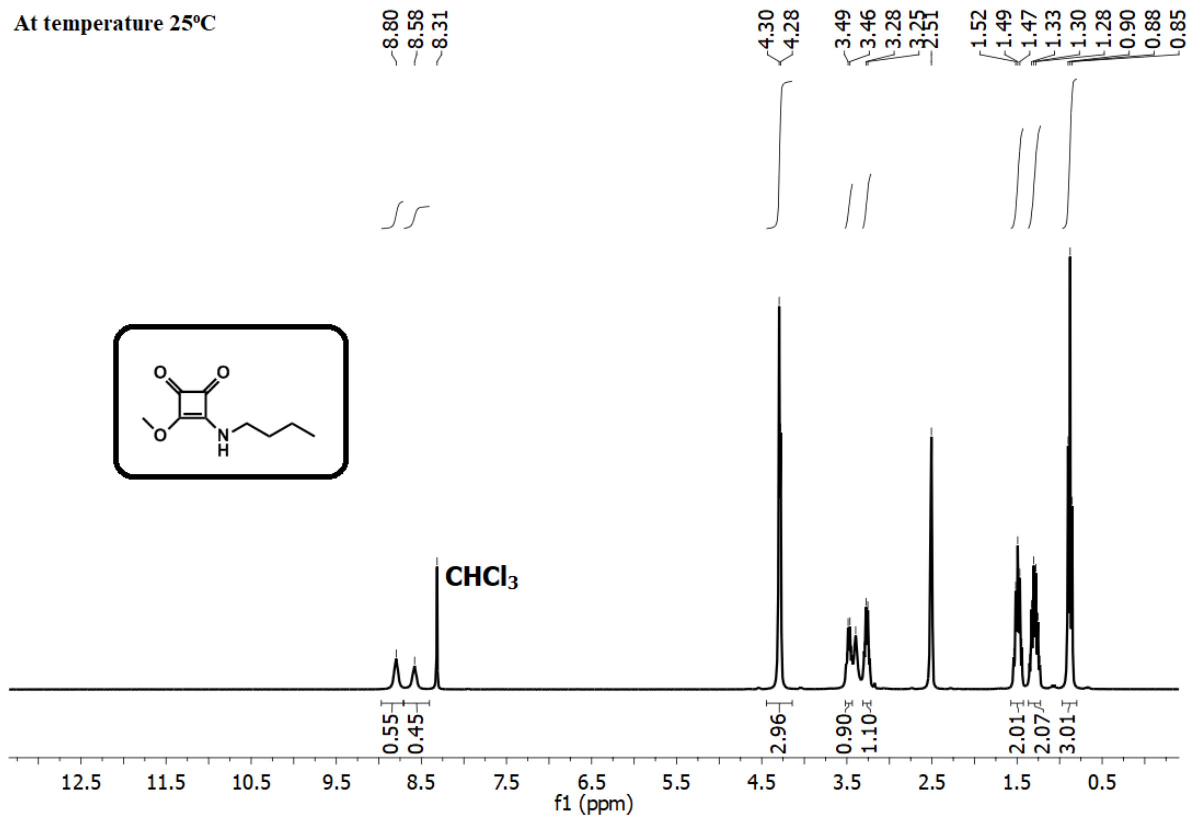
At temperature 25ºC
CHCl3
f1 (ppm)

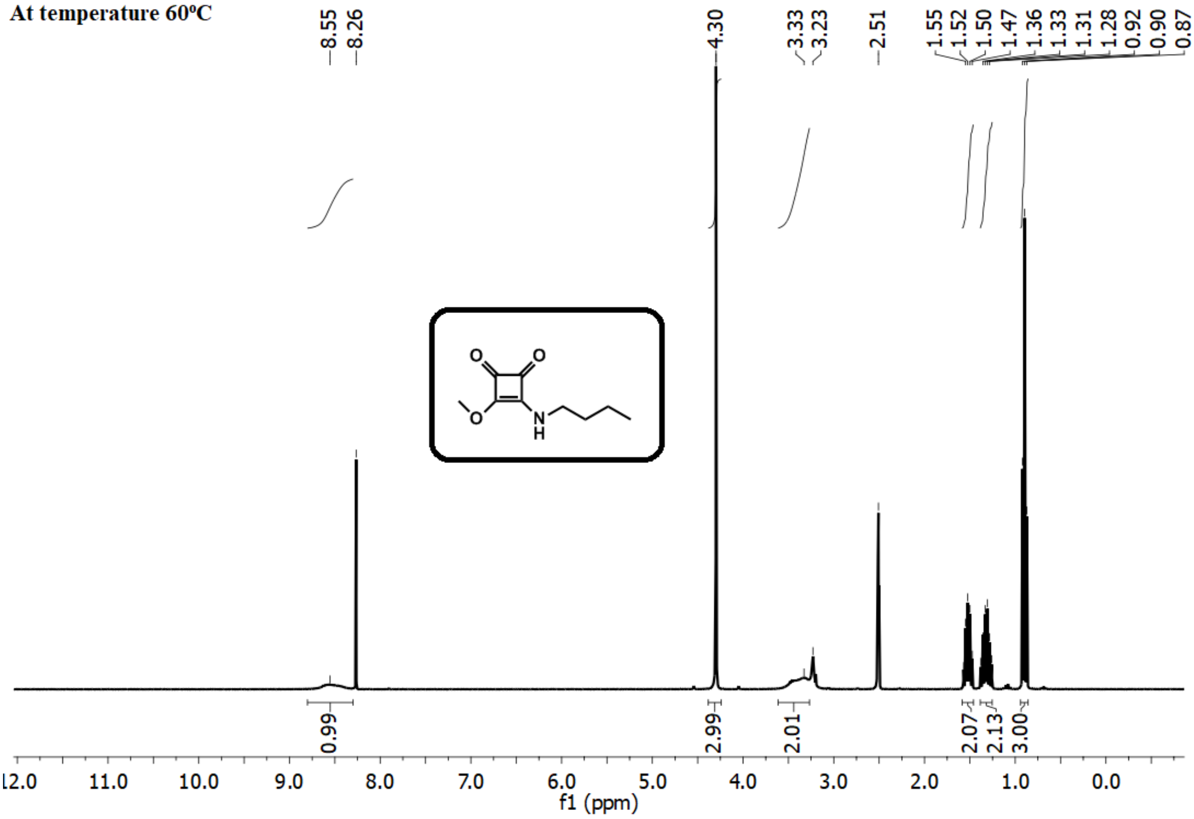
At temperature 60ºC
f1 (ppm)

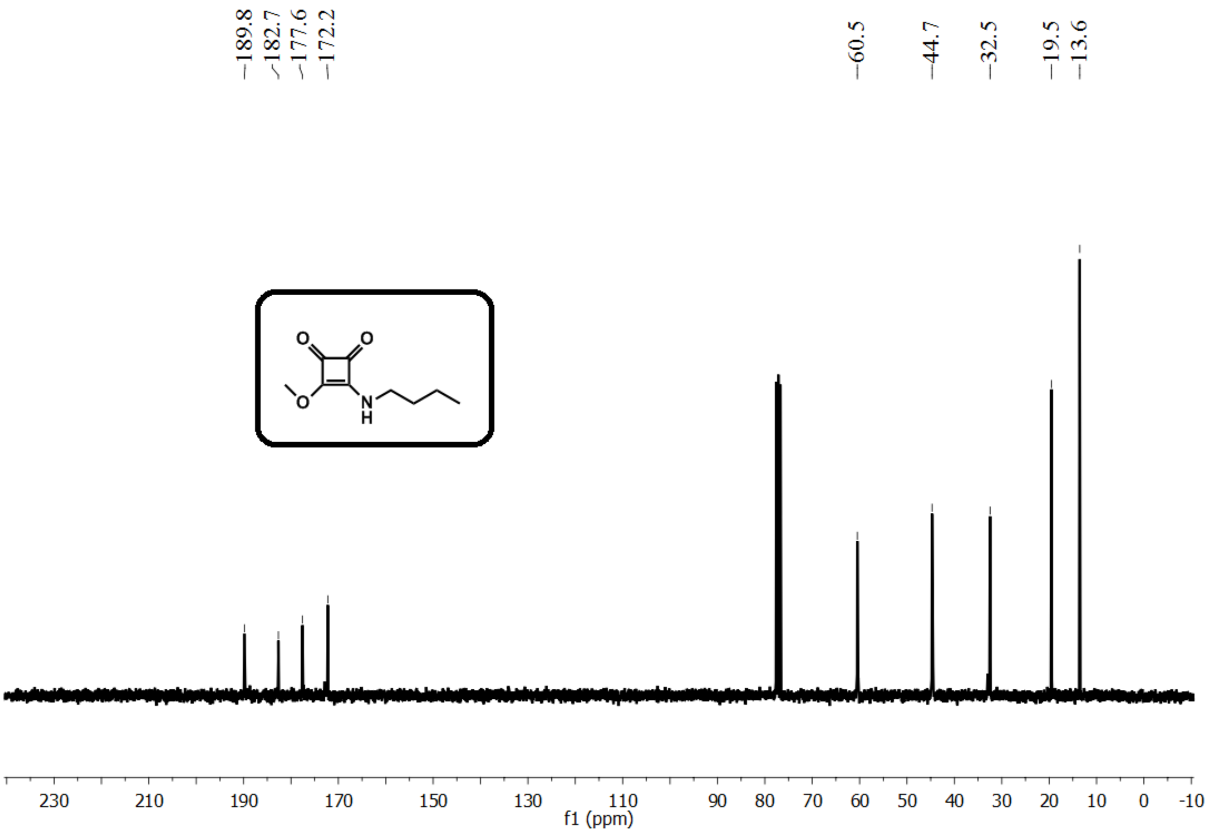
189.8
182.7
177.6
172.2
60.5
44.7
32.5
19.5
13.6
230 210 190 170 150 130 110 90 80 70 60 50 40 30 20 10 0 -10
f1 (ppm)

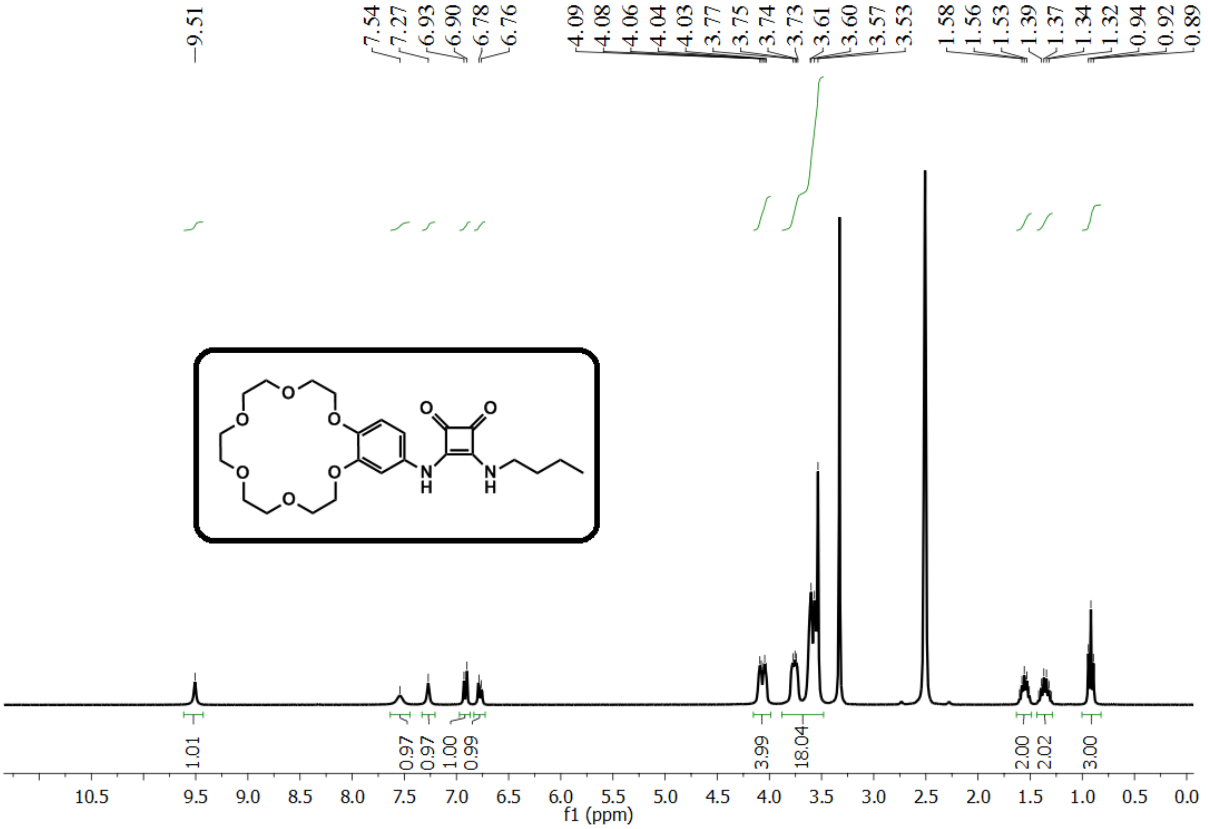
9.51
7.54
7.27
6.93
6.90
6.78
6.76
4.09
4.08
4.06
4.04
4.03
3.77
3.75
3.74
3.73
3.61
3.60
3.57
3.53
1.58
1.56
1.53
1.39
1.37
1.34
1.32
0.94
0.92
0.89
1.01
0.97
0.97
1.00
0.99
3.99
18.04
2.00
2.02
3.00
10.5 9.5 9.0 8.5 8.0 7.5 7.0 6.5 6.0 5.5 5.0 4.5 4.0 3.5 3.0 2.5 2.0 1.5 1.0 0.5 0.0
f1 (ppm)

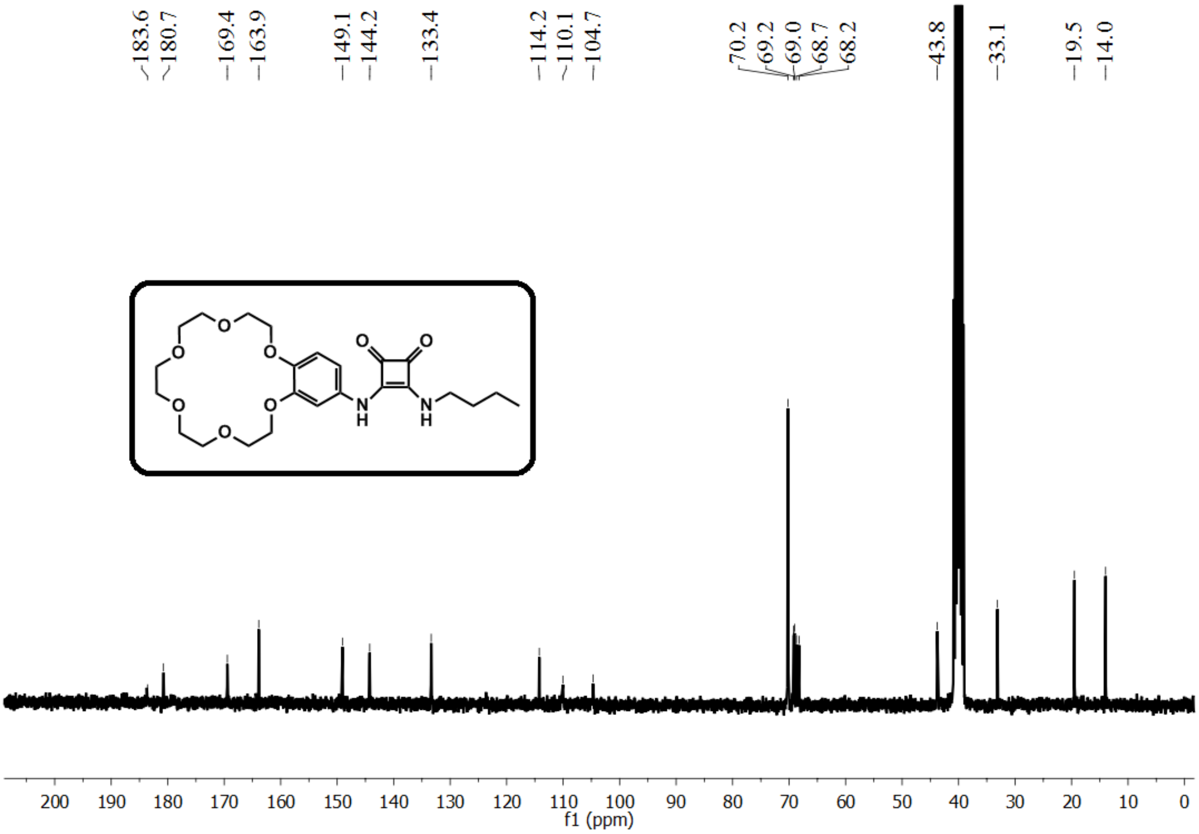
183.6
180.7
169.4
163.9
149.1
144.2
133.4
114.2
110.1
104.7
70.2
69.2
69.0
68.7
68.2
43.8
33.1
19.5
14.0
200 190 180 170 160 150 140 130 120 110 100 90 80 70 60 50 40 30 20 10 0
f1 (ppm)
